# Targeting redox metabolism: the perfect storm induced by acrylamide poisoning in the brain

**DOI:** 10.1038/s41598-019-57142-y

**Published:** 2020-01-15

**Authors:** Demetrio Raldúa, Marta Casado, Eva Prats, Melissa Faria, Francesc Puig-Castellví, Yolanda Pérez, Ignacio Alfonso, Chuan-Yu Hsu, Mark A. Arick II, Natàlia Garcia-Reyero, Tamar Ziv, Shani Ben-Lulu, Arie Admon, Benjamin Piña

**Affiliations:** 1 Institute for Environmental Assessment and Water Research (IDAEA-CSIC), Jordi Girona, 18, 08034 Barcelona, Spain; 2 Research and Development Center (CID-CSIC), Jordi Girona, 18, 08034 Barcelona, Spain; 3 NMR Facility, Institute of Advanced Chemistry of Catalonia (IQAC-CSIC), Jordi Girona, 18, 08034 Barcelona, Spain; 4 Department of Biological Chemistry, Institute of Advanced Chemistry of Catalonia (IQAC-CSIC), Jordi Girona, 18, 08034 Barcelona, Spain; 5 Institute for Genomics, Biocomputing & Biotechnology (IGBB), Mississippi State University, Starkville, MS USA; 6 Environmental Laboratory, US Army Engineer Research and Development Center, Vicksburg, MS USA; 7 Faculty of Biology, Technion-Israel Institute of Technology, Haifa, 32000 Israel

**Keywords:** Metabolomics, Proteomic analysis, Gene expression analysis, Toxicology

## Abstract

Exposure to acrylamide may lead to different neurotoxic effects in humans and in experimental animals. To gain insights into this poorly understood type of neurotoxicological damage, we used a multi-omic approach to characterize the molecular changes occurring in the zebrafish brain exposed to acrylamide at metabolite, transcript and protein levels. We detected the formation of acrylamide adducts with thiol groups from both metabolites and protein residues, leading to a quasi-complete depletion of glutathione and to the inactivation of different components of the thioredoxin system. We propose that the combined loss-of-function of both redox metabolism-related systems configure a perfect storm that explains many acrylamide neurotoxic effects, like the dysregulation of genes related to microtubules, presynaptic vesicle alteration, and behavioral alterations. We consider that our mechanistical approach may help developing new treatments against the neurotoxic effects of acrylamide and of other neurotoxicants that may share its toxic mode of action.

## Introduction

Acrylamide (AA) is a water-soluble type-2 alkene used in paper and textile industries, as a flocculant in the wastewater treatment and municipal drinking water, as a soil conditioner, as a chemical grout in tunnels, sewers and wells, in ore processing, and in cosmetics^1^. Acrylamide has been recognized as mutagenic, carcinogenic^2^, neurotoxic^3,4^, and endocrine disruptor^5^ both in humans and experimental animals. The neurotoxic effects of AA exposure in humans includes ataxia, skeletal muscles weakness, numbness of the extremities, and other symptoms related to polyneuropathy^3,4^. The currently favored hypothesis is that the primary site of action for AA neurotoxic effect is at the presynaptic part of nerve terminals, and that the molecular initiating event of this process is the formation of adducts with specific sulfhydryl thiolate sites from proteins directly involved in the recycling of synaptic vesicles, therefore impairing the synaptic function^6^. Although the loss of synaptic function seems central in the pathophysiology of this condition, additional key events involved in the development of AA neurotoxicity need to be identified.

Within a project to characterize a zebrafish model for studying neurotoxic effects of AA and other neurotoxicants, we recently studied the effects of AA acute exposure in zebrafish adults^7,8^ and larvae^9^. These studies revealed specific behavioral, transcriptomic and metabolic markers compatible with the known effects of AA neurotoxicity in humans and rodents^7–9^. These results pointed to the nervous system as an early target of AA toxicity, indicated by changes in the adult brain proteome and transcriptome, as well as in the neurotransmitter systems, and alterations in the presynaptic vesicles of larval motoneurons^7–9^. However, these data did not allow the identification of the molecular pathways implicated in the observed effects, nor did they define the mechanistic relationships linking these molecular events to the whole-animal toxidrome. In this paper, we use a multi-omic approach to gain insight into the mechanistic aspects of AA toxicity across many levels of biological organization. Our objective was to identify the molecular initiating event (MIE) for AA toxicity and to define the intermediate steps leading to the final behavioral adverse effects. The final purpose of the work is to use this mechanistical understanding for the development of new treatments for the neurotoxic effects of AA and of other neurotoxicants that may share its toxic mode of action.

## Results

### Metabolome analysis

**Figure 1:**
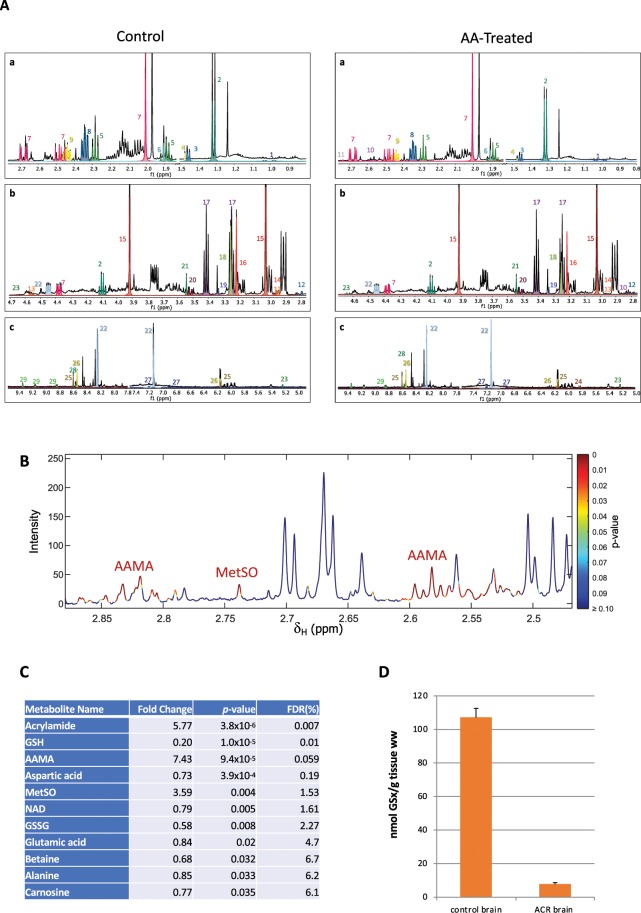
(**A**) ^1^H NMR spectrum of a metabolic extract of control (left) and AA-exposed (right) zebrafish brains. (a) 0.8–2.8 ppm region, (b) 2.8–4.7 ppm region, (c) 4.9–9.5 ppm region. Colors indicated the areas used for integration in the quantitative analysis. Identified metabolites: 1 L-valine, 2 lactic acid, 3 phosphothreonine, 4 L-alanine, 5 GABA, 6 acetic acid, 7 N-acetyl-L-aspartic acid, 8 L-glutamic acid, 9 L-glutamine, 10 AAMA, 11 Met-SO, 12 L-aspartic acid, 13 GSH, 14 GSSG, 15 creatine + P-creatine, 16 choline moiety + GPC, 17 taurine, 18 betaine, 19 scyllo-inositol, 20 myo-inositol, 21 glycine, 22 carnosine, 23 D-glucose, 24 acrylamide, 25 AMP, 26 ATP, 27 L-tyrosine, 28 inosine, 29 NAD^+^. (**B**) Statistical determination of ^1^H NMR signals corresponding to AA, AAMA, and MetSO. Spectral points were colored as a function of *p* values (Student’s T-test). The graph shows a blow-up of an ^1^H NMR spectrum from an AA-exposed brain (2.45–2.87 ppm region). (**C**) List of metabolites with statistically significant changes between control and treated samples. Uncorrected (*p* values, Student’s T-test) and corrected (FDR, %) significance levels are also indicated. (**D**) Biochemical quantitation of the total glutathione (oxidized + reduced) in nmol total GSx/g of brain tissue.

Zebrafish brain metabolome was investigated with one-dimensional proton Nuclear Magnetic Resonance (1D ^1^H NMR) spectroscopy. A detailed spectroscopic analysis of the resonances allowed the characterization of 26 different compounds of the primary metabolism, including amino acids, organic acids, and sugars (Fig. 1A). In addition, different resonances found only in AA-exposed samples were assigned to three non-standard metabolites: free AA, the oxidized form of L-methionine (Met-SO), and AAMA (*N*-acetyl-*S*- (2-carbamoyl-ethyl)-*L*-cysteine), a conjugation product of AA and glutathione found in exposed humans and other vertebrates^10^ (Fig. 1B). AAMA and Met-SO identities were ultimately confirmed by spiking and 2D NMR experiments, as shown in Supplementary Figs. SF1–SF3. All resonance assignments from ^1^H NMR data were verified with 2D NMR spectroscopy (see Methods and Supplementary Table ST1).

Quantitative analysis of the resonance integrals revealed only slight variations upon AA treatment, as only 11 of the identified metabolites showed significative differences between control and AA-treated groups (Fig. 1A–C). The analysis demonstrated that AA, AAMA, and Met-SO accumulated in the brain of treated fish by more than 3-fold, whereas glutathione showed a significant depletion, both in its reduced (GSH) and oxidized (GSSG, glutathione disulfide) forms (Fig. 1A,C, Supplementary Fig. SF2). Biochemical analysis confirmed a dramatic decrease (up to 93%) of total glutathione (GSx) in AA-treated fish compared to controls (Fig. 1D). Other minor changes were found for different amino acids, as well as slight, but significant decreases in NAD^+^, betaine, and carnosine levels (Fig. 1C).

### Protein-acrylamide adduct analyses

**Figure 2:**
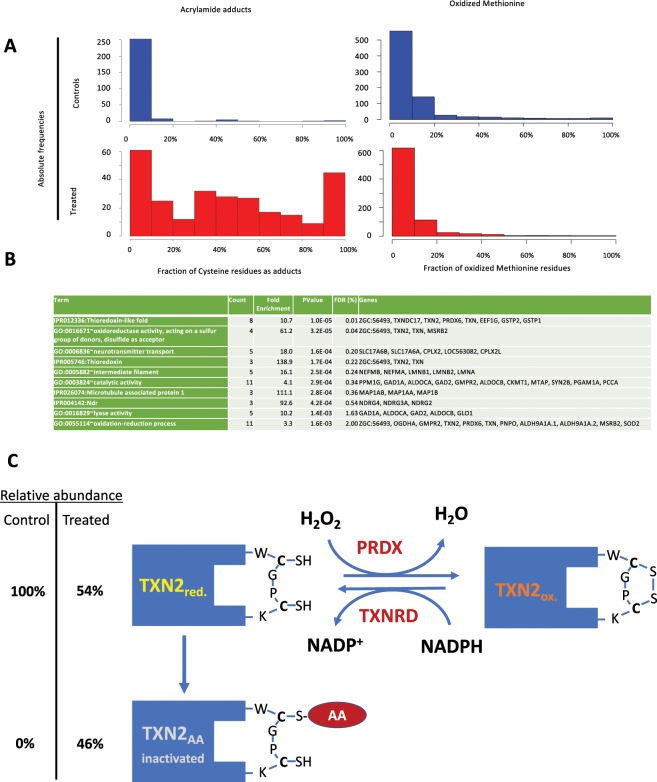
Quantitative analysis of adduct formation in AA-treated zebrafish brain. (**A**) Histograms representing absolute frequency values of cysteine residues showing a given proportion of modification for all Cys-encompassing detected peptides (left) or of oxidized methionine residues (right). Control and treated samples’ histograms are represented in blue and red, respectively. (**B**) Functional analysis of proteins encompassing peptides for which more than 20% of acrylamide adducts have been detected. Redundant functional classes have been discarded, and only those with significant enrichments (less than 5% of false discovery ratio) are represented. (**C**) Graphical representation of the equilibrium between reduced (TXN2_red_), oxidized (TXN2_ox_) and AA-inactivated (TXN2_AA_) forms of the mitochondrial thioredoxin TXN2. Amino acid residues correspond to the actual sequences found in the proteomic analysis, the choice of the blocked cysteine residue is arbitrary. PRDX and TXNRD indicate the enzymes catalyzing the oxidation and reduction of thioredoxin (Peroxiredoxin and thioredoxin reductase, respectively).

In a previous study we reported the presence of propionamide-cysteine adducts, resulting from conjugation of the thiol group with AA, in brain proteins from AA exposed fish^7^. Here, we reanalyzed those proteomic data to quantify the proportion of modified cysteines in AA-treated zebrafish brain proteins by comparing the relative intensities of the corresponding peptide signals. As a result, we identified 385 peptides encompassing at least one Cys residue, 239 of them showing propionamide conjugates in treated samples. No propionamide conjugates were observed in control samples. The histograms in Fig. 2A show that adduct formation differed widely among different peptides, with a relative frequency maximum at around 50% of Cys residues (i.e., peptides showing 50% of Cys residues as adducts) and another maximum at 90–100% of adduct formation in AA-treated samples. Supplementary Table ST2 lists the quantitation results for all detected Cys residues.

DAVID functional analysis of proteins encompassing peptides showing moderate or high levels of acrylamide adducts (from 20 to 100%) identified a short list of functional modules related to either the redox metabolism or to the nervous system structure and function (Fig. 2B). Thioredoxin and thioredoxin-like proteins, together with several oxidoreductases, appeared as significantly enriched among AA-modified peptides (Fig. 2B). Adduct formation was much more relevant for the mitochondrial thioredoxin, TXN2 (46% of observed residues in acrylamide-treated samples) than for the cytoplasmic thioredoxin TXN (20% of observed residues). Proteins related to microtubule function, including the microtubule-associated proteins *map*, and to neurotransmitter transport were also significantly enriched in the adduct-presenting peptide subset (Fig. 2B). We postulate that the formation of AA-adducts with the active thiol groups of TXN2 and, presumably, other thioredoxin domain-encoding proteins should lead to their inactivation, as schematized in Fig. 2C.

Thioredoxin is known to participate in the preservation of the native reduced status of both free- and protein-forming methionine residues^11–13^. We identified 442 peptides encompassing Met residues, from which more than 90% of them presented no or very low proportion of oxidized Met residues (Fig. 2A), and this distribution changed very little (if any) upon AA.

### Transcriptomic analysis

RNA sequence analyses identified 19,034 unique sequences in brain samples. Comparison between control and AA-treated samples showed significant changes in transcripts corresponding to 1,163 zebrafish genes, 176 from which appeared overrepresented and 887 underrepresented in AA-treated samples compared to controls (FDR ≤ 5%, Supplementary Table ST3). Functional analyses showed that overrepresented transcripts were related to transcriptional regulation and/or membrane transport, whereas underrepresented transcripts corresponded to proteins related to main cell functions, like RNA processing and metabolism, transcription, translation, and microtubule assembly, among other categories (Supplementary Table ST4).

**Figure 3:**
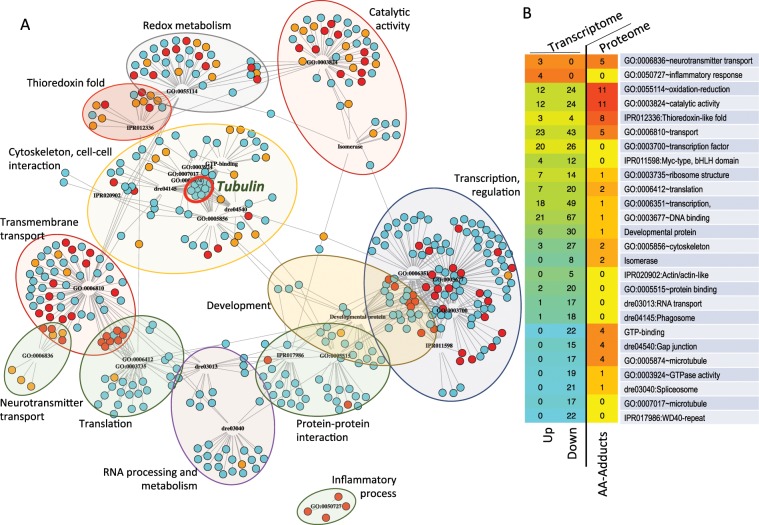
Combined functional analysis of proteomic and transcriptomic data. The dataset included genes whose transcripts were significantly affected by acrylamide treatment (DEGs) plus those encompassing Cys- residues showing a > 20% of propionamide adducts in AA-treated samples. (**A)** Network representation of DEGs and peptides according to their adscription to functional modules (GO:biological process and KEGG databases, codes for each module shown as nodes). DEGs are represented by dots, colored in cyan or red according to their under- or over-representation in AA-treated samples. Orange dots corresponded to proteins for which AA-modified peptides were identified in the proteome analysis (more than 20% of Cys residues modified). Color areas indicate groups of functional modules considered of particular relevance. The cluster of tubulin genes is marked with a red thick circle. (**B**) Distribution of DEGs and of modified peptides among the different functional modules (rows) and their response to acrylamide (columns, “Up” and “Down” for transcripts, “AA-Adducts” for peptides). Only modules with at least 4 hits in at least one of the clusters are represented; redundant modules were simplified to the one with the highest number of hits. Numbers indicate the absolute number of DEGs or peptides for each module and clusters. For transcripts, the shades of red and cyan colors represent the net balance between overrepresented (red) and underrepresented (cyan) genes annotated to each particular functional class. For peptides, cell colors reflect the number of genes in the particular functional class codifying for AA-modified peptides, from yellow (minimum, no genes) to red (maximum) (See Supplementary Table ST2 for further information).

Figure 3 shows a joint network representation of the functional analyses of the modified proteins (orange dots in Fig. 3A), and the over- and under-represented transcripts in AA-treated samples (red and cyan dots, respectively). The graph illustrates the general decrease of transcripts related to the main cell functions (transcription, translation, protein-protein interaction) and a much more complex pattern for membrane function (particularly, transport) and metabolism, with a sizeable proportion of modified proteins in both functional categories (orange dots in Fig. 3A). Particularly relevant are two functional categories intimately related to the nervous system, cytoskeleton and transmembrane transport. With limited exceptions, almost all detected genes related to these categories either were underrepresented in AA-treated samples, or they codified for AA-conjugated peptides (red and orange dots in Fig. 3). Particularly relevant is the block of 17 genes identified as microtubule-related, most of them tubulin genes, which appeared underrepresented in AA-treated samples (labeled with a red circle in Fig. 3A). A similar situation was observed for transcripts/peptides encompassing thioredoxin-fold domains, and, in a lesser extent, for genes/proteins associated to transmembrane transport, redox metabolism and catalytic activity (Fig. 3). We interpret this simultaneous enrichment as an indication that the formation of AA-adducts with protein residues leads to its total or partial inactivation, and that the cell compensates this lack of function by increasing the synthesis of either the same protein or functionally related ones, a classical acclimation response. A quantitative summary of these changes is shown in Fig. 3B.

### Integrated analysis

**Figure 4:**
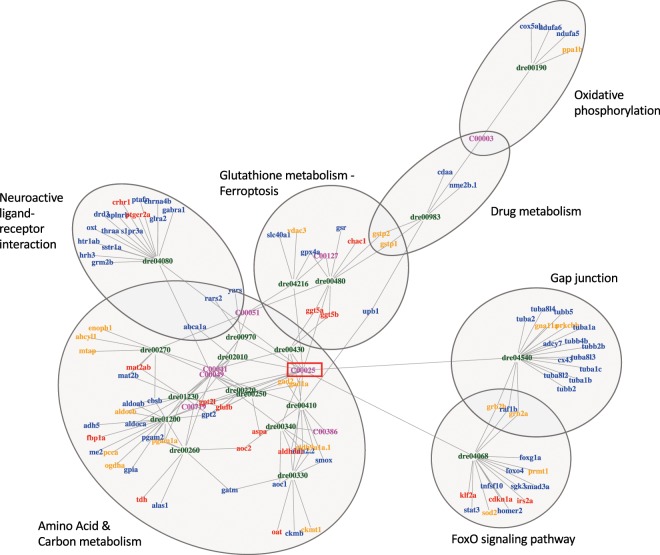
Integrated metabolic, proteomic and transcriptomic functional analysis. The network represents the results from a KEGG pathway analysis of the DEGs (red and blue gene names) and modified proteins (orange gene names) included in Fig. 3, as well as the metabolites listed in Fig. 1C. Only pathways (nodes) including at least one metabolite and five hits are shown. The complete list of KEGG results are shown in Supplementary Table ST3. Gene names are codified as in Fig. 3A: blue and red gene names correspond to genes the transcripts of which appear as under- or over-represented in AA-treated samples, respectively; orange gene names indicate proteins for which at least one peptide showed more than 20% of its Cys residues as propionamide adducts. KEGG codes (in magenta) represent the metabolites listed in Fig. 1C; only natural metabolites could be included in the KEGG analysis, and all of them were found underrepresented in the AA-treated metabolome (Fig. 1C). KEGG codes are the following: C00003, NAD^+^; C00025, L-Glutamate; C00041, L-Alanine; C00049, L-Aspartate; C00051, GSH; C00127, GSSG; C00386, Carnosine; C00719 Betaine. Ovals show the position of relevant pathways. The central position of L-Glutamate, which participated in most identified pathways, is marked with a red frame.

The correlations between metabolomic, proteomic and transcriptomic results were studied at the functional level, using the KEGG pathway analysis (https://www.genome.jp/, Fig. 4, Supplementary Table ST5). In this analysis, only metabolic pathways including at least one of the metabolites listed in Fig. 1C was considered. Several of the identified pathways coincided with or were related to functional categories identified by DAVID (Fig. 3), like neuroactive ligand-receptor interaction or gap junction pathways. In addition, the analysis identified some pathways related to amino acid and glutathione metabolism, indicating that these pathways were affected at metabolomic, transcriptomic and proteomic level. Two other pathways, Ferroptosis and FoxO signaling pathway, are related to different stressors, including oxidative stress^14,15^, and they could be related to the observed deregulation of functional modules related to main cell functions (transcription, translation, protein-protein interaction) observed in Fig. 3. Note the central role of L-Glutamate (red frame in the center in Fig. 4), which participates in many of the identified pathways (see also Supplementary Table ST3). The analysis suggests a general depression of many of these pathways, as most of their associated transcripts appear underrepresented in AA-treated samples (blue gene names in Fig. 4). This is less evident for pathways related to the general metabolism (amino acids, carbon-metabolism), which present a significant proportion of over-represented transcripts (red gene names in Fig. 4). We interpret this as reflecting a mixture of inhibitory and compensatory mechanisms induced to cope with the loss of function of AA-adducted enzyme molecules (codifying gene names in orange in Fig. 4).

## Discussion

Zebrafish are being increasingly used in the development of animal models of human neurotoxic syndromes. Its nervous system shares many features with terrestrial vertebrates, including humans, from the overall structure to neurotransmitter complement and functions. In previous papers, we developed animal models of AA acute neurotoxicity in adult and larval zebrafish^7–9^. Since the construct validity of the model developed in adult has already been tested^7,8^, we consider that the toxicity mechanisms defined in this study may apply to other vertebrate species, including humans.

The use of NMR spectroscopy for characterizing metabolomic alteration by endogenous or exogenous processes has the unique advantage of giving the opportunity to identify and quantify metabolites or toxicant derivatives that could not be foreseen at the beginning of the analysis^16–18^. In this work, we detected three non-physiological compounds in the brain of AA-treated zebrafish: AA itself, the oxidized form of L-methionine (Met-SO), and the glutathione-acrylamide adduct derivative AAMA. The accumulation of the latter is likely related to the observed major depletion of GSH and GSSG. It should be pointed out that this is, to our knowledge, the first report of accumulation of AAMA in a tissue, as it is normally detected as an excretion product in AA-exposed humans and rodents^19,20^. We think that this may be due to the presence of the blood-brain barrier and the predicted inability of AAMA to cross it (BBB predictor tool, www.cbligand.org/AD/, data not shown).

The formation of AA-derived thiol adducts (propionamide adducts) was also observed in peptidic cysteine residues in the proteomic analysis, particularly in proteins of the thioredoxin family (thioredoxin-fold) and in proteins related to different brain functions. We propose that the formation of propionamide adducts in cysteine residues inactivates many of the affected proteins, and particularly thioredoxin, as at least some of the modified residues lie on the active site. Therefore, the AA action would ultimately result in the simultaneous inactivation of the two major cellular systems for regenerating oxidized thiol groups, the glutathione and the thioredoxin systems, a particularly damaging effect for the nervous system^21^. The main enzymes responsible for elimination of hydrogen peroxide (H_2_O_2_) in cells are peroxiredoxins (PRxs), catalase, and glutathione peroxidases (GPxs)^22^. The catalytic reduction of H_2_O_2_ by Prx and GPx involves the oxidation of catalytic thiol groups on selenocysteine or cysteine residues in GPx and cysteine residues in Prx, and the recycling of these enzymes lies on the thioredoxin and glutathione system, respectively^23^. In AA-treated animals, with the glutathione and the thioredoxin systems strongly compromised, only catalase should be available for H_2_O_2_ reduction. Whereas catalase activity has not been evaluated in this study, no up-regulation of catalase has been found at the transcript or protein level in AA-treated animals, so we can predict that catalase activity will not be enough for an efficient reduction of H_2_O_2_ in the cells, and in particular in the mitochondria. In these conditions, H_2_O_2_ levels should be steady increasing. As H_2_O_2_ is an important intracellular messenger, increased H_2_O_2_ levels will result initially in changes in some cellular functions, including proliferation, differentiation or migration^23^, and finally in oxidative stress and cellular damage.

The integrated metabolic, proteomic and transcriptomic analysis suggest a major disruption of the central carbon and amino acid metabolism pathways in AA-treated animals, as well as of some neural-specific functions, like the GAP function or the neuroactive ligand-receptor interaction pathway. This metabolic distortion may well be related to the observation that AA-treated animals show a depletion in the monoamine neurotransmitters, which is consistent with the observed alterations in behavior linked to AA exposure^7,24,25^ (see below).

**Figure 5:**
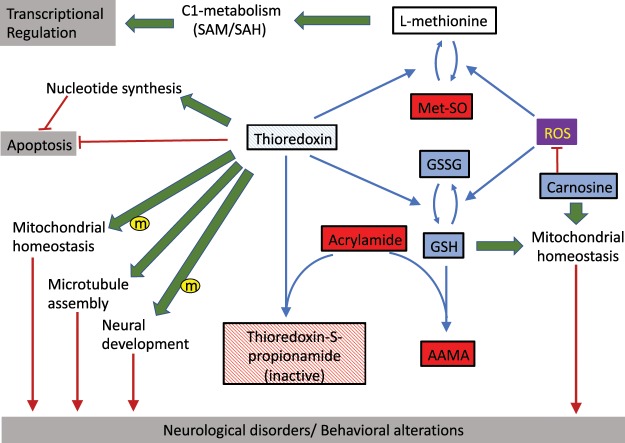
Functional intercorrelation of different metabolites and their relation with cellular functions linked to acrylamide-elicited brain disorders. Blue and red boxes represent metabolites that become enriched or depleted in AA-treated brain. White boxes indicate metabolites for which no quantification was possible in the analysis. Analogously, boxes thioredoxin and its propionamide adduct follow the same color code, indicating the formation of adducts and the decrease of free, functional thioredoxin. Blue arrows indicate direct metabolic relationships, green arrows indicate indirect functional or regulatory ones. Red lines ending with a dash indicate an inhibitory effect, red arrows indicate those cell functions whose disruption lead to neurological disorders. Small case “m” in a yellow circle indicates effects specifically described for the mitochondrial thioredoxin.

The metabolic and functional relationships between the changes identified in the multi-omic analysis and the proposed inactivation of the glutathione and thioredoxin is summarized in Fig. 5. The figure shows the oxidation of free L-methionine by reactive oxygen species (ROS), leading to the accumulation of Met-SO (metabolic relationships are represented by blue lines). This effect is likely favored by the depletion of carnosine in AA-treated animals, a recognized neuron protector from oxidative stress^26^. A lower susceptibility to the oxidation, a highest efficiency of the repair methionine sulfoxides or a combination of both factors could explain the low proportion of oxidized methionine residues found in proteins compared with the free methionines. Interestingly, all the methionine sulfoxide reductases expressed in zebrafish (msrb1a, msrb1b, msrb2, msrb3) belong to the MSRB, a class of MSR repairing with a high efficiency the protein-based methionine-R-sulfoxide, but with low efficiency in repairing the free methionine-R-sulfoxide^27^. The figure also shows the likely mechanism underlying the almost complete depletion of GSH and GSSG by the conversion of the former into AAMA upon AA adduct formation. The proposed inactivation of the thioredoxin system would prevent the regeneration of both methionine from its oxidized form^28^, and the reduction of GSSG to GSH^29^. Thioredoxin is also required for the microtubule assembling process^11,30^ and for the functioning of nucleotide reductases involved in nucleotide synthesis^21^. There are several reports linking functional thioredoxin to neural development and protection^12^, microtubule assembly^11^, and, together with glutathione, to the maintenance of mitochondrial homeostasis^31^. These functional relationships are represented by green arrows in Fig. 5. Glutathione and carnosine are necessary factors to maintain mitochondrial homeostasis, which is also dependent on mitochondrial thioredoxin activity (marked with an “m” in a yellow circle in the Fig. 5). Thioredoxin has also been related to play a central role on blocking apoptotic signals^32^ (marked with red lines in Fig. 5). Particularly relevant is the role of thioredoxin on C1 cell metabolism, by maintaining the levels of native (i.e., non-oxidized) cytoplasmic methionine, which is a key metabolite in the SAM/SAH cycle. SAM is the main methyl group donor in the cell, participating in the modification of residues in nucleic acids and proteins, including histone modifications, with a direct impact in transcriptional regulation. Red arrows in Fig. 5 indicate how the disruption of several of these processes (mitochondrial homeostasis, microtubule assembly, and neuronal development) have been directly related to neurological disorders.

Many of the effects of the depletion of glutathione and thioredoxin systems can be traced as changes in the AA-treated brain transcriptome. For example, our results indicate a major disruption of microtubule-related function, as mRNAs coding for 15 out of the 27 genes annotated as Tubulin in INTERPRO (IPR000217) for zebrafish appeared underrepresented in treated brain samples (Supplementary Table ST4). This would be consistent with the effects reported for AA exposure in human and other vertebrates^33–35^, and probably linked to the lack of function of thioredoxin, and particularly of the mitochondrial form, TXN2, which has been identified as essential for neuronal maintenance and energy metabolism in humans.

**Figure 6:**
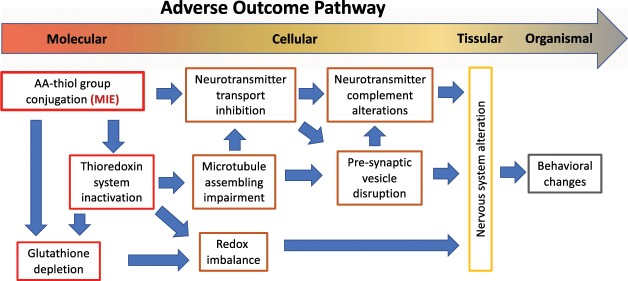
Adverse Output Pathway analysis of the proposed AA mode of toxicity. The graph shows the different potentially adverse effects detected at molecular (transcriptome, metabolome), cell (metabolome, immunohistochemistry), tissue, and organism (behavior) levels of organization. The molecular initiating event (MIE) is indicated at the upper left end corner of the scheme. Blue arrows indicate the proposed cause/effect relationships.

The links between the proposed MIE and the different key-events involved in AA acute neurotoxicity are summarized in the tentative Adverse Outcome Pathway (AOP) analysis^36^ for AA neurotoxicity shown in Fig. 6. We propose that the formation of adducts of acrylamide with thiol groups of metabolites and proteins is the molecular initiating event (MIE) of AA neurotoxicity. This process leads to the virtually complete elimination of glutathione and to the inactivation of thioredoxin and thioredoxin-like proteins. We consider that this dual inactivation deprives cells of their two major mechanisms for controlling it redox status and repairing ROS damage^29,37^. On the other hand, thioredoxin inactivation may compromise microtubule assembling^11,30^, which in turn may affect neurotransmitter transport. This particular function likely suffers from the direct inactivation of some of its components by the formation of AA adducts, and it is an evident candidate for playing a substantial role in the observed changes previously reported in behavior and in the neurotransmitter profile in the AA-treated zebrafish brain^7,8^. It may also be related to the observed alteration of pre-synaptic vesicles in AA-treated zebrafish larvae^9^, an effect not yet observed in adult zebrafish, but that is mechanistically related to one of the proposed mode of action of AA neurotoxicity in humans^6^. The model finally proposes that the alteration on neurotransmitter transport, microtubule assembling and redox imbalance ends affecting the nervous system of the affected animals, leading to the different abnormal behaviors (ataxic gait and comorbid depression and anxiety disorder) observed in AA-treated zebrafish adults and larvae^7–9^.

In summary, our results unravel a new and highly relevant molecular pathway involved the AA acute neurotoxicity, the disruption of the main mechanism of the cell for controlling redox homeostasis, glutathione, and thioredoxin system, configuring a “perfect storm” that triggers a cascade of potentially catastrophic effects at cellular and tissue levels. It is unclear which part of the observed toxicity is due to the direct inactivation of cysteine thiol groups from active centers of enzymes and structural proteins, and which part is linked to the inability of the cell to revert protein methionine oxidation in the absence of a functional thioredoxin system. A similar dual impact on both glutathione and thioredoxin systems has been reported for methylmercury (MeHg)^38^. MeHg not only induces GSH depletion^39^ but also binds to the thiol groups of at least two thioredoxin forms (Trx1 and TrxR) inhibiting their function, a proposed major molecular mechanism of mercury toxicity^38^. Another recognized neurotoxicant, the herbicide paraquat, also impairs the thioredoxin system, but through a different mechanism, depleting the cellular pool of NADPH, the ultimate electron donor used by the thioredoxin system^21,40^. AA is considered as highly toxic in account of its potential carcinogenic effects; our results, combined with different previous observations and studies, indicate that efforts to limit public exposure to it, or to counteract accidental, suicidal, or provoked acrylamide intoxications, should also focus on its neurotoxic effects.

## Materials and Methods

### Zebrafish experiments

Wild-type zebrafish were obtained from Piscicultura Superior (Barcelona, Spain) and maintained in fish water (reverse-osmosis purified water containing 90 mg/L Instant Ocean (Aquarium Systems, Sarrebourg, France), 0.58 mM CaSO4·2H_2_O) at 28 ± 1 °C under a 12 L:12D photoperiod. Adult zebrafish (≈50:50 male:female ratio) were either exposed to 0.75 mM AA (exposed, Sigma-Aldrich, St. Louis, MO) or maintained in fish water (controls) for 72 h. Exposure and control media were renewed after 48 h. Fish were euthanized by a hypothermic shock in ice-chilled water (2° to 4 °C), and brains were immediately excised, pooled (4 brains/sample), and stored at −80 °C for further analyses. All procedures were approved by the Institutional Animal Care and Use Committees at the CID-CSIC and conducted in accordance with the institutional guidelines under a license from the local government (agreement number 9027).

### NMR metabolomics

#### Metabolite extraction

The extraction protocol was adapted from Puig-Castellví *et al*. (2015)^18^. Tissue samples were freeze-dried and homogenized in 1500 μl of a solution of cold CHCl_3_:MeOH (2:1) using a TissueLyser (Qiagen, 2 stainless steel beads, 50 Hz, 2 min). Next, 350 μL of cold milliQ water were added and the biphasic system was mixed at 4 °C for 20 min by orbital shaking. After centrifugation (16500 rpm, 4 °C, 20 min), the aqueous phase was collected and transferred to a 2-mL eppendorf tube. The extraction process was repeated once. The two supernatants were combined and freeze-dried, and the resulting dried residue was stored at −80 °C for further analyses.

#### NMR sample preparation

The extract was dissolved in 700 μl of deuterated phosphate buffer (25 mM Na_2_DPO_4_, pH 7.0 in D_2_O) with 0.2 mM DSS as internal standard. After centrifugation at 13,000 rpm for 5 min at 4 °C, the resulting solution was placed into the NMR tube.

#### NMR spectroscopy

NMR spectra were recorded at 298 K on a Bruker Avance III console (Bruker Biospin, Germany) combined with a 9.7 T Oxford Instruments magnet (^1^H 500 MHz), equipped with a 5 mm inverse cryogenically cooled triple-resonance TCI (^1^H, ^13^C, ^15^N, and ^2^H lock) cryoprobe with a z-axis magnetic field-gradient capability. The instrument also had a Bruker SampleXpress™ System for sample delivery and an ATMA unit (Bruker Biospin, Germany) for automated tuning and matching. The samples were run under automation by IconNMR and the acquired data were processed using Topspin 3.6

#### ^1^H NMR experiments

For every tube, the sample was locked to the solvent, tuned and matched, shimmed and the optimal 90° pulse was calculated using the automated routine. The 90° length value and the saturation frequency and power (presaturation power corresponding to a 25 Hz field) for each sample and the rest of fixed acquisition parameters (see below) were used for the 1D NOESY acquisition. For every sample, the optimal water saturation and the line width at half height of the DSS (which should be less than 0.7 Hz with no line broadening) were checked. For each brain tissue extract (5 samples from untreated zebra fish and 5 samples from zebra fish exposed to acrylamide), one ^1^H NMR NOESY-presat (Bruker library - noesygppr1d) spectrum was acquired in automation.

#### 2D NMR experiments

For one of the extracts of each group (untreated/exposed to acrylamide), an ensemble of 2D experiments were acquired (^1^H J-resolved, COSY, TOCSY and ^1^H-^13^C HSQC; acquisition parameters in Supplementary Material).

#### Preprocessing of NMR spectra

NMR spectra have been automatically referenced, phased and baseline corrected using TopSpin (Bruker BioSpin GmbH, Billerica, MA, USA) routines. Moreover, the ^1^H NMR spectra were apodized with a weighting exponential function of 0.3 Hz with MestreNova v.11.0 (Mestrelab Research, Spain), and imported to Matlab R2016a (The Mathworks Inc., Natick, MA, USA) as a spectral data matrix. Then, regions of 4.68–5.14 ppm (water), 7.65–7.68 ppm (chloroform), below 0.7 ppm (DSS), and above 9.5 ppm (empty) were removed. After the removal of these spectral regions, the number of data-points for each ^1^H NMR spectra was 28,913. Minor resonance misalignments were corrected with *icoshift*^41^. Finally, the processed ^1^H NMR spectra were normalized using the Probabilistic Quotient Normalization (PQN) method^42^.

#### Statistical analysis of the ^1^H NMR spectra

Significant differences in the ^1^H NMR spectra were screened by the application of a Student’s t-test on each set of intensities for every chemical shift.

#### Metabolite identification

Metabolite assignment was performed by a detailed analysis of the ^1^H NMR, ^1^H-J Resolved, ^1^H-^1^H TOCSY/COSY and ^1^H-^13^C HSQC NMR of the extracts’ spectra using the Human Metabolome Data Base library^43^, the NMR spectral library BBIOREFCODE alongside the Analytical Profiler tool in AMIX software (both from Bruker Biospin Ltd.) and the Biological Magnetic Resonance Data Bank^44^ **(**BMRB, http://www.bmrb.wisc.edu/). The presence of Met-SO and AAMA was ultimately checked by spiking using commercial standards (Met-SO, Alfa Aesar, Thermo Fisher, Karlsruhe, Germany; AAMA, Santa Cruz Biotechnology, Inc. Dallas, TX, USA)

#### Integration of ^1^H NMR resonances

Relative metabolite quantifications of the ^1^H NMR spectral matrix were performed using BATMAN R-package^18,45^.

#### Statistical analysis of the resonance integrals

Significant metabolic concentration changes in the zebrafish metabolome were studied by one-way ANOVA. All metabolomic data would be available on request.

### Proteome analysis

The proteomic raw data used for the present study were obtained from Faria *et al*.^7^, The relative proportions of native and modified peptides (propionamide adducts and oxidized methionine residues) were quantified using the MaxQuant software, based on extracted ion currents (XICs). Unmodified cysteine residues were quantified as carbamidomethyl derivatives, due to the processing of proteomic samples (see Supplementary Table ST2). Areas from all biological replicates, each one corresponding to 10 pools of three brains each (five control and five treated) were averaged for the analysis. The mass spectrometry proteomics data can be acceded from the ProteomeXchange Consortium via the PRIDE partner repository with the dataset identifier PXD008993. Relevant methodological information from our previous work has been included as Supplementary Material.

### Total glutathione measurement

Brain tissue was homogenized in 5% ice cold trichloroacetic acid (TCA), prepared in 0.1 mM Phosphate Buffer pH 7.4 (200 mg/mL w/v). The homogenates were then centrifuged at 4 °C and 12 000 rpm for 5 min and the supernatant was collected for total glutathione (GSx) measurement^46^. Total glutathione content (GSx = GSH + 2xGSSG) was determined in a Synergy 2 Multi-Mode Microplate Reader (BioTek Instruments − Vermont, USA) using the DTNB assay as described^46,47^. The final concentration of reagents was 0.21 mM NADPH, 0.6 mM DTNB and 0.175 U/mL GR in the presence of 0.05 mM triethanolamine prepared in phosphate buffer. Triethanolamine stabilizes pH to 6–7 to avoid autoxidation of reduced glutathione (GSSG). GSSG was used as GSx standard, where 1 µM of GSSG is equivalent to 2 µM of GSx^46^. Standards were prepared and measured under the same conditions as samples. Samples and standards were incubated for 30 minutes at 25 °C to allow the formation of the GSx-TNB complex and were then measured at 412 nm. Total GSx in samples was extrapolated using the standard curve and final results were expressed as nmol GSx/g tissue ww (wet weight).

### RNAseq

#### RNA extraction

Frozen samples were homogenized with Trizol (Invitrogen) in a TissueLyser (Qiagen) with stainless steel beads. After centrifugation, the upper aqueous phase was used to isolate RNA according to the manufacturer’s instructions. Subsequently, the lower organic phase was processed to isolate the protein fraction according to the manufacturer’s instructions. Pellets containing the proteins were kept in 70% cold acetone for proteomics analysis. Quality parameters for the 14 brain RNA samples (seven from controls and seven from AA-treated animals) are listed in the Supplementary Table ST6.

#### RNA-Seq library preparation

The total RNA samples were treated with DNase I (Promega, Madison, Wisconsin, USA) and cleaned up by using Qiagen RNeasy Mini Kit (Qiagen, Valencia, California, USA) to remove the contaminated genomic DNA in the RNA samples. The concentration of total RNA from each sample was determined by Nanodrop 1000 Spectrophotometer (NanoDrop Technologies, Wilmington, Delaware, USA) and the quality was confirmed with Fragment Analyzer Automated CE System (Agilent, formerly Advanced Analytical Technologies, Santa Clara, California, USA) using High Sensitivity RNA Analysis Kit. Each stranded mRNA-Seq library was constructed from 500 ng of total RNA by using the TruSeq Stranded mRNA Sample Preparation HT Kit (Illumina, San Diego, California, USA) based on the manufacturer’s protocol. All library samples were independently barcoded with Illumina TruSeq Dual Indexes (Illumina, San Diego, California, USA) for multiplexing. The concentration and size of each RNA-Seq library were validated with Qubit fluorometer using Qubit dsDNA HS Assay Kit (Life Technologies, Grand Island, NY) and Fragment Analyzer Automated CE System using dsDNA 935 Reagent Kit (Agilent, formerly Advanced Analytical Technologies, Santa Clara, California, USA), respectively. These stranded mRNA-Seq libraries were sequentially pooled together with equal volume from equal concentration and sequenced with paired-end 150 bp (PE150, or 2 × 150) Illumina NovaSeq6000 System (Illumina, San Diego, California, USA). Parameters of the sequencing procedures are listed in the Supplementary Table ST6.

#### Bioinformatics

Salmon (v0.9.1)^48^ generated transcript expression estimates for each library by mapping the raw Illumina RNA-seq data to the *D*. *rerio* transcriptome (vGRCz11)^49^. Gene expression estimates were produced by combining all transcript estimates from the same gene^50^. Genes with low expression (an average log2 transformed count per million less or equal to one) were removed from further analysis^51^. The gene expression estimates were normalized based on the sequencing depth of each library. EdgeR (v3.12.1)^52^ was used to find differentially expressed genes. Generalized linear models were used to test for differential expression based on contrasting the acrylamide treatment against the control. Genes with an FDR adjusted p-value ≤ 0.05 were considered differentially expressed. The sequencing data have been archived in the NCBI Short Read Archive with the BioProject accession number PRJNA515927.

### Functional analyses

Gene enrichment analysis was performed in a combined transcriptomic/proteomic dataset using DAVID Bioinformatic Resources 6.8 (https://david.ncifcrf.gov), using the default zebrafish (*Danio rerio*) as background. The datased included all DEGs and all proteins for which the extend of Cys modification exceeded the 20% for at least one of its identified peptides. Enrichment significances were set to a false discovery ratio (FDR) ≤ 5%. Identified modules with at least five hits were included in the network analysis, using the *reshape2* and *igraph* packages in R^53^. Any two given genes were considered linked if they share at least one common KEGG or GO (Gene Ontology) module. Correlations between metabolomic, proteomic and transcriptomic results at the functional level were explored using the KEGG pathway analysis (https://www.genome.jp), adding the metabolites identified as significantly altered by AA-treatment in the metabolomic analyses to the combined transcriptomic/proteomic dataset. Note that the KEGG pathway analysis tool does not calculate enrichment values or significance values. Only natural metabolites could be included in the analysis. Network analyses were performed using the same *reshape2* and *igraph* packages in R.

## Supplementary information

Supplementary Information

## References

[1] Friedman M (2003). Chemistry, biochemistry, and safety of acrylamide. A review. Journal of agricultural and food chemistry.

[2] IARC (1994). Monographs on the Evaluation of Carcinogenic Risks to Humans: Acrylamide.

[3] Fujita A (1960). Clinical observation on 3 cases of acrylamide intoxication. Nippon. Iji. Shimpo..

[4] Auld RB, Bedwell SF (1967). Peripheral neuropathy with sympathetic overactivity from industrial contact with acrylamide. Canadian Medical Association Journal.

[5] Matoso V, Bargi-Souza P, Ivanski F, Romano MA, Romano RM (2019). Acrylamide: A review about its toxic effects in the light of Developmental Origin of Health and Disease (DOHaD) concept. Food Chemistry.

[6] LoPachin RM, Gavin T (2012). Molecular mechanism of acrylamide neurotoxicity: lessons learned from organic chemistry. Environmental health perspectives.

[7] Faria M (2018). Acrylamide acute neurotoxicity in adult zebrafish. Scientific reports.

[8] Faria M (2019). Further characterization of the zebrafish model of acrylamide acute neurotoxicity: gait abnormalities and oxidative stress. Scientific reports.

[9] Prats E (2017). Modelling acrylamide acute neurotoxicity in zebrafish larvae. Scientific reports.

[10] Boettcher MI, Schettgen T, Kütting B, Pischetsrieder M, Angerer J (2005). Mercapturic acids of acrylamide and glycidamide as biomarkers of the internal exposure to acrylamide in the general population. Mutation Research - Genetic Toxicology and Environmental Mutagenesis.

[11] Landino LM, Skreslet TE, Alston JA (2004). Cysteine oxidation of tau and microtubule-associated protein-2 by peroxynitrite - Modulation of microtubule assembly kinetics by the thioredoxin reductase system. Journal of Biological Chemistry.

[12] Silva-Adaya Daniela, Gonsebatt María E., Guevara Jorge (2014). Thioredoxin System Regulation in the Central Nervous System: Experimental Models and Clinical Evidence. Oxidative Medicine and Cellular Longevity.

[13] Matsuzawa A (2017). Thioredoxin and redox signaling: Roles of the thioredoxin system in control of cell fate. Archives of Biochemistry and Biophysics.

[14] Lei P, Bai T, Sun Y (2019). Mechanisms of Ferroptosis and Relations With Regulated Cell Death: A Review. Front Physiol.

[15] Klotz LO (2015). Redox regulation of FoxO transcription factors. Redox. Biol..

[16] Dunn WB, Broadhurst DI, Atherton HJ, Goodacre R, Griffin JL (2011). Systems level studies of mammalian metabolomes: the roles of mass spectrometry and nuclear magnetic resonance spectroscopy. Chemical Society Reviews.

[17] Puig-Castellvi, F., Alfonso, I., Pina, B. & Tauler, R. H-1 NMR metabolomic study of auxotrophic starvation in yeast using Multivariate Curve Resolution-Alternating Least Squares for Pathway Analysis. *Scientific Reports* **6**, 10.1038/srep30982 (2016). 10.1038/srep30982 PMC4971537 27485935

[18] Puig-Castellvi F, Alfonso I, Pina B, Tauler R (2015). A quantitative H-1 NMR approach for evaluating the metabolic response of Saccharomyces cerevisiae to mild heat stress. Metabolomics.

[19] Boettcher MI, Angerer J (2005). Determination of the major mercapturic acids of acrylamide and glycidamide in human urine by LC–ESI-MS/MS. Journal of Chromatography B.

[20] Luo Y-S (2015). Synthesis, characterization and analysis of the acrylamide-and glycidamide-glutathione conjugates. Chemico-biological interactions.

[21] Ren X (2017). Redox signaling mediated by thioredoxin and glutathione systems in the central nervous system. Antioxidants & redox signaling.

[22] Rhee SG, Yang KS, Kang SW, Woo HA, Chang TS (2005). Controlled elimination of intracellular H(2)O(2): regulation of peroxiredoxin, catalase, and glutathione peroxidase via post-translational modification. Antioxid. Redox. Signal.

[23] Veal EA, Day AM, Morgan BA (2007). Hydrogen peroxide sensing and signaling. Mol. Cell.

[24] Dixit R, Husain R, Mukhtar H, Seth PK (1981). Effect of acrylamide on biogenic amine levels, monoamine oxidase, and cathepsin D activity of rat brain. Environmental research.

[25] Kyzar E (2013). Behavioral effects of bidirectional modulators of brain monoamines reserpine and d-amphetamine in zebrafish. Brain research.

[26] Cheng J, Wang F, Yu D-F, Wu P-F, Chen J-G (2011). The cytotoxic mechanism of malondialdehyde and protective effect of carnosine via protein cross-linking/mitochondrial dysfunction/reactive oxygen species/MAPK pathway in neurons. European journal of pharmacology.

[27] Lee BC, Dikiy A, Kim H-Y, Gladyshev VN (2009). Functions and evolution of selenoprotein methionine sulfoxide reductases. Biochimica et Biophysica Acta (BBA)-General Subjects.

[28] Stadtman ER, Van Remmen H, Richardson A, Wehr NB, Levine RL (2005). Methionine oxidation and aging. Biochimica et Biophysica Acta (BBA)-Proteins and Proteomics.

[29] Gromer S, Urig S, Becker K (2004). The thioredoxin system—from science to clinic. Medicinal research reviews.

[30] Khan IA, Luduen RF (1991). Possible regulation of the *in vitro* assembly of bovine brain tubulin by the bovine thioredoxin system. Biochimica et Biophysica Acta (BBA)-Protein Structure and Molecular Enzymology.

[31] Go, Y.-M., Fernandes, J., Hu, X., Uppal, K. & Jones, D. P. Mitochondrial network responses in oxidative physiology and disease. *Free Radical Biology and Medicine* (2018). 10.1016/j.freeradbiomed.2018.01.005 PMC5833979 29317273

[32] Saitoh M (1998). Mammalian thioredoxin is a direct inhibitor of apoptosis signal-regulating kinase (ASK) 1. Embo. Journal.

[33] Yu S (2006). Acrylamide alters cytoskeletal protein level in rat sciatic nerves. Neurochemical research.

[34] Zhang L, Gavin T, DeCaprio AP, LoPachin RM (2010). γ-Diketone axonopathy: analyses of cytoskeletal motors and highways in CNS myelinated axons. Toxicological Sciences.

[35] Chauhan NB, Spencer PS, Sabri MI (1993). Effect of acrylamide on the distribution of microtubule-associated proteins (MAP1 and MAP2) in selected regions of rat brain. Molecular and chemical neuropathology.

[36] Ankley GT (2010). Adverse outcome pathways: a conceptual framework to support ecotoxicology research and risk assessment. Environmental Toxicology and Chemistry.

[37] Townsend DM, Tew KD, Tapiero H (2003). The importance of glutathione in human disease. Biomedicine & Pharmacotherapy.

[38] Carvalho CM, Chew E-H, Hashemy SI, Lu J, Holmgren A (2008). Inhibition of the human thioredoxin system a molecular mechanism of mercury toxicity. Journal of Biological Chemistry.

[39] Sarafian T, Verity MA (1991). Oxidative mechanisms underlying methyl mercury neurotoxicity. International Journal of Developmental Neuroscience.

[40] Wu B (2012). Central nervous system damage due to acute paraquat poisoning: a neuroimaging study with 3.0 T MRI. Neurotoxicology.

[41] Savorani F, Tomasi G, Engelsen S (2010). B. icoshift: A versatile tool for the rapid alignment of 1D NMR spectra. Journal of Magnetic Resonance.

[42] Dieterle F, Ross A, Schlotterbeck G, Senn H (2006). Probabilistic quotient normalization as robust method to account for dilution of complex biological mixtures. Application in 1H NMR metabonomics. Analytical chemistry.

[43] Wishart DS (2017). HMDB 4.0: the human metabolome database for 2018. Nucleic acids research.

[44] Ulrich EL (2008). BioMagResBank. Nucleic Acids Research.

[45] Hao J (2014). Bayesian deconvolution and quantification of metabolites in complex 1D NMR spectra using BATMAN. Nature protocols..

[46] Baker, M. A., Cerniglia, G. J. & Zaman, A. Microtiter plate assay for the measurement of glutathione and glutathione disulfide in large numbers of biological samples. *Anal Biochem*. **190**, 360–365, doi:0003-2697(90)90208-Q [pii] (1990). 10.1016/0003-2697(90)90208-q 2291479

[47] Pena-Llopis S, Pena J, Sancho E, Fernandez-Vega C, Ferrando M (2001). Glutathione-dependent resistance of the European eel Anguilla anguilla to the herbicide molinate. Chemosphere.

[48] Patro R, Duggal G, Love MI, Irizarry RA, Kingsford C (2017). Salmon provides fast and bias-aware quantification of transcript expression. Nature methods.

[49] Warren WC (2010). The genome of a songbird. Nature.

[50] Soneson, C., Love, M. I. & Robinson, M. D. Differential analyses for RNA-seq: transcript-level estimates improve gene-level inferences. *F1000Research* **4** (2015). 10.12688/f1000research.7563.1 PMC4712774 26925227

[51] Bourgon R, Gentleman R, Huber W (2010). Independent filtering increases detection power for high-throughput experiments. Proceedings of the National Academy of Sciences.

[52] Robinson MD, McCarthy DJ, Smyth G (2010). K. edgeR: a Bioconductor package for differential expression analysis of digital gene expression data. Bioinformatics.

[53] R_Development_Core_Team. *R: A language and environment for statistical computing*, http://www.R-project.org (2008).

