# Supplementary material for: Targeting redox metabolism: the perfect storm induced by acrylamide poisoning in the brain
Source: Sci Rep. 2020 Jan 15;10:312. doi: 10.1038/s41598-019-57142-y (PMC6962170; doi:10.1038/s41598-019-57142-y)
Supplement: Supplementary file 1 — Supplementary Information [file 41598_2019_57142_MOESM1_ESM.pdf]

Supplementary Information

Targeting redox metabolism: the perfect storm induced by acrylamide poisoning in the brain

Demetrio Raldúa, Marta Casado, Eva Prats, Melissa Faria, Francesc Puig-Castellví, Yolanda Pérez, Ignacio Alfonso, Chuan-Yu Hsu, Mark A. Arick II, Natàlia Garcia-Reyero, Tamar Ziv, Shani Ben-Lulu, Arie Admon, Benjamin Piña

Supplementary information for proteome analysis

Proteomic Analysis

Protein fraction were extracted from 5 pools (of 3 brains each) from control group and 5 pools (of 3 brains each) from AA-treated adult zebrafish. Proteins were trypsinized and 2ug of tryptic peptides from each samples analyzed by LC-MS/MS using a Q-Exactive-Plus mass spectrometer fitted with a capillary HPLC.

Proteomic data Analysis

The mass spectrometry data were analyzed using the MaxQuant software 1.5.2.8 (www.maxquant.org) (Cox & Mann, 2008) fitted with the Andromeda (Cox et al., 2011) search engine searching against the *Danio rerio* Uniprot database (of March 2017 containing 59,064 entries) with mass tolerance of 20 ppm for the precursor masses and the fragment ions. Oxidation on methionine, propionamide on cysteine, histidine and lysine, and carbamidomethyl on cysteine was accepted as variable modifications. Minimal peptide length was set to six amino acids and a maximum of two miscleavages was allowed. Peptide and protein level false discovery rates (FDRs) were filtered to 1% using the target-decoy strategy. The identified protein table was filtered to remove the identifications from the reverse database, the common contaminants and single peptide identifications.

Data were quantified by normalized label free analysis using the same MaxQuant software (LFQ intensities), based on extracted ion currents (XICs) of peptides enabling quantitation from each LC/MS run for each peptide identified in any experiment. Ratio mod/base was calculated for each samples by the MaxQuant software.

Cox, J. & Mann, M. MaxQuant enables high peptide identification rates, individualized ppb-range mass accuracies and proteome-wide protein quantification. *Nat. Biotechnol.* **26**, 1367 (2008).

Cox, J. *et al.* Andromeda: a peptide search engine integrated into the MaxQuant environment. *J. Proteome Res.* **10**, 1794–1805 (2011).

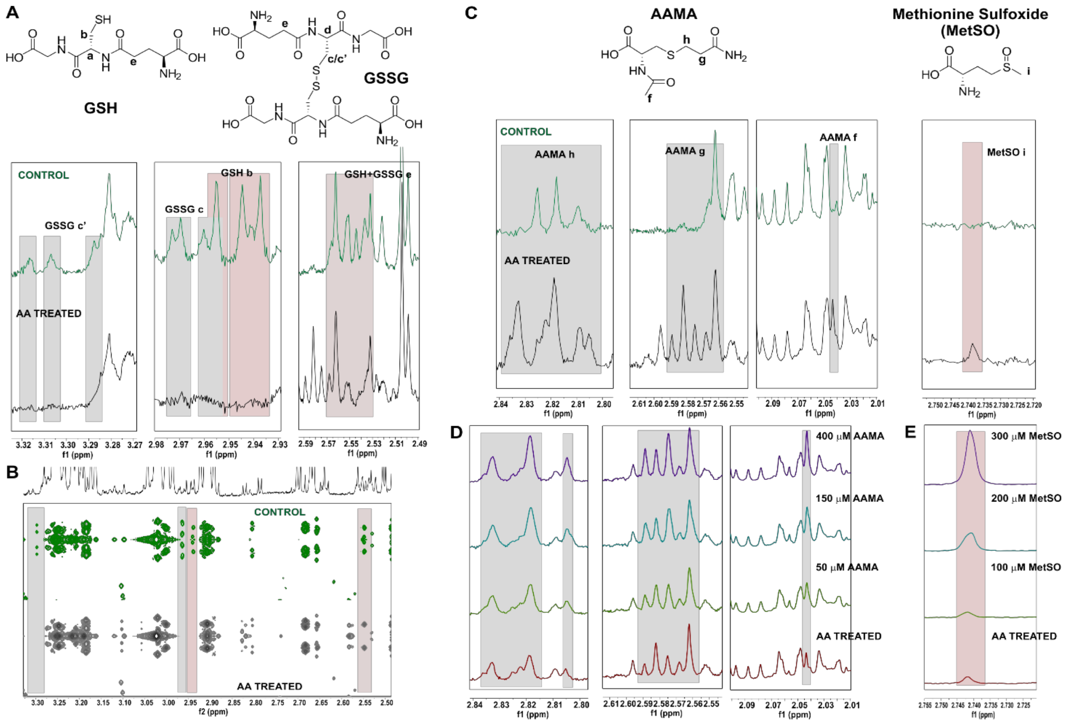

Supplementary Figure SF1

Supplementary Figure SF1. (A) Partial ^{1}H NMR spectra of zebrafish brain extract (green trace) and treated with AA (black trace) showing diagnostic signals for the identification of the reduced and oxidized forms of glutathione (signal assignment in the upper chemical structures). (B) J-RES NMR spectra for the corresponding untreated (green) and AA-treated (black) of zebrafish brain extracts. (C) Partial ^{1}H NMR spectra of zebrafish brain extract (green trace) and treated with AA (black trace) showing diagnostic signals for the identification of AAMA and MetSO. (D) Spiking experiments for the confirmation of the presence of AAMA. (E) Spiking experiments for the confirmation of the presence of MetSO.

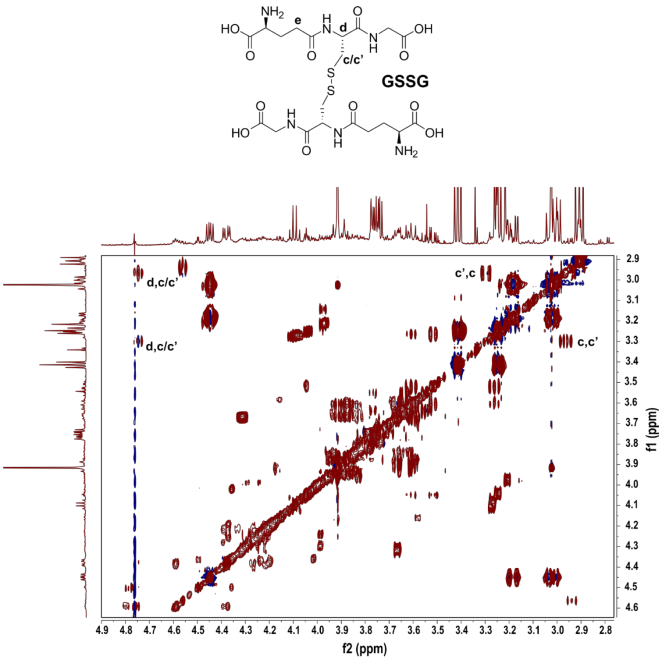

Supplementary Figure SF2

Supplementary Figure SF2. ^{1}H-^{1}H COSY experiment showing representative signals of oxidized glutathione (GSSG) observed in zebrafish brain extracts. Relevant GSSG resonances are indicated by low-case letters, which correspond to the positions indicated in the molecular formula represented on the top of the figure.

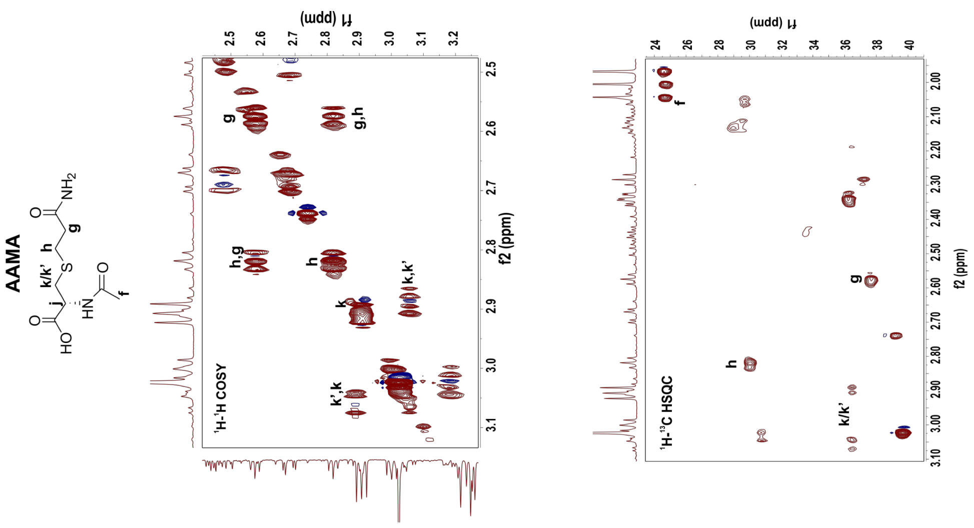

Supplementary Figure SF3

Supplementary Figure SF3. ^{1}H-^{1}H COSY (up) and ^{1}H-^{13}C HSQC (down) experiments to confirm AAMA assignment. The graphs show results from a ACR-treated zebrafish brain extract spiked with 0.5mM AAMA. Relevant AAMA resonances are indicated by low-case letters, which correspond to the positions indicated in the molecular formula represented on the top of the figure.

Supplementary Table ST1. Proton, carbon and *J*-coupling values for low molecular weight brain zebrafish metabolites. Chemical shifts are reported with reference to DSS (at 0.00 ppm) and multiplicity definitions are s (singlet), d (doublet), t (triplet), dd (doublet of doublets) and m (multiplet).

#	Metabolite	Group	^{1}H/ppm	Peak intensity/area	^{1}H-^{1}H correlations	Confirmed in COSY/TOCSY?	^{13}C/ppm	Confirmed in HSQC?	Confirmed in JRES ?	observed ^{1}H multiplicity	J_{HH} (in Hz)	Notes
1	**N-Acetyl-S-(carbamoylethyl)-L-cysteine**											Derived form adduct between GSH and AAM. Confirmed by spiking
	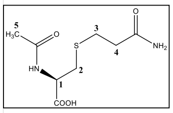	1 - CH	4.36	tiny	n.o.	no	n.o.	no	no	n.o.	n.m.	Partially overlapped with N-Acetyl-L-Aspartic Ac.
		2 1H-CH_2	3.06	tiny	n.o.	no	n.o.	no	yes	dd	13.66, 4.32	Partially overlapped with Creatine and Carnosine
		2 1H-CH_2	2.89	tiny	n.o.	no	n.o.	no	yes	dd	13.61, 8.00	Partially overlapped with DSS
		3 -CH_2	2.82	small	2.58-2.82 (COSY)	yes	n.o.	no	yes	dd	6.99	One of the J's is very small; overlapped with L-Aspartic Ac. in treated brain samples
		4 -CH_2	2.58	small		yes	n.o.	no	yes	dd	7.00	One of the J's is very small
		5 -CH_3	2.04	small			n.o.	no	yes	s		
2	**Acrylamide** 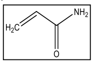	CH	5.81	tiny	6.22-5.81	yes	n.o.	no	yes	dd	10.01, 1.47	
		1H-CH_2	6.22	tiny	6.22-5.81	yes	n.o.	no	no	n.o.	n.m.	
		1H-CH_2	6.28	tiny	n.o.	no	n.o.	no	no	n.o.	n.m.	
3	**ATP** 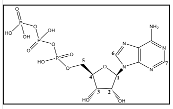	1 - CH	6,14	medium	6.14-4.79 (TOCSY)<br>6.14-4.59 (TOCSY)<br>6.14-4.39 (TOCSY)	yes	6.14-89.26	yes	yes	d	5.78	Anomeric sugar protons from nucleosides: ATP+AMP+Inosinic Acid
		2 -CH	4.80*	n.o.	6.13-4.80 (COSY)	yes	n.o.	no	no	n.o.		Below water supressed resonance
		3 -CH	4.39	tiny	4.59-4.39 (TOCSY)	yes	n.o.	no	yes	m		
		4 -CH	4.59	tiny	4.59-4.39 (TOCSY)	yes	n.o.	no	no	n.o.		
		5 -CH_2	4.21-4.28	tiny	4.39-4.21 (TOCSY)	yes	n.o.	no	no	n.o.		
		6 -CH	8.52***	medium	n.o.	no	n.o.	no	yes	s		
		7 -CH	8,26	medium	n.o.	no	8.25-155.65	yes	yes	s		Overlapped with AMP
4	**AMP** 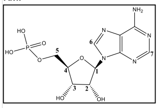	1 - CH	6,13	small	6.13-4.50 (TOCSY)<br>6.13-4.80 (TOCSY)	yes	6.14-89.26	yes	yes	d	5.85	
		2 -CH	4.80*	n.o.	6.13-4.80 (COSY)	yes	n.o.	no	no	n.o.		Below water supressed resonance
		3 -CH	4.50	small	4.50-4.36 (TOCSY)	yes	n.o.	no	yes	m		
		4 -CH	4.36	small	4.36-4.50 (TOCSY)	yes	n.o.	no	no	n.o.		Overlapped with N-acetyl-L-aspartic acid and Inosinic acid
		5 -CH_2	4.02*	n.o.	n.o.	no	n.o.	no	no	n.o.		
		6 -CH	8.58***	medium	n.o.	no	n.o.	no	yes	s		Overlapped with Inosinic Acid
		7 -CH	8.25	medium	n.o.	no	8.25-155.65	yes	yes	s		Overlapped with ATP
5	**Betaine** 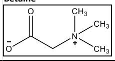	- $(CH_3)_3N+$	3.25	medium	3.25-3.89 (COSY)	yes	3.25-56.15	yes	yes	s		Overlapped with D-glucose
		- CH_2	3.89	small			3.89-69.03			s		Overlapped with L-Aspartic Acid
6	**Carnosine** 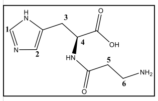	1 -CH	8.22	large	8.22-7.12 (COSY)	yes	8.22-136.85	yes	yes	d	2.40	
		2 -CH	7.12	large		yes	7.12-119.83	yes	yes	d	2.72	
		3 1H-CH_2	3.19	medium	3.19-3.02 (TOCSY)	yes	3.19-30.83	yes	yes	dd	15.10, 5.00	Overlapped with Choline/Phosphorylcholine
		3 1H-CH_2	3.02	medium	3.02-3.19 (TOCSY)	yes	3.02-30.83	yes	yes	dd	15.20, 8.50	Overlapped with GABA and Creatine
		4 -CH	4.45	medium	4.45-3.18 (COSY)<br>4.45-3.02 (COSY)	yes	4.45-57.24	yes	yes	dd	8.40, 4.80	
		5 -CH_2	3.22*	n.m.	n.o.	no	n.o.	no	m	m	n.m.	Overlapped with GPC
		6 -CH_2	2.68*	n.m.	n.o.	no	n.o.	no	m	m	n.m.	Overlapped with N-Acetyl-L-Aspartic Ac. and L-Aspartic Ac.
7	**Choline+PC+ GPC (Choline moiety)** 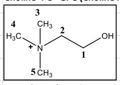	1 -CH_2	4.06	tiny	n.o.	no	n.o.	no	no	n.o.	n.m.	Tiny resonance, overlapped with myo-inositol
		2 -CH_2	3.52*	n.o.	n.o.	no	n.o.	no	no	n.o.	n.m.	Tiny resonance, overlapped with myo-inositol
		3,4,5 -$(CH_3)_3N+$	3.22	large	n.o.	no	3.22-56.72	yes	yes	s		Assigned to Glycerophosphocholine; Overlapped with Carnosine
			3.19	small	n.o.	no	3.19-56.64	yes	yes	s		Assigned to Choline/Phosphorylcholine
	**GPC Glycerol moeity** 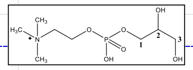	1 -CH_2	3.94/3.88	tiny	n.o.	no	3.94/3.88-69.27	yes	no	n.o.	n.m.	Overlapped with Creatine/Betaine
		2 -CH	3.91	tiny	n.o.	no	3.91-73.31	yes	no	n.o.	n.m.	Overlapped with Creatine/Betaine
		3 -CH_2	3.66/3.60	tiny	n.o.	no	3.66/3.60-64.90	yes	no	n.o.	n.m.	Overlapped with Myo-inositol
8	**Creatine + Phosphocreatine** 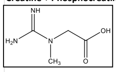	- CH_3	3.02	very large	3.92-3.02 (COSY)	yes	3.92-56.61	yes	yes	s		
		- CH_2	3.92	very large		yes	3.02-39.66	yes	yes	s		
9	**4-Aminobutyric Ac. (GABA)** 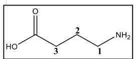	1 -CH_2	3.01	medium	3.01-1.89 (COSY)<br>3.01-2.28 (TOCSY)	yes	3.01-42.03	yes	yes	t	7.58	Overlapped with Carnosine
		2 -CH_2	1.89	medium	2.28-1.89 (COSY)	yes	2.28-37.14	yes	yes	q	7.36	Overlapped with (possible) acetic acid
		3 -CH2	2.28	medium		yes	1.89-26.32	yes	yes	t	7.42	Overlapped with L-Valine (very tiny)
10	**Glucose**	Alpha-D-Glucose										
	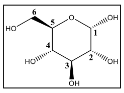	1- CH	5.22	tiny	n.o.	no	n.o.	no	yes	d	4.3	
		2 -CH	3.53	tiny	n.o.	no	3.53-75.10	yes	no	n.o.	n.m.	Overlapped with Myo-inositol
		3 -CH	3.70	tiny	n.o.	no	n.o.	no	no	n.o.	n.m.	Overlapped with L-Glutamic Acid and L-Glutamine
		4 -CH	3.41	tiny	n.o.	no	n.o.	no	no	n.o.	n.m.	Overlapped with Taurine
		5 -CH	3.84	tiny	n.o.	no	n.o.	no	no	n.o.	n.m.	Overlapped with L-Methionine
		6 -CH_2	3.81/3.73	tiny	n.o.	no	n.o.	no	no	n.o.	n.m.	Overlapped with L-Glutamic Acid and L-Glutamine
		Beta-D-Glucose										
	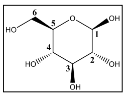	1- CH	4.65	tiny	n.o.	no	n.o.	no	yes	d	8.4	
		2 -CH	3.26	tiny	n.o.	no	3.26-77.01	yes	yes	n.o.	8.3	Overlapped with Myo-inositol and Taurine
		3 -CH	3.47	tiny	n.o.	no	n.o.	no	no	n.o.	n.m.	Overlapped with Myo-inositol
		4 -CH	3.41	tiny	n.o.	no	n.o.	no	no	n.o.	n.m.	Overlapped with Myo-inositol
		5 -CH	3.47	tiny	n.o.	no	n.o.	no	no	n.o.	n.m.	Overlapped with Myo-inositol
		6 -CH_2	3.88/3.73	tiny	n.o.	no	n.o.	no	no	n.o.	n.m.	Overlapped with L-Methionine
11	**Glutathione** 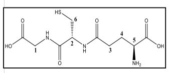	2 -CH	4.56	tiny	4.56-2.94 (COSY)	yes	n.o.	no	yes	dd	7.20, 5.10	
		3 -CH_2	2.54	tiny	2.54-2.15 (COSY)	yes	2.54-34.16	yes	yes	m	n.m.	Overlapped with GSSG
		4 -CH_2	2.15	tiny	2.15-2.54 (TOCSY)	yes	n.o.	no	yes	dd	8.43,13.55	Overlapped with L-Glutamic Accid, L-Glutamine and L-Methionine
		5 -CH ; 1 -CH_2	3.77	tiny	3.77-2.15 (COSY)<br>3.77-2.54 (TOCSY)	yes	3.77-46.20<br>3.77-56.88	yes	no	n.o.	n.m.	Overlapped with alpha-CH from L-Glutamic Acid, L-Glutamine and D-Glucose
		6 -CH_2-SH	2.94	tiny	2.94-4.56 (TOCSY)	yes	n.o.	no	Yes	dd	9.94, 6.18	Overlapped with GSSG
12	**GSSG (Oxidized glutathione)** 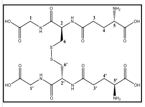	2 -CH	4.75	n.o.	4.75-2.96 (TOCSY)<br>4.75-3.30 (TOCSY)	yes	n.o.	no	no	n.o.	n.m.	Below water supressed resonance in 1D-^{1}H; Observed in TOCSY
		3 -CH_2	2.53	small	2.53-2.15 (TOCSY)	yes	2.53-34.32	yes	yes	m	n.m.	Overlapped with GSH
		4 -CH_2	2.15	tiny	2.15-2.53 (TOCSY)	yes	n.o.	no	yes	dd	8.43,13.55	Overlapped with L-Glutamic Accid, L-Glutamine and L-Methionine
		5 -CH ; 1 -CH_2	3.77	tiny	3.77-2.15 (COSY)<br>3.77-2.54 (TOCSY)	yes	3.77-46.20<br>3.77-56.88	yes	no	n.o.	n.m.	Overlapped with alpha-CH from L-Glutamic Acid, L-Glutamine and D-Glucose
		6 -CH_2-S-S	2.96	tiny	2.96-3.29 (COSY)	yes	n.o.	no	yes	dd	14.22, 6.10	Overlapped with GSH
		6' -CH_2-S-S	3.29	tiny	2.94-3.30 (COSY)	yes	n.o.	no	yes	dd	14.3, 4.50	
13	**Glycine** 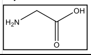	-CH_2	3.54	medium		no	3.54-44.23	yes	yes	s		Overlapped with D-Glucose
14	**Inosinic Ac.** 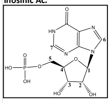	1 -CH	6.13	small	6.13-4.50 (COSY)	yes	6.14-89.26	yes	yes	d	n.m.	Overlapped with AMP+ATP
		2 -CH	4.80	n.o.	4.80-6.13 (COSY)	yes	n.o.	no	no	n.o.	n.m.	Overlapped with AMP+ATP
		3 -CH	4.50	small	4.50-4.36 (COSY)	yes	n.o.	no	yes	m		Overlapped with AMP+ATP
		4 -CH	4.36	small	4.36-4.02 (COSY)	yes	n.o.	no	yes	m		Overlapped with AMP+ATP
		5 -CH_2	4.02	small	4.02-4.50 (TOCSY)	yes	n.o.	no	no	n.o.	n.m.	Overlapped with AMP+ATP
		6 -CH	8.55***	small	n.o.	no	n.o.	no	yes	s		
		7 -CH	8.26	small	n.o.	no	n.o.	no	no	n.o.	n.m.	Overlapped with AMP+ATP

Supplementary Table ST1 (continued)

15	Lactic Ac.	-CH_3	1.31	large	4.10-1.31 (COSY)	yes	1.31-22.80	yes	yes	d	6.92	
	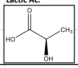	-CH	4.09	large		yes	4.10-71.26	yes	yes	q	6.90	
16	L-Alanine + Phosphothreonine	-CH_3	1.47/1.45	small	1.47-3.77 (COSY)	yes	n.o.	no	yes	yes	7.32 (Ala)	Two partially overlapped doublets at 1.45 and 1.47 ppm (Alanine)
	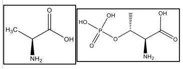				4.82-1.45 (COSY)	yes	1.45-21.4	yes	yes	yes	6.86	Tentative assigment: phosphothreonine
		-CH	3.77	small	3.77-1.47 (COSY)		n.o.	no	no			Overlapped with Glutamic and Glutamine
17	L-Aspartic Ac.	1H-CH_2	2.68	small	2.68-3.88 (COSY)	yes	n.o.	no	yes	part of dd	17.8, 9.75	Overlapped with N-acetyl-L-Aspartic Acid and Carnosine
	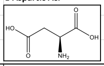	1H-CH_2	2.80	small	2.68-2.80 (COSY)	yes	n.o.	no		part of dd	17.50, 3.70	Partially overlapped with AAMA in treated brain samples
		-CH	3.88	small	3.88-2.80 (TOCSY)	yes	3.88-55.00	yes	no	n.o.	n.m.	Overlapped with GPC, Betaine and Adenosine
18	L-Glutamic Ac.	1 -CH	3.74	medium	3.74-2.12 (COSY)	yes	3.74-57.41	yes	yes	dd	7.06, 5.00	Overlapped with D-Glucose, L-Alanine and L-Glutamine
	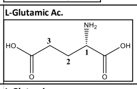	2 -CH_2	2.00-2.12	medium-small	3.74-2.02 (COSY)	yes	2.04-29.61	yes	yes	m	7.22, 16.33	Partially overlapped with L-Glutamine and L-Methionine
		3 -CH_2	2.37-2.32	medium	3.74-2.34 (TOCSY)<br>2.34-2.12 (COSY)	yes	2.34-36.34	yes	yes	m	6.84,7.93,2.57	
19	L-Glutamine	1 -CH	3.76	medium	3.77-2.14 (COSY)<br>3.77-2.44 (TOCSY)	yes	3.76-56.91	yes	yes	t	6.10	Overlapped with D-Glucose, L-Alanine and L-Glutamic Acid
	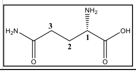	2 -CH_2	2.12-2.16	medium	2.46-2.12 (COSY)	yes	2.13-28.96	yes	no	m	n.m.	Partially overlapped with L-Glutamic Ac.
		3 -CH_2	2.46-2.42	medium		yes	2.44-33.57	yes	yes	m	7.78, 12.21	Partially overlapped with N-acetyl-L-Aspartic Ac.
20	L-Tyrosine	1,4 -CH	7.18	tiny	7.18-6.88 (TOCSY)	yes	n.o.	no	yes	d	8.40	
	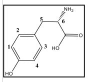	2,3 -CH	6.89	tiny		yes	n.o.	no	yes	d	8.27	
		5 1H-CH_2	3.21*	n.o.	n.o.	no	n.o.	no	no	n.o.	n.m.	Confirmed by spiking
		5 1H-CH_2	3.06*	n.o.	n.o.	no	n.o.	no	no	n.o.	n.m.	
		6 -CH	3.94*	n.o.	n.o.	no	n.o.	no	no	n.o.	n.m.	
21	L-Valine	1 -CH	3.61*	n.o.	n.o.	no	n.o.	no	no	n.o.	n.m.	
	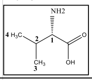	2 -CH	2.28*	n.o.	n.o.	no	n.o.	no	no	n.o.	n.m.	
		3 -CH_3	1.03	tiny	n.o.	no	n.o.	no	yes	d	7.00	Overlapped with broad signal (lipids)
		4 -CH_3	0.98	tiny	n.o.	no	n.o.	no	yes	d	6.94	
22	Methionine sulfoxide	1 -CH_3	2.74	small	n.o.	no	n.o.	no	Yes	s		Confirmed by spiking
	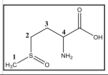	2 -CH_2	3.02**	n.o.	n.o.	no	n.o.	no	no	n.o.	n.m.	Overlapped with Creatine/PC, Carnosine, and 4-AminoButyric
		3 -CH_2	2.31**	n.o.	n.o.	no	n.o.	no	no	n.o.	n.m.	Overlapped with 4-AminoButyric
		4 -CH	3.87**	n.o.	n.o.	no	n.o.	no	no	n.o.	n.m.	Overlapped with D-Glucose and L-Methionine
23	Myo-inositol	2 -CH	3.26	small	3.52-3.26 (TOCSY)	yes	3.26-77.06	yes	no	n.o.	n.m.	Overlapped with Taurine, Betaine and D-Glucose
	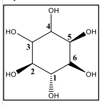	6,4 -CH	3.52	small		yes	3.52-73.94	yes	yes	dd	9.90, 2.90	Overlapped with D-Glucose
		3,1 -CH	3.61	small	3.61-3.26 (COSY)<br>3.61-3.52 (TOCSY)	yes	3.61-75.12	yes	yes	t	9.56	
		5 -CH	4.04	small	4.04-3.52 (COSY)<br>4.04-3.26 (COSY)<br>4.04-3.61 (TOCSY)	yes	4.04-75.10	yes	yes	t	2.87	
24	N-acetyl-L-aspartic Ac.	1 -CH	4.38	medium	4.37-2.68 (COSY)	yes	4.37-55.98	yes	yes	dd	10.10 , 3.80	Overlapping ATP and AMP
	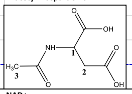	2 -CH_2	2.48	medium	4.37-2.48 (COSY)	yes	2.68-42.39	yes	yes	dd	15.70, 10.10	Overlapping L-Glutamine
		2 -CH_2	2.68	medium	2.68-2.48 (COSY)	yes	2.48-42.39	yes	yes	dd	15.70, 3.90	Overlapping L-Aspartic acid
		3 -CH_3	2.00	large			2.00-24.70	yes	yes	s		
25	NAD+	1 -CH	6.03	tiny	6.03-4.50 (TOCSY)	yes	n.o.	no	yes	d	6.16	
	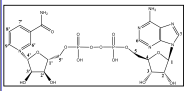	2 -CH	4.37*	n.o.	n.o.	n.o.	n.o.	no	no	n.o.	n.m.	Overlapped with N-acetyl-L-Aspartic Ac
		3 -CH	4.50	n.o.	n.o.	n.o.	n.o.	no	no	n.o.	n.m.	Overlapped with AMP
		4 -CH	4.76*	n.o.	n.o.	n.o.	n.o.	no	no	n.o.	n.m.	
		5 -CH_2	4.25*	n.o.	n.o.	n.o.	n.o.	no	no	n.o.	n.m.	
		6 -CH	8.12*	n.o.	n.o.	n.o.	n.o.	no	no	n.o.	n.m.	
		7 -CH	8.44	small	n.o.	no	n.o.	no	no	n.o.	n.m.	
		1' -CH	6.08	tiny	6.08-4.48 (COSY)	yes	n.o.	no	yes	d	6.09	
		2' -CH	4.48	n.o.		yes	n.o.	no	no	n.o.	n.m.	Overlapped with Carnosine
		3' -CH	4.43	n.o.	6.08-4.43 (TOCSY)	yes	n.o.	no	no	n.o.	n.m.	Overlapped with Carnosine
		4' -CH	4.55*	n.o.	n.o.	n.o.	n.o.	no	no	n.o.	n.m.	
		5' -CH_2	4.36 & 4.24*	n.o.	n.o.	n.o.	n.o.	no	no	n.o.	n.m.	
		6' -CH	9.33	tiny	9.33-8.82 (TOCSY)<br>9.32-9.14 (TOCSY)	yes	n.o.	no	yes	s		
		7' -CH	8.82	tiny	8.82-9.14 (TOCSY)<br>8.82-8.19 (COSY)	yes	n.o.	no	yes	d	7.88	
		8 '-CH	8.19	tiny	8.19-9.14 (COSY)<br>8.19-8.82 (TOCSY)	yes	n.o.	no	no	n.o.	n.m.	
		9 '-CH	9.14	tiny	9.14-9.33 (TOCSY)<br>9.14-8.82 (TOCSY)<br>9.14-8.19 (COSY)	yes	n.o.	no	yes	n.m.	6,22	
26	Taurine	1 -CH_2	3.25	large	3.41-3.25 (COSY)	yes	3.25-50.21	yes	yes	t	6.55	Overlapped with Beta-D-Glucose
	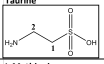	2 -CH_2	3.41				3.41-38.10			t	6.63	
27	L-Methionine	2-CH	3.89	small	n.o.	no	3.89-56.3	yes	yes	dd	4.08, 8.82	Partially overlapped with Aspartic acid, Betaine
	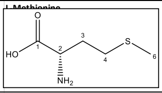	3-CH_2	2.20/2.14	tiny	n.o.	no	n.o.	no	no	m	16.48, 5.19	Partially overlapped with Glutamic Acid and Glutamine
		4-CH_2	2.63	tiny	n.o.	no	n.o.	no	no	t	7.65	Partially overlapped with Glutamic Acid and Glutamine
		6-CH_3	2.12	tiny	n.o.	no	n.o.	no	no	m	16.48, 5.19	Partially overlapped with Glutamic Acid and Glutamine
28	Scyllo-Inositol 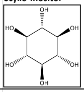	CH	3.33	small			3.33-76.42	yes	yes	s		Single peak, no 1H-1H correlations, compatible with Scyllo-Inositol
29	Phosphorylethanolamine	1-CH_2	3.97	tiny	3.98-3.21 (TOCSY)	yes	3.98-63.06	yes	no	n.o.	n.m.	Overlapped with GPC
	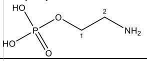	2-CH_2	3.21	tiny	3.21-3.98 (TOCSY)	yes	3.21-43.33	yes	yes	t	4.61	Overlapped with L-Carnosine

*** from Bibliography (PLOS-ONE 2016 e0203379, HMDB or BMRB)**
**** from standard**
*****NMR in Biomed,8, 190-196, 1995**
n.o. not observed
n.m. not measured
peak intensity (tiny, small, medium, large)

Supplementary Table ST2. Cys-containing peptides identified in the proteomic analyses and their proportion in carbamylated derivatives in AA-treated brain samples

Sequence	Cys Position	GeneName	Carbamyl-Cys (Area)	Cys-Propionamide (Area)	Fraction of AA-modification
NSSCVSASSGVSSASR	4	lmna	<1000	15723004	>0.99
TNTEVLPYDTSGIPPGVVICQLPQHR	20	nccrp1	<1000	18259004	>0.99
NEAVAVCTGLDTSEIR	7	akap12b	<1000	20561004	>0.99
SQSLSSCPLHEQPYGEAVSQR	7	si:dkey-34b22.2	<1000	21755004	>0.99
CGFIDEDELAEETGDITQSYGALGAPAK	1	slc17a6b	<1000	27272403	>0.99
VLPEDPCTATPGSSGQSVNVDDNVLAFECTAK	7	map1ab	<1000	59710006	>0.99
IVVESDGGSQASGCTSR	14	pip5k1ca	<1000	30131403	>0.99
HTGPNCPETEGDGLVR	6	hnrnph1l	<1000	35024004	>0.99
SCPSPMQTGGSNDDSK	2	elavl4	<1000	47498003	>0.99
VGVGTCGIADKPMTQYNDTSK	6	smndc1	<1000	51464004	>0.99
LAAVTCNGVDATK	6	sept7b	<1000	69701003	>0.99
CQPSAPESTSEAMK	1	sgsm1b	<1000	88782002	>0.99
SGDAGPSCSSSAAAPPGSAK	8	ppm1g	<1000	90322001	>0.99
AKEHFVDTLDCLQTSPDSR	11	zgc:101040	<1000	92576003	>0.99
NQPLITEPSCEDPSLEKK	10	dbn1	<1000	104675002	>0.99
LDGSHVQLSDIEPCTSASSFSKPDVQDRDHR	14	map1ab	<1000	107817003	>0.99
GHHVAQLDPLGIMDADLDSCVPTDIITSSDK	20	ogdha	<1000	108774002	>0.99
SDHYSYMDMGGDLCDFGSMGNAK	14	rtn1a	<1000	136944001	>0.99
ILAVNNVCLEDVMHEDAVGALK	8	dlg4	<1000	142425000	>0.99
AFGAGADFVMLGGMLAGHCESGGEVIEK	19	gmpr2	<1000	145554001	>0.99
ALLECENFK	5	gstp1;gstp2	<1000	147577000	>0.99
FCINSVALNFKPR	2	msrb2	<1000	178674000	>0.99
ICTTVSDTNIQSR	2	mink1	<1000	226180001	>0.99
EHFVDTLDCLQTSPDSR	9	zgc:101040	<1000	246410001	>0.99
MSELESSKPACVLNSSK	11	vapa	<1000	262110001	>0.99
MGTAMLVPTEEGDLELNCYKPK	18	zgc:153154;zgc:158768	<1000	429478000	>0.99
AFAESFHSVCAQKPWVTK	10	myg1	<1000	535105000	>0.99
CLNPVFNESFPFDVPAHVLR	1	si:ch73-168o14.2;syt7b	11672004	342365000	0.967031684
TCANMAVPPAYADLGK	2	vdac3	53130003	1211360000	0.957983058
QKGSSETCHHCKPK	8	vdac3	882271309	19356387301	0.956406631
AISVGDPLCEGTR	9	aldh9a1a.1;aldh9a1a;aldh9a1a.2	57491003	963670000	0.943700354
EVFDCEGKPR	5	ppp3ca;ppp3cb	206280003	2945850000	0.934558536
GSSETCHHCKPK	6	vdac3	7929436000	1.03161E+11	0.928621799
NQQVEVDCSIGR	8	cops7a	24702004	182688000	0.880891058
LSDDHDVKETCDLK	11	map1ab	19406004	138573003	0.877160869
ETICVSSISETTESHSADIAK	4	akap12b	14799004	105219002	0.876693469
VSTNQEESRGPDCQK	13	akap12b	33736004	197601001	0.854169444
HTSSPCFGEEDPVK	6	scrn3	76191002	434703000	0.8508673
NCQDPQFSTK	2	cpne1;LOC100000238	34145003	188958001	0.846954087
KAHCNEEEK	4	cfl1	65521600	360263000	0.846115618
LAAGLITEVISAATQEVMAVSSCESR	23	akap1b	23410207	100151001	0.810537568
TSVCAQQRPAATVQPAQSPALK	4	syn2b	69727003	296478001	0.809595712
TWLLGMGASIPSPTMNDSSSCK	21	st13	63360201	247264000	0.796022973
QKGGEDEGQPLEVEEGKVPSSTCTPPSSPVR	23	add1	21041004	81414003	0.794631765
SDGDTVSYEYSSLKEEESPTMDLPCLK	25	map1ab	38921003	130630001	0.770446638
CAALDVEPIYTFR	1	zgc:158357	23961004	80294002	0.770169271
HPRPTQHHACVK	10	map2	1420740000	4577750000	0.763150393
STSCSLGQSTFGTK	4	map1aa	260024000	824980000	0.760347427
AFEDEDIIHVEGCVDPVRDIEIIHEELR	13	ola1	164080002	498336000	0.752300667
DLGPADGDMNPRPCTR	14	dpysl5a	888700005	2467939002	0.735241114
MEDIKSEETNVAEQKDEENKICLEK	22	arpp19a	107423002	297751001	0.734871929
TCQPGAGSATYAK	2	map2	391398000	962210000	0.71084834
LTPGHELQPLALIDARPCSR	18	gja1	78878004	190167001	0.706822269
CFLDHFESLEK	1	gstm	2066010000	4919470000	0.704242228
CGFIDEDELAEETGDITLSHAPFGAQGALGAPAK	1	slc17a6a	34010702	79101001	0.699317568
EKDVTGASCQFLESPDIK	9	map1ab	120198003	279181002	0.699037752
ACCPMPFPIIADDKR	2	prdx6	527477806	1170061002	0.689269074
CMPTFHFYK	1	zgc:56493	1967830005	4233550000	0.682678694
AASLQGLVQEDTTTASLCK	18	zgc:153240	33524203	70706001	0.678363836
YVSSGTCGAAGQSLYVANHAY	7	aldocb	2976430000	6149050000	0.673833048
CDTVNAVAQSEK	1	si:ch211-137a8.4	724796000	1495390000	0.673542667
LDLSAEGDQECKK	11	map1ab	172069001	339682001	0.663764213
CDGEQNKPVGATTPEAIK	1	slc12a5a	114346003	205347002	0.642325602
ILCSHLVDYFPEFDGPQNDAQSAR	3	gna11a	51430004	89995000	0.636344334
VGHSISSPTSPCEDMMK	12	phyhipl	161432001	274463000	0.629653929
LDGSHVQLSDIEPCTSASSFSKPDVQDR	14	map1ab	537560000	863270001	0.616256077
EVAEAEEAAAMEQACEGSMTRPK	15	cplx2l	1064790005	1673490000	0.611146412
LQCTNPDAAIENASAEHPITMVFMGYK	3	palm1b	276765000	423430002	0.60473154
ILSFSPNLPCTSEVNITSESEER	10	map1aa	84059001	126179001	0.600172185
NVPESSQHTAISSTMEHEDIVCLK	22	map1ab	603874000	893740000	0.596775938
GNDVSSGCVMSDYVGAGPPK	8	pebp1	623234000	877930000	0.584832836
CSHYDDKDLEFDKQQTSDAVEK	1	map1aa	173788001	233630003	0.573440547
NEGCSQPQDEDIMDIPLDDPAANK	4	nrgna	348504000	461922000	0.56997431
GPCAINDADAK	3	LOC101883393	2300090000	3016790000	0.56739855
HEEFEEGCK	8	glod4	314227000	411454000	0.566990179
TDCGEDAKLEATK	3	ahcyl1	326668001	417823000	0.561219678
SQLEGHSTQNLQQVEDWFMCR	20	nefma	1697350003	2145480000	0.558307289
AAMEQACEGSLTRPK	7	cplx2	1016990000	1283240000	0.557874647
HGESCWNQENR	5	pgam1a	1198930000	1471630000	0.551056707
LGLGPEAGAETHFDYQELCR	19	ptprna	109406002	133854002	0.55025076
KAVPSGCGDDDEEENIMDTVMK	7	cplx2l	495763000	557232000	0.529187698
DLCGASSTLEFEK	3	map1aa	342702000	384898000	0.528996701
AVSEILTETKPTSITLLCSDSQTSGVDGQHETLFSK	18	map1ab	712250000	798700000	0.52860783
ATITCTR	5	camk2a	660200001	734560000	0.526656916
HALPDLTYDYGALEPHICAEIMQLHHSK	18	sod2	213346001	235476001	0.524653426
MADEAVCVGPAPTSK	7	pcca	159445000	175419000	0.523851474
AVPSGCGDDDEEENIMDTVMK	6	cplx2l	51155002	55785002	0.521647652
AIYDTPCILEDSSK	7	psmd12	105164001	113300001	0.518620917
CQAMELLYWR	1	rtn1b	398163000	412481000	0.50883125
QSVGDCKDVYFLK	6	uchl1	146819001	149056001	0.503780313
ASAACTPAGQQSLSR	5	lmnb1	80134002	80417003	0.500881343
SWCPDCVK	3	txndc17	1597470010	1597470000	0.499999998
MKDDEIVCQNSSLCDSPFK	8	zgc:77715	197044010	197044000	0.499999987
VFPLCCVVQNYAWGK	5	mpi	36580012	36580002	0.499999932

EEGLMQSCNQMQACYLFQQDK	8	gad2	30655013	30655003	0.499999918
GLLQGCNSMCAGYLFQPDK	6	gad1a	16619213	16619203	0.49999985
FCAAVTAFAYSQPK	2	nefmb	154360000	149566001	0.492113213
LSPCEWSQPETEPNHFTLLHSLWYTAGALSLQGAGPHPI	4	LOC100151589	3314910000	3012470001	0.476100693
KAAPAEEEMDECEAALASEPK	12	eef1g	142917001	128045002	0.47255704
AVEIPSKPDEESIECQIYEK	15	map1ab	1066780000	947640000	0.470428213
CFLDHFENLEK	1	zgc:173994	280217000	244036001	0.465492807
IHWWENVYGFDMSCIK	14	prmt1	66821000	57300000	0.461646297
VINSELPVLIDFHAQWCGPCK	17	txn2	293995404	247229000	0.456795736
NNPPGGEASGVLCGEPSAPLRR	13	hn1b	297430003	249194002	0.455878263
TYPGLMHSSCPQEMSAVK	10	lypla2	632773000	516288000	0.449312961
DLSCEQLDDILK	4	atp1a1b	460906000	344772001	0.427927783
AEETHTGHSPELDCAR	14	ppp1r14aa	65359003	45057004	0.40806587
KVDFFCPDIVIK	6	si:ch211-288g17.3	1845580000	1271430000	0.40790052
SSDDEPENNKSDFEEGTLPCR	20	map1ab	224504000	154588002	0.407784921
ADGCPSNSSSGGPSEGPVEK	4	nt5dc1	224188000	153051001	0.405713621
KAIPAGCGDDEEEEESIVDTVMK	7	LOC563082	2223160000	1506810005	0.403973759
AIPAGCGDDEEEEESIVDTVMK	6	LOC563082	886230005	597209000	0.402584129
YFGAFGDLSCASAIMGNPK	10	zgc:92880;OTTDARP00000002003	760701005	509749000	0.401234994
SREDCCTKF	5	gpm6bb	223992009	148626001	0.398869612
ECTFLDEEYSQEAKPLKEEK	2	map1aa	429011001	282331001	0.396899101
MLPCEVPCHDK	4	nans	98592011	64778003	0.396510972
ACTHSDSSEGVGQVSHTMEVSC	2	ndrg4	1670236000	1087770004	0.394404509
MDCHIEECSQPQEEEDIMDIPLDDPEANK	3	nrgnb	104071010	66077012	0.388350163
LEELEKEQEKLEEDNCNAPEFVK	16	LOC794256	124145003	78218003	0.386523231
ACHVHTVDQTVHTDEEK	2	enoph1	544600000	341148001	0.385152437
TDQGVTNEAAAAACK	14	glo1	327892000	205260000	0.384993398
EPFDLEAFYSCPHDLPYPDVR	11	si:dkey-21a6.6	486481000	295028000	0.377510688
KEPFDLEAFYSCPHDLPYPDVR	12	si:dkey-21a6.6	44967502	27208302	0.376972621
LTWHSCPEDEQQ	6	cbx3a	272483000	164011000	0.375746287
HNGYDPCNMK	7	ckmt1	1454490000	871893000	0.374784805
TDLVSSKPLVVSTEEEECMVR	18	map1ab	335564001	193692001	0.365970344
ISPCEWEQPEK	4	si:ch211-251b21.1	884563000	507831000	0.364717889
FNTCDFDNMLAEK	4	rpl10	376361000	210267000	0.358433283
VLNEECDQNWYK	6	grb2b;grb2a	201911000	110679001	0.35407083
SSSMLSCEER	7	erc1a	124793001	65351003	0.343692158
YTVCGDSSGATGLSHYLSSYAY	4	aldoca	525118000	272790000	0.34188152
YKEALLGSCAVAADPNAPNVQVTR	9	arhgdia	121811003	61616004	0.335915659
CACASHVATIAEFFQVGLPK	1	dbn1	216635607	108791003	0.334302727
YLICADCEIGPIGWHCLDDKK	4	rabif	184476019	92238002	0.333333315
DTDPECKPVSLQQTYEAK	6	sept10	199613000	98126002	0.329570534
ADALQAGASQFESCAAK	14	zgc:92912	234412001	113975002	0.327150557
ECTFLDEEYSQEAK	2	map1aa	71949001	34770003	0.325808916
EMSGCLEDILVAIVK	5	anxa3b	333762000	158061003	0.321377817
CALEQLHK	1	nefmb	1328840000	626807000	0.320511319
SDPSIVLLLQCDLQK	11	vsnl1a	1664510000	772779000	0.317064985
EQQEKPGGGDVDQLMEECR	18	ccdc136b	424158000	190718000	0.310173108
IVNDDQSFCADIYMEDGLIK	9	dpysl2b	61867001	27816902	0.310166051
VCSFEQPYVAAINK	2	gna11a	253288000	113570001	0.309574824
YFIQGMGYMASSCMTR	13	ndrg2	64947001	28757003	0.306891934
LYSCGEAPNYDR	4	zgc:173994	594617000	255917000	0.300889794
IDDSNQEEVIRHEEITHCK	18	map1b	406963000	174424002	0.300013591
CPEVGEANAMCLATATK	1	pnpo	423251002	179257000	0.29751804
ECMQQQLNEYQELLDVK	2	lmnb2	120665000	46856001	0.279702251
TCLPGFPGAPCAIK	2	hcfc1b;hcfc1a	424882008	163448002	0.27781687
STVLDVEQNACTGDAVEHDIETPHGVLHVTMR	11	ndrg3a	1454318003	452770002	0.23741432
AVVDGSCACGDASPIPTSVDK	7	ppa1b	680276000	190152010	0.218458055
AIQETHDAVADEGQCRPEADKVNFHFITFVNVDGR	15	uchl1	1142817001	312335001	0.214640808
ATTTCTR	5	camk2b1	962690001	258330003	0.211569018
VDVDDAQDVAALCGISCMPTFHFYK	13	txn	908062000	243014000	0.211118988
SVCTLEITDTTGSHQFPAMQR	3	diras1a	777930000	207713000	0.210738574
EAGLCYASIAMATDYDCWKEHEEAVCVDNVLK	5	mtap	124456020	32870004	0.208929223
LLLEHLECLVSR	8	zgc:171298;ppfia3;ppfia2;ppfia4	491302000	124193700	0.201778339
SACKEDIPEEQSNSIACSR	3	LOC557745	5757198000	1445218000	0.200657391
EDIPEEQSNSIACSR	13	LOC557745	177675000	43145004	0.195385396
VSECVGTMEVTVVR	4	slc8a2b	312145000	71559002	0.186495324
QPATTSDAWYGVAHGCTR	16	gad1b	1098240000	250500001	0.185728903
SLLFSSEGETPCCMVDNYRPPQPLK	12	cahz	2157150002	484776010	0.183493409
QVCLWDTCSHLPLWTK	3	eml2	426136002	92688000	0.178650177
SDPSIVLLLQCDMQK	11	hpcal4;vsnl1b	2026105804	433387101	0.176209942
MEEFKDQLPADECNK	13	hspa9	1352350000	287570000	0.175356115
KENQWCEEK	6	skp1	570318000	120020001	0.173856866
DGVGCCVGQYNNK	5	prkar2aa	1179964000	247604000	0.173444628
SVATLQNSSDPHQCSR	14	map7d1b	201277002	40333003	0.166934325
LLQCDPSSASQF	4	hpca	492765000	90602003	0.155308755
AAVPSIQHCLDNCAK	9	pgk1	21925260000	3694260000	0.144197081
CVELQGLLEEEKR	1	slmapb	906350000	150231002	0.142185977
CIKPDFVLVR	1	syn1	7600400000	1205620000	0.136908615
IAECYGDVQGICR	4	atp2b4	758539002	119923002	0.136514729
SREDCCTK	5	gpm6bb	495559803	77271801	0.134894444
ICLTDHFKPLWAR	2	ufc1	103103001	15232004	0.128719342
MKELEESICR	9	serpina1l;serpina1	1942390000	278416001	0.125367097
TDECQPWVLPVVR	4	got1	3286040000	460704000	0.122961163
VDTLCQPEAVSTVAVPAAWCSLNRD	5	necab2	767122001	105840001	0.121242392
AIQETHDAVADEGQCRPEADK	15	uchl1	2311850000	316387000	0.120379935
EALLGSCAVAADPNAPNVQVTR	7	arhgdia	1071200000	133757000	0.111005621
TDHGADIVCK	9	maptb	1725850000	186899001	0.097712246
ACHEQLSVSEITNACFEPANQMVK	2	tuba2	10027541000	1053161000	0.09504461
LGYILTCPSNLGTGLR	7	ckmt2b;ckmt1	7033600000	718060000	0.092633062
GACQQTHAYQDIK	3	gpm6ba	6729200000	670071000	0.090559056
ATACFSDLYSTDLLPALDGDAK	4	gad2	183431001	17874303	0.088792012
DCPLDCK	2	srsf3a;srsf3b	1556800008	139620002	0.082302732
KGDHPWIPKPTAAAAPCK	17	si:ch211-207l3.1	550960001	48956003	0.081604762
EMFGSGTACVVSPVGR	9	bcat1	311499001	26013003	0.077072823
NDLLEYINCHFK	9	uqcrc1	1546460000	104711000	0.063416206
SCCSCCPSGCSK	2	mt2;mt	20143515003	1271260002	0.059363687
SDQECFEENQLASLDPIK	5	pnpo	190918000	12021004	0.059234567
GVDACHLLAPHNADFAFSLYK	5	serpina1l;serpina1	2771710000	171860000	0.058384886
SVNHGFCFNILCVGETGLGK	7	sept6	3378164000	198564000	0.055515544

KPEKDEWGSGVEALECALQLEK	16	fth1a	1333390000	74889001	0.053177674
GYPMEIQQEFPGVPSHLDAAVECPK	23	zgc:152945	665658000	37005004	0.052663942
ADETKDEQFEQCVQNFK	12	amph	2125000000	102826001	0.046155311
QTCASNSGHPTSCCSAAELMSVLFFHTMR	3	tktb	4692422011	224509001	0.045660393
SQMHLDCVQMNDALER	7	zgc:65851	11346669000	476369000	0.04029159
CPEALFQPSFLGMESCGIHETTFNSIMK	1	actb2;actbb;actb1;actba	34780822003	1181198001	0.032845708
DDSSGICWACFK	7	cotl1	2520745000	84625000	0.032480991
ADCVFGQGSPVLTLHR	3	proza	1511200000	49753004	0.03187348
FPELHAHYSLSTTSCGCLTVSGSYYGEK	15	epd	33185240000	1084840000	0.031655602
CIEECIEK	1	glula	43743580001	1412180001	0.03127353
SCWFNEK	2	gstm;gstm3	1618640000	48924003	0.029338606
YDDMAACMK	7	ywhaqa	3421340000	95358002	0.027115778
RPLVLQLINCPTEYAEFLHCK	10	dnm1a;dnm1;dnm1b	5539481000	152321000	0.026761472
IIPESCLVSDELLQYR	6	si:ch211-269m17.1	377363000	10235004	0.026406235
HNNCMASHLTPAVYAK	4	ckmt1	12986195001	326885000	0.024553672
YYDPATCGFDFTGALDDISK	7	got2b	1555650000	38888001	0.024388256
FDAHGDQCTIVCNSFQSGSWCEEHR	8	lgals2a	1414174008	34076004	0.023529089
LMGDACSVNMVIYR	6	stxbp6l	2915317002	63471002	0.02130766
TLNVLINNAGCMMTK	11	flj13639	7303272005	150396000	0.020177448
CRPPYMESMEQVFDQCQQHEVK	1	pacsin1a	1635172004	30532004	0.01832979
GHEFMWNEHLGYVLTCPSNLGTGLR	16	ckbb	29354330000	411012002	0.013808409
YGEVCPAGWKPGSDTIVPDVQK	5	prdx2	5834480000	73880002	0.012504316
MVCDIKPEIHANYR	3	atp6v1g1	17128360001	192584002	0.011118563
DLAGCIHGLANCK	5	idh2	30418758001	293758001	0.009564765
FYTCGEAPNYDK	4	gstm	5563870000	52147003	0.009285407
YDPSSMTIVSNASCTTNCLAPLAK	14	gapdh-2;gapdhs	5039844009	32212003	0.006350877
QLLATASCSQKPSDPVLNALLAPVSK	8	cap1	1241710000	5947004	0.004766538
FCGLCSCPCNK	2	snap25a	42065121000	151307000	0.003584079
EAESCDCLQGFQLTHSLGGGTGSGMGTLLISK	5	zgc:55461;tubb2b;zgc:123292;tubb4b;zgc:65894;zgc:153426	56950064001	110444001	0.001935559
TIQFVDWCPTGFK	8	si:ch73-199e17.1;tuba1c;tuba1b;tuba1a;tuba8l3;tuba7l	51389600000	81471002	0.00158285
KEAESCDCLQGFQLTHSLGGGTGSGMGTLLISK	6	zgc:55461;tubb2b;zgc:123292;tubb4b;zgc:65894;zgc:153426	36843005004	39645004	0.001074896
LIVDGQAISVFQCMK	13	gapdh-2;gapdhs	12165089501	<1000	<0.001
ESENCDCLQGFQLTHSLGGGTGSGMGTLLISK	5	tubb2	6394840015	<1000	<0.001
CIELCCGSVK	1	atp1a1b;atp1a1a.1	2840520010	<1000	<0.001
KSCCSCCPSGCSK	3	mt2;mt	1654075020	<1000	<0.001
VVEDIECLK	7	ndufa10	1644520000	<1000	<0.001
ESESCDCLQGFQLTHSLGGGTGSGMGTLLISK	5	tubb5;zgc:153264	2042120010	<1000	<0.001
SPTDESVGECEVYSK	10	si:dkey-21a6.6	443806000	<1000	<0.001
SCPIIVHCSDGAGR	2	ptprn2	411320005	<1000	<0.001
HTGPNCPDTGGDGLVR	6	hnrnph1	284871002	<1000	<0.001
NVGTGLVGAPACGDVMK	12	iscub;si:ch211-191d15.2	149983000	<1000	<0.001
FSTQDHKPCNPR	9	ppm1bb	94276003	<1000	<0.001
VGYESGDYELLAEGCGVK	15	dctn2	92440001	<1000	<0.001
ASPADSGVCESVSER	9	camlg	15087004	<1000	<0.001
IQCLQQQADDAEDR	3	tpm4a	10870004	<1000	<0.001
MYECFIESIPLLK	4	prkar2aa	9847208	<1000	<0.001
LTTPSYGDLNHLVSATMSGVTTCLR	23	tubb2b	1267370002	<1000	<0.001
LCVPAMNVNDSVTK	2	ahcyl2;ahcyl1	75122009	<1000	<0.001
PLVCGGTSEAK	4	zgc:56530;MGC174082	425507002	<1000	<0.001
NDPPMEAANFNAQVIILNHPGQISQGYAPVLDCHTAHIA	33	eef1a;eef1a1l1	339182010	<1000	<0.001
MVCDVVSR	3	gnao1a	6931960005	<1000	<0.001
AYHEQLSVAEITNSCFEPSNQMVK	15	tuba8l2	207479006	<1000	<0.001
CPVMLVVGDNAPAEEGVVECNSK	1	ndrg4	2379570010	<1000	<0.001
TLESMMACCLSEEAK	8	gna11b	137578012	<1000	<0.001
AFVQLCNENPSVLHLPEMSFFK	6	st13	1499853005	<1000	<0.001
VIEPMACDGLR	7	atp2b2;atp2b4;atp2b3a	1125170005	<1000	<0.001
PFVPHIPFDFYVCEMAFPR	13	ilf2	505191006	<1000	<0.001
SCCSVMSAELSQR	2	si:ch211-251b21.1	4613376006	<1000	<0.001
FFSEGCAPGADPTSNMCK	6	tfa	4467978006	<1000	<0.001
AETEGSPFGGCLEK	11	plp2	97825008	<1000	<0.001
AGKPIICATQMLESMIK	7	pkma	8112121001	<1000	<0.001
QNYELLCMDGSR	7	tfa	1359928001	<1000	<0.001
HYLDQLNHILGVLGSPSQDDLNCIINMK	23	mapk3	1028841004	<1000	<0.001
LTDCVVMR	4	hnrnpa3	533350002	<1000	<0.001
VIPGFMCQGGDFTNHNGTGGK	7	ppiaa	4126802003	<1000	<0.001
AVMFCLSDDKK	5	cfl1	7662104004	<1000	<0.001
AYHEQLSVAEITNACFEPANQMVK	15	tuba1c;tuba1a;tuba1b	28311470000	<1000	<0.001
GSQLEGYCMDLLTELAK	8	LOC100151589	5898220000	<1000	<0.001
PECWDGEHDIETPYGMLHVVIR	3	ndrg4	263700000	<1000	<0.001
VYCMLGDGECSEGSVWEAMAFASHYK	3	tktb	1789460010	<1000	<0.001
ICDPGLTCFEPEALGNLVEGMDFHR	2	camk2b1	10611506008	<1000	<0.001
AEGSDVANAVLDGADCIMLSGETAK	16	pkma	3287337003	<1000	<0.001
GADIMYSGTIDCWR	12	slc25a5	1713363001	<1000	<0.001
VGLIGSCTNSSYEDMGR	7	aco2	2287266000	<1000	<0.001
YSTHLQLAEDCMK	11	stxbp1a	13278362000	<1000	<0.001
MFVDLNPDSDKIIYSHFTCATDTENIR	19	gna11a;gna11b	737514004	<1000	<0.001
SEDFELICPNTPDTTMK	8	tfa	1590989001	<1000	<0.001
LADFGMCK	7	prkcea;prkceb;prkchb;prkcha	1737660005	<1000	<0.001
VVVSAPSPDAPMFVMGVNQDKYDPSSMTIVSNASCTT	35	gapdh-2;gapdhs	7008952002	<1000	<0.001
IPTPLNTSGVQVICMK	14	ap2m1b;ap2m1a	1618781004	<1000	<0.001
SDFEENGSMDNVCLFLNLANDPTIER	13	atp6v1ba;atp6v1b2	293350005	<1000	<0.001
LADQCTGLQGFLVFHSFGGGTGSGFTSLLMER	5	tuba8l2;tuba1c;tuba1a;tuba2	39750210000	<1000	<0.001
EFCQQEVEPMSK	3	otub1b;otub1a	618816005	<1000	<0.001
EYYSEADASHCIQQILEAVLHCHQMGVVHR	11	camk2b1;camk2d1;camk2a	55462580000	<1000	<0.001
CPVMLVVGDQAPYEEAAVECNSK	1	ndrg2	1336240010	<1000	<0.001
NMMAACDPR	6	tubb5;tubb2b;zgc:153264;zgc:123292;tubb2;tubb4b;zgc:65894;zgc:153426	91324670002	<1000	<0.001
KHNLCGETEEEQMR	5	gstm	4656000001	<1000	<0.001
VGDSMVVCGTR	8	si:ch211-113g11.6	5049699002	<1000	<0.001
GCMECCLK	2	gpm6aa	42062910003	<1000	<0.001
AALAGGTTMIVDHVIPEPGCSLLEAFDR	20	dpysl3	3241847002	<1000	<0.001
MMGVPLQCSAILVR	8	gad1b;gad1a	578821003	<1000	<0.001
YYSLTVMTEQEQQQLIDDHFLFDKPVSPLLTCAGMAR	32	ckmt1	6037290005	<1000	<0.001
YMACCLLYR	4	si:ch73-199e17.1;tuba8l2;tuba1c;tuba1b;tuba1a;tuba8l3;tuba2;tuba8l4	1.11688E+11	<1000	<0.001
MCDPSVTAFEPEALGNLVEGLDFHR	2	camk2a	6128090001	<1000	<0.001
LCYVALDFEQEMGTAASSSSLEK	2	actb2;actbb;actb1;actba	8714949000	<1000	<0.001
VFANTEDSACLLGMR	10	pfkpa;pfkpb	1401860005	<1000	<0.001
IMRPTDVPDTGLLCDLLWSDPDKDVQGWGENDR	14	ppp1cb	837082002	<1000	<0.001
TLCSDDTPMVR	3	ppp2r1a;ppp2r1b	1250720005	<1000	<0.001
NADMSEEMQQDAVECATQALEK	15	dynll1	1539257002	<1000	<0.001

EAGVPDGLFNVVQGGAETGSLLCHHPMVAK	23	aldh9a1a.1;aldh9a1a	527945005	<1000	<0.001
DEEIAALVVDNGSGMCK	16	actba;actb1	2327418000	<1000	<0.001
DDEIAALVVDNGSGMCK	16	actb2;actbb	7983510000	<1000	<0.001
GCVVGTK	2	rpl3	1246690001	<1000	<0.001
IDAAMCGPK	6	hpx	2862480005	<1000	<0.001
LPSPPVTPGMECSGVIEAVGEEVTDRK	12	vat1	3691034002	<1000	<0.001
EVMICPDSSLEDAHK	5	park7	2316240001	<1000	<0.001
LPSPPVTPGMECSGVIEAVGEEVTDR	12	vat1	2504106001	<1000	<0.001
KADPQEAINCLNAAIDIYTDMGR	10	napba	4385663000	<1000	<0.001
VSCLGVTDDGMAVATGSWDSFLK	3	gnb1;gnb1b	2833887404	<1000	<0.001
GTLSMANAGPNTNGSQFFICTADTNWLDGK	20	ppiab	1269320000	<1000	<0.001
LQEVPHEGPMCDLLWSDPDDR	11	ppp2cb;zgc:56064	1059794003	<1000	<0.001
QPCHSPQLTSGTMK	3	epd	1.83249E+11	<1000	<0.001
VLDFHHPHQLLEGMEGFNLELSDQPESLEQILVDCR	35	gad1b	1476054001	<1000	<0.001
MDPCECAK	4	mt2;mt	6021466008	<1000	<0.001
LSDQCTGLQGFLVFHSFGGGTGSGFTSLLMER	5	si:ch73-199e17.1;tuba1b	5224022000	<1000	<0.001
GADIMYTGTLDCWR	12	slc25a6	627611005	<1000	<0.001
NALSNSLYCPDIMLGK	9	uqcrc2a	549958004	<1000	<0.001
VMVADEGPYTCSFQAR	11	zgc:110372	603468005	<1000	<0.001
LMLGENDYNIEHTVECK	16	atp1b3b	7545018000	<1000	<0.001
SPAPMDQPCCR	9	sh3gl2	7734116004	<1000	<0.001
MADCGGLPQVVQPGK	4	ndrg3a;ndrg3b	2334229001	<1000	<0.001
QLDNMQFENCGESPLDYK	10	atp1b1b	1150790005	<1000	<0.001
MHESLMLFDSICNNK	12	gnao1b;gnao1a	2757720000	<1000	<0.001
VFIMDNCEELIPEYLNFIR	7	hsp90ab1	4411896002	<1000	<0.001
VCVETVESGVMTK	2	idh2	12003120001	<1000	<0.001
VEDACEMYAR	5	napba	4976496004	<1000	<0.001
YTIVVSATASDAAPLQYLAPYSGCSMGEYFR	24	atp5a1	435390005	<1000	<0.001
LCNDLMNCLQER	2	pacsin1a	5363850006	<1000	<0.001
GGTGAIVEYHGPGVDSISCTGMATICNMGAEIGATTSV	19	aco2	1792460010	<1000	<0.001
YHQYIVECHGNTLLPQFLGMYR	8	pip4k2ab	1586429004	<1000	<0.001
NADMSEDMQQDAVDCATQAMEK	15	dnl2l;dynll2a	3023324002	<1000	<0.001
GAPMLVVCQGK	8	dpysl3	8030674001	<1000	<0.001
LLADCITVCAAMK	5	ba1;ba2	19657694000	<1000	<0.001
CMFVQVLYHACQNYWEAK	1	stxbp6l	1834810004	<1000	<0.001
AVCMLSNTTAIAEAWAR	3	si:ch73-199e17.1;tuba8l2;tuba1c;tuba1b;tuba1a;zgc:123298; tuba8l3;tuba7l;tuba2;tuba8l4;tuba8l	27402530000	<1000	<0.001
YDDMASCMK	7	ywhaqb	7648780000	<1000	<0.001
ECISMHVGQAGAQMGNACWELYCLEHGIQPDGQMPS	2	tuba8l3;tuba8l4	2043921012	<1000	<0.001
SGDAAIVEMIPGKPMCVESFSEYPPLGR	16	eef1a1a	1360663001	<1000	<0.001
VIPQFMCQGGDFTNHNGTGGK	7	ppiab;ppifb	10969287000	<1000	<0.001
ALSDHHVYLEGTLLKPNMVTAGHACSQK	25	aldoaa	5505204003	<1000	<0.001
IVTTCAFMCNAPLHEIHDGIGVMVQEVLK	5	zgc:162944	8707534004	<1000	<0.001
ATTGTATVAEGIPAGWMGLDCGPESSK	21	pgk1	2020245002	<1000	<0.001
MCGLNFADLMAR	2	vat1	5287670000	<1000	<0.001
SDLENCLGTIIEVFHK	6	s100b	1.93388E+11	<1000	<0.001
SSWVMTCAYAPSGNYVACGGLDNICSIYSLK	7	gnb1b;CABZ01087197.1	951752412	<1000	<0.001
SELEIVLCRDPML	8	calb2a	814019002	<1000	<0.001
GLDLAGFLGDMESAPDHSIFVLHACAHNPTGTDPTQDQ	25	got1	8201080004	<1000	<0.001
ADQELMVYSHDNIICGITSVAFSK	15	gnb1;gnb1b	8782520000	<1000	<0.001
AHCPIYLVNVSSMSAGDVLASAK	3	dpysl5a	3911196002	<1000	<0.001
MFEPACLDDFK	6	gstm;zgc:173994	3650895000	<1000	<0.001
VLWYCCGPTVYDASHMGHAR	5	cars	140836006	<1000	<0.001
LVTCSMDDTVR	4	wdr1	2543700005	<1000	<0.001
FQSSAVMALQESSEAYLVGLFEDTNLCAIHAK	27	si:ch211-113a14.27	1.03743E+11	<1000	<0.001
HSMNPFCEIAVEEAVK	7	etfb	1024996001	<1000	<0.001
ITYNQCGDVMR	6	zgc:153867;MLC3SM	1460180005	<1000	<0.001
MSSYAYFVQTCR	11	hmgb1a;hmgb1b	5273034000	<1000	<0.001
IMYNQCGDVMR	6	myl6	1253540005	<1000	<0.001
VEDMPGDCGYAHNDEFITK	8	zgc:175088	2714719004	<1000	<0.001
ECISIHVGQAGVQIGNACWELYCLEHGIQPDGQMPSDK	2	tuba1c;tuba1b;tuba1a;tuba2	28586400000	<1000	<0.001
LFLDEDDKPHNPMVNAGAIVCTSLIK	21	glsb	195392006	<1000	<0.001
ALSDHHVYLEGTLLKPNMVTAGHSCPTK	25	aldocb	47352335000	<1000	<0.001
TVDAYGHIVIPGGIDVNTCLQAPHLGMSPADDFYHGTR	19	dpysl4	2028290005	<1000	<0.001
AMFDHHVYLEGTLLKPNMVTPGHGCPTK	25	aldoca	4696089000	<1000	<0.001
AQNMLLYWGTGSPPCWR	15	tfa;gstR2;gstR3	2366950005	<1000	<0.001
VSYPLCFIFSSPVGCKPEQQMMYAGSK	6	gmfb	1037960008	<1000	<0.001
TCLLNEEGEVPEKYM	2	glulb	212572005	<1000	<0.001
NKEWEVYDFPGGGCGMGMYNTDESITGFAHSCFQYAI	14	idh2	1336158008	<1000	<0.001
HCPQAMICIISNPVNSTIPITSEVMK	2	mdh2	7971870000	<1000	<0.001
KGADIMYSGTIDCWR	13	slc25a5	3474869003	<1000	<0.001
VTLDDSFMENCDEDTLWLDYK	11	pkma	594007003	<1000	<0.001
RVVVSAPSPDAPMFVMGVNQDKYDPSSMTIVSNASCT	36	gapdh-2;gapdhs	3202978008	<1000	<0.001
DKQTPSGFTLDDVIQTGVDNPGHPFIMTVGCVAGDEET	31	ckbb	2160758001	<1000	<0.001
CMFVQVLYHACQNFWEAK	1	stxbp6l	3295434012	<1000	<0.001
MGIYSPMEVSVSTSGQHLHCR	20	ggctb	8675727001	<1000	<0.001
AADIECHLQPTVIDCMR	6	tfa	7484662008	<1000	<0.001
EGSTHNWQHVTDQIGMFCFTGLKPEQVER	18	got2b;got2a	8336634002	<1000	<0.001
HMEAFNDCNLNALVQHGLR	8	psma1	651555003	<1000	<0.001
EKGHEFMWNEHLGYVLTCPSNLGTGLR	18	ckbb	6386893000	<1000	<0.001
MREIVHLQAGQCGNQIGAK	12	zgc:55461;tubb2b;tubb4b;zgc:153426	4767579003	<1000	<0.001
YMGHYYCFHGHQFSK	7	hpx	7651723001	<1000	<0.001
FQSSAVMALQEASEAYLVGLFEDTNLCAIHAK	27	zgc:113984;si:dkey-108k21.24	58975500001	<1000	<0.001
VHCCLYFIAPSGHGLKPLDIEFMK	3	hm:zeh0351;sept7a;sept7b	9389504004	<1000	<0.001
CDVIAQGIIMAVTDLDLK	1	sucla2	1167565004	<1000	<0.001
VFVLVCAFPSVAAIAALTTMPESPR	6	LOC568033	1068796001	<1000	<0.001
MGLVFDDVVGIVEVINCR	17	cap1	2818541000	<1000	<0.001
EIYCHMTCATDTGNIQVVFDAVTDIIIANNLR	4	gnao1a	3759398002	<1000	<0.001
MREILHLQAGQCGNQIGAK	12	zgc:65894	4212198003	<1000	<0.001
IQDTCGIHNLHGMPGILGAIVGAVTAALASR	5	rhbg	1458268000	<1000	<0.001
MNEEYDVIVLGTGLTECILSGIMSVK	17	gdi2	26729504	<1000	<0.001
SVCGFHLGYLDSETELIDQAMTAVMDLYR	3	vat1	919805001	<1000	<0.001
LTTPTYGDLNHLVSATMSGVTTCLR	23	zgc:55461;tubb5;tubb2;tubb4b;zgc:65894;zgc:153426	1.00567E+11	<1000	<0.001

Supplementary Table ST3. Deregulated Genes in AA-treated samples relative to controls[a]

GeneID	Refseq	ZFIN.ID	Symbol	logFC	FDR	Description
GeneID:100307105	XM_017353641.2	ZDB-GENE-090313-413	si:rp71-80o10.4	2.57022249	1.83E-13	si:rp71-80o10.4
GeneID:541529	NM_001014343.2	ZDB-GENE-050327-67	sh2d5	2.24266478	1.51E-12	SH2 domain containing 5%2C
GeneID:555812	NM_131176.1	ZDB-GENE-990708-8	ucp2	2.06926543	5.49E-12	uncoupling protein 2%2C
GeneID:100148115	NM_001328691.1			2.48134696	5.71E-12	cytochrome P450 2F2-like
GeneID:259194	NM_001004494.1	ZDB-GENE-020801-3	prc1a	2.06853151	6.38E-12	protein regulator of cytokinesis 1a%2C
GeneID:30407	NM_131169.3	ZDB-GENE-990415-119	hoxd13a	2.72639239	6.38E-12	homeobox D13a
GeneID:404617	NM_207085.1	ZDB-GENE-040426-2337	zgc:77287	2.54768796	6.38E-12	zgc:77287
GeneID:565869	NM_001128729.1	ZDB-GENE-060526-244	klf9	3.14733452	6.52E-12	Kruppel-like factor 9
GeneID:335916	NM_199987.1	ZDB-GENE-030131-7859	jun	-2.0805601	1.60E-11	jun proto-oncogene
GeneID:100330841	NM_001245962.1	ZDB-GENE-100629-3	sbno2a	2.21088013	6.88E-11	strawberry notch homolog 2a
GeneID:402971	NM_205672.1	ZDB-GENE-040426-1799	zgc:77158	2.07936499	6.88E-11	zgc:77158
GeneID:568080	NM_001077616.1	ZDB-GENE-061103-553	stk35	2.09640904	3.35E-10	serine/threonine kinase 35
GeneID:569618	XM_021476490.1	ZDB-GENE-060526-202	adamts8a	2.23551843	3.97E-10	ADAM metallopeptidase with thrombospondin type 1 motif%2C 8a
GeneID:560548	XM_005172311.4	ZDB-GENE-160728-113	si:ch211-15b10.6	1.71570995	1.45E-09	si:ch211-15b10.6
GeneID:57935	NM_181601.4	ZDB-GENE-000329-3	actb2	-2.0351368	5.66E-09	actin%2C beta 2
GeneID:570332	NM_001030247.1	ZDB-GENE-050913-48	zgc:114130	1.71225014	7.33E-09	zgc:114130%2C
GeneID:337696	NM_194379.1	ZDB-GENE-030131-9642	cbr1l	1.4004805	1.29E-08	carbonyl reductase 1-like
GeneID:57934	NM_131031.1	ZDB-GENE-000329-1	actb1	-2.3611995	1.29E-08	actin%2C beta 1
GeneID:405850	NM_212914.1	ZDB-GENE-040426-2437	foxg1b	2.05279766	2.96E-08	forkhead box G1b
GeneID:386701	NM_198818.1	ZDB-GENE-031110-4	tubb5	-1.6916879	4.05E-08	tubulin%2C beta 5%2C
GeneID:563855	NM_001110126.2	ZDB-GENE-030131-1957	chac1	1.61449199	4.08E-08	ChaC%2C cation transport regulator homolog 1 (E. coli)
GeneID:558079	XM_681250.7	ZDB-GENE-131127-627	si:ch211-195b15.8	-1.6299631	6.27E-08	si:ch211-195b15.8
GeneID:767746	NM_001076715.2	ZDB-GENE-060929-220	zgc:153426	-1.9367299	6.27E-08	zgc:153426
GeneID:30606	NM_131339.1	ZDB-GENE-980526-531	rarga	1.57939571	1.87E-07	retinoic acid receptor gamma a
GeneID:334936	NM_199929.1	ZDB-GENE-030131-6876	trmt2a	1.41284697	2.38E-07	tRNA methyltransferase 2 homolog A
GeneID:791612	NM_001014296.2	ZDB-GENE-050327-6	mat2ab	2.38423017	2.63E-07	methionine adenosyltransferase II%2C alpha b
GeneID:768172	NM_001077315.1	ZDB-GENE-061013-174	zgc:153911	1.75000242	3.96E-07	zgc:153911
GeneID:327020	NM_199761.1			1.45109232	6.05E-07	RNA binding protein S1%2C serine-rich domain
GeneID:402815	NM_001130592.2	ZDB-GENE-040504-1	nr1d2a	2.2811706	1.36E-06	nuclear receptor subfamily 1%2C group D%2C member 2a
GeneID:368885	NM_001002513.2	ZDB-GENE-030616-566	slc1a4	1.46561908	1.54E-06	solute carrier family 1 (glutamate/neutral amino acid transporter)%2C member 4%2C
GeneID:336473	NM_182866.3	ZDB-GENE-030131-8417	glulb	1.46126027	2.04E-06	glutamate-ammonia ligase (glutamine synthase) b
GeneID:560341	NM_001126109.1	ZDB-GENE-030219-51	mych	1.75871489	2.05E-06	myelocytomatosis oncogene homolog
GeneID:403086	NM_001142774.1	ZDB-GENE-050302-11	gpt2l	1.38115339	5.61E-06	glutamic pyruvate transaminase (alanine aminotransferase) 2%2C like
GeneID:321837	NM_182864.2	ZDB-GENE-030131-556	keap1a	1.1885721	5.65E-06	kelch-like ECH-associated protein 1a
GeneID:368367	XM_021476561.1	ZDB-GENE-030804-21	hsd20b2	1.76035983	7.15E-06	hydroxysteroid (20-beta) dehydrogenase 2
GeneID:565617	NM_212606.2	ZDB-GENE-030131-780	cdh15	2.34934087	7.15E-06	cadherin 15%2C type 1%2C M-cadherin (myotubule)
GeneID:553350	XM_003198999.5	ZDB-GENE-071009-2	cgnl1	0.96845083	7.90E-06	cingulin-like 1%2C
GeneID:117508	NM_131856.3	ZDB-GENE-011109-1	klf2a	1.9603123	9.20E-06	Kruppel-like factor 2a
GeneID:58052	NM_131537.3	ZDB-GENE-000823-6	hoxb5b	3.46425404	9.20E-06	homeobox B5b
GeneID:445144	NM_001003538.2	ZDB-GENE-040801-46	chchd4a	1.25349603	9.39E-06	coiled-coil-helix-coiled-coil-helix domain containing 4a
GeneID:101886224	XM_009295733.3			1.62903521	1.14E-05	uncharacterized LOC101886224%2C
GeneID:445287	NM_001003742.1	ZDB-GENE-040808-62	hpdb	1.30528981	1.14E-05	4-hydroxyphenylpyruvate dioxygenase b
GeneID:406311	NM_001291329.1	ZDB-GENE-040426-1978	pik3r3a	1.70198158	1.26E-05	phosphoinositide-3-kinase%2C regulatory subunit 3a (gamma)%2C transcript variant 2
GeneID:641291	NM_001035265.1	ZDB-GENE-051018-1	ahrra	1.76072361	1.77E-05	aryl-hydrocarbon receptor repressor a
GeneID:555584	NM_001204464.1			1.52868977	2.16E-05	proline rich 5a (renal)%2C
GeneID:334334	NM_201483.1	ZDB-GENE-030131-6266	ivns1abpb	1.61215032	2.46E-05	influenza virus NS1A binding protein b
GeneID:794493	NM_001171589.2	ZDB-GENE-120316-1	gdf10b	-1.3860871	2.59E-05	growth differentiation factor 10b
GeneID:541429	NM_001013555.3	ZDB-GENE-050320-133	aoc2	1.72162152	2.76E-05	amine oxidase%2C copper containing 2
GeneID:724009	NM_001045314.1	ZDB-GENE-060616-266	slc25a47a	1.46665307	2.88E-05	solute carrier family 25%2C member 47a%2C
GeneID:798694	XM_017352961.2			1.37561067	3.05E-05	complement component C6-like%2C
GeneID:100003721	XM_005166734.4	ZDB-GENE-041111-225	fam208b	1.29849669	3.74E-05	family with sequence similarity 208%2C member B%2C
GeneID:799527	NM_001144813.1	ZDB-GENE-100922-34	cxcr3.3	1.4829032	4.87E-05	chemokine (C-X-C motif) receptor 3%2C tandem duplicate 3%2C
GeneID:100535045	XM_003201338.5	ZDB-GENE-130206-2	spidr	1.16756722	5.69E-05	scaffolding protein involved in DNA repair
GeneID:317638	NM_173283.3	ZDB-GENE-021231-1	igfbp1a	1.6751733	6.19E-05	insulin-like growth factor binding protein 1a
GeneID:445164	NM_001003558.1	ZDB-GENE-040801-77	tuba8l3	-2.0196875	6.33E-05	tubulin%2C alpha 8 like 3
GeneID:553686	NM_001020661.1	ZDB-GENE-050522-224	tefb	1.77644373	6.33E-05	thyrotrophic embryonic factor b
GeneID:30317	NM_131101.2	ZDB-GENE-980526-70	hoxb5a	3.1269601	7.12E-05	homeobox B5a
GeneID:100003594	XM_001343070.8	ZDB-GENE-140106-172	fam135b	1.44284185	8.09E-05	family with sequence similarity 135%2C member B%2C
GeneID:266987	NM_001014345.2	ZDB-GENE-021030-3	cbsb	0.85671565	8.09E-05	cystathionine-beta-synthase b
GeneID:30314	NM_131098.1	ZDB-GENE-980526-368	apoeb	1.33281463	8.09E-05	apolipoprotein Eb%2C
GeneID:335032	XM_017353005.2	ZDB-GENE-030131-6972	rapgef6	-2.0520356	8.61E-05	Rap guanine nucleotide exchange factor (GEF) 6
GeneID:565449	XM_005169191.4	ZDB-GENE-091118-93	wscd1b	-1.1417475	8.70E-05	WSC domain containing 1b%2C
GeneID:561110	XM_684512.7	ZDB-GENE-140106-100	tbc1d2	1.46677956	8.87E-05	TBC1 domain family%2C member 2
GeneID:100359374	NM_001172402.1	ZDB-GENE-140619-1	bcl2l16	2.17938655	9.36E-05	BCL2-like 16%2C
GeneID:338214	NM_198914.2	ZDB-GENE-030219-114	sult2st1	1.38270563	0.00010204	sulfotransferase family 2%2C cytosolic sulfotransferase 1
GeneID:641421	NM_001037410.2	ZDB-GENE-030131-722	tubb2b	-1.8185413	0.00010782	tubulin%2C beta 2b
GeneID:794083	XM_001332558.6	ZDB-GENE-120910-1	pdk2a	1.72848634	0.00010782	pyruvate dehydrogenase kinase%2C isozyme 2a
GeneID:553567	NM_001020546.2	ZDB-GENE-050522-134	syt5b	-0.7838899	0.00010956	synaptotagmin Vb
GeneID:795469	NM_001302701.1	ZDB-GENE-091204-19	ccl19b	-1.588295	0.00012202	chemokine (C-C motif) ligand 19b
GeneID:567940	XM_691254.6	ZDB-GENE-081231-1	crhr1	1.63840561	0.00013259	corticotropin releasing hormone receptor 1
GeneID:794891	NM_001082906.2	ZDB-GENE-060526-181	ccl19a.1	-2.4781484	0.00013378	chemokine (C-C motif) ligand 19a%2C tandem duplicate 1%2C
GeneID:100334378	XM_005156957.4	ZDB-GENE-090312-39	kcnf1b	-0.8728547	0.00017518	potassium voltage-gated channel%2C subfamily F%2C member 1b
GeneID:336380	XM_002663518.6			1.81729673	0.00017518	si:dkey-242g16.2%2C
GeneID:492363	NM_001007329.2	ZDB-GENE-041114-206	slc7a3a	1.54895535	0.00018628	solute carrier family 7 (cationic amino acid transporter%2C y+ system)%2C member 3a
GeneID:570832	XM_005161237.4	ZDB-GENE-021120-1	c7b	0.91994846	0.00018628	complement component 7b
GeneID:30498	NM_131248.1	ZDB-GENE-980526-320	egr1	-2.1065016	0.00019112	early growth response 1
GeneID:403309	NM_203483.1	ZDB-GENE-040310-2	rtn4rl2b	-1.6781232	0.00019787	reticulon 4 receptor-like 2b
GeneID:100151256	NM_001109832.1	ZDB-GENE-071004-21	cmbl	1.28828793	0.00022417	carboxymethylenebutenolidase homolog (Pseudomonas)
GeneID:101883881	XM_017352945.2			1.54302345	0.00022417	V-set and immunoglobulin domain containing 10 like 2%2C
GeneID:564472	NM_001045053.2	ZDB-GENE-060503-344	si:dkey-228a15.1	1.29583829	0.00023509	si:dkey-228a15.1
GeneID:554127	NM_001024422.1	ZDB-GENE-050522-384	tubb2	-1.7379141	0.00023567	tubulin%2C beta 2A class IIa
GeneID:552924	NM_001020494.1	ZDB-GENE-050508-1	slc2a15b	2.0382425	0.00024151	solute carrier family 2 (facilitated glucose transporter)%2C member 15b
GeneID:563923	NM_001077380.1	ZDB-GENE-061013-487	lrrc8db	1.29654893	0.00024553	leucine rich repeat containing 8 family%2C member Db%2C
GeneID:564054	XM_009301922.3	ZDB-GENE-100208-2	usp2b	1.13843274	0.00024823	ubiquitin specific peptidase 2b%2C
GeneID:794635	XM_001334604.6	ZDB-GENE-100922-98	si:dkey-8k3.2	1.56379961	0.00026577	si:dkey-8k3.2
GeneID:561007	NM_001128704.1	ZDB-GENE-060503-754	pdk4	1.60029854	0.00027403	pyruvate dehydrogenase kinase%2C isozyme 4
GeneID:555795	XM_021477947.1	ZDB-GENE-141222-48	si:ch211-260e23.9	1.30496118	0.0002905	si:ch211-260e23.9
GeneID:100126128	NM_001109854.1	ZDB-GENE-071004-86	zgc:173594	1.44544567	0.0003506	zgc:173594
GeneID:101886560	XM_021478065.1			1.0651215	0.0003506	sodium/potassium/calcium exchanger 3-like%2C
GeneID:266988	NM_001017721.1	ZDB-GENE-021031-5	smoc2	-1.2874478	0.0003506	SPARC related modular calcium binding 2
GeneID:541344	NM_001013471.2	ZDB-GENE-050320-35	prdx1	1.7391048	0.0003506	peroxiredoxin 1
GeneID:563720	XM_005171198.4	ZDB-GENE-030131-1426	cc2d1b	1.27563118	0.0003506	coiled-coil and C2 domain containing 1B%2C
GeneID:100001405	XM_005165414.4			1.2260497	0.00039326	cell surface glycoprotein 1-like%2C
GeneID:431765	NM_001002218.1	ZDB-GENE-040704-63	nfil3-6	1.34125599	0.00039742	nuclear factor%2C interleukin 3 regulated%2C member 6
GeneID:796299	XM_001920001.6	ZDB-GENE-130530-593	ntn5	1.72972201	0.00041567	netrin 5%2C
GeneID:100330171	XM_002662814.4	ZDB-GENE-030131-9661	si:dkey-112a7.4	-0.9464933	0.00041814	si:dkey-112a7.4%2C
GeneID:574009	NM_001025188.1	ZDB-GENE-050626-142	alpi.2	1.43861054	0.00043274	alkaline phosphatase%2C intestinal%2C tandem duplicate 2
GeneID:555481	NM_001099240.1	ZDB-GENE-070615-2	zgc:154142	-1.0422634	0.00045345	zgc:154142
GeneID:445240	NM_001003634.1	ZDB-GENE-040801-155	fnbp1l	-0.8787032	0.00047469	formin binding protein 1-like
GeneID:767805	NM_001076772.1	ZDB-GENE-060929-1274	klf13	1.34692956	0.00048879	Kruppel-like factor 13
GeneID:569963	XM_005162089.4			1.00798808	0.00051231	syntaphilin b%2C

GeneID:393285	NM_200315.1	ZDB-GENE-040426-975	irs2a	1.04434876	0.00052521	insulin receptor substrate 2a%2C
GeneID:792924	XM_001332531.8	ZDB-GENE-030131-1009	wu:fb55g09	-1.1176764	0.00052765	wu:fb55g09
GeneID:393875	NM_200901.1	ZDB-GENE-040426-1274	sox4b	-0.9711449	0.00055123	SRY (sex determining region Y)-box 4b
GeneID:407676	NM_001083814.1	ZDB-GENE-070424-54	ptpro	-1.4503752	0.00055123	protein tyrosine phosphatase%2C receptor type%2C O
GeneID:103910381	XM_017355453.2			1.25220309	0.00057289	clathrin coat assembly protein AP180-like
GeneID:100149745	NM_001079975.2	ZDB-GENE-070108-1	sesn2	1.32772763	0.00059377	sestrin 2
GeneID:100534657	XM_009295435.3			-1.1479595	0.00061101	neuronal PAS domain protein 4b
GeneID:100536567	XM_003197697.5	ZDB-GENE-090313-261	si:dkey-22i16.9	1.57300381	0.00061101	si:dkey-22i16.9
GeneID:334757	NM_212730.1	ZDB-GENE-040426-2831	sfxn2	0.89924935	0.00062945	sideroflexin 2%2C
GeneID:562652	XM_009307060.3			1.0656058	0.00063894	ARFGEF family member 3%2C
GeneID:553716	NM_001020687.1	ZDB-GENE-050522-358	b3galnt2	-1.6302262	0.00066082	beta-1%2C3-N-acetylgalactosaminyltransferase 2
GeneID:795099	NM_001114453.1	ZDB-GENE-080204-90	egr4	-1.8047232	0.00068291	early growth response 4
GeneID:336681	NM_198809.2	ZDB-GENE-030131-8625	tubb4b	-1.5329844	0.00071552	tubulin%2C beta 4B class IVb
GeneID:60634	NM_131636.2	ZDB-GENE-001103-3	fezf2	-0.9925752	0.00072137	FEZ family zinc finger 2%2C
GeneID:114435	NM_131082.1	ZDB-GENE-010608-3	neurod2	-1.0178503	0.00072445	neurogenic differentiation 2
GeneID:436978	NM_001002705.1	ZDB-GENE-040718-460	tmc6b	-1.2375445	0.00073607	transmembrane channel-like 6b
GeneID:797250	NM_001128358.1	ZDB-GENE-080818-4	slc7a5	1.23911899	0.00079799	solute carrier family 7 (amino acid transporter light chain%2C L system)%2C member 5%2C
GeneID:565590	XM_688859.7			1.31368498	0.00079893	golgin A7 family%2C member Bb
GeneID:100002292	XM_001342080.6	ZDB-GENE-091204-463	si:ch73-63e15.2	1.05146021	0.000821	si:ch73-63e15.2%2C
GeneID:58050	NM_131535.1	ZDB-GENE-000823-4	hoxa4a	2.46790135	0.000821	homeobox A4a%2C
GeneID:100151358	XM_005164905.4	ZDB-GENE-041210-216	gsap	0.75388641	0.00098188	gamma-secretase activating protein%2C
GeneID:100034522	NM_001242996.1	ZDB-GENE-041001-133	mthfd1l	1.61600966	0.00099455	methylenetetrahydrofolate dehydrogenase (NADP+ dependent) 1-like%2C
GeneID:100001360	XM_001336357.7	ZDB-GENE-131121-498	si:dkeyp-110a12.4	-1.1646997	0.00109683	si:dkeyp-110a12.4
GeneID:790945	NM_001080090.1	ZDB-GENE-070313-1	oaz2b	1.23798766	0.00109683	ornithine decarboxylase antizyme 2b
GeneID:561939	XM_005160183.4	ZDB-GENE-121214-154	slc25a23a	1.44453838	0.00110852	solute carrier family 25 (mitochondrial carrier%3B phosphate carrier)%2C member 23a%2C
GeneID:573216	NM_001190982.1	ZDB-GENE-090507-4	tuba1a	-1.9682514	0.00110852	tubulin%2C alpha 1a
GeneID:140817	NM_131887.1	ZDB-GENE-020111-4	cebpd	1.6187118	0.00113006	CCAAT/enhancer binding protein (C/EBP)%2C delta
GeneID:436836	NM_001002563.1	ZDB-GENE-040718-301	stmn4l	-1.3730545	0.00113006	stathmin-like 4%2C like%2C
GeneID:449866	NM_001258317.1	ZDB-GENE-041008-126	si:ch211-39i2.2	2.20239574	0.00113392	si:ch211-39i2.2
GeneID:447889	NM_001004628.1	ZDB-GENE-040912-54	pnp5b	1.17699675	0.00117622	purine nucleoside phosphorylase 5b%2C
GeneID:503885	NM_001077246.2	ZDB-GENE-050309-15	fut9d	1.89056274	0.00117622	fucosyltransferase 9d
GeneID:562496	NM_131292.1	ZDB-GENE-980526-192	col2a1a	-1.1147427	0.00117622	collagen%2C type II%2C alpha 1a%2C
GeneID:751757	NM_001198748.1	ZDB-GENE-060825-309	zgc:153317	1.36313308	0.00117622	zgc:153317
GeneID:559364	XM_682698.9	ZDB-GENE-091204-385	shroom1	1.80404936	0.0012233	shroom family member 1
GeneID:445225	NM_001003619.2	ZDB-GENE-040801-138	uevld	1.10951795	0.00122798	UEV and lactate/malate dehyrogenase domains%2C
GeneID:751692	NM_001045405.2	ZDB-GENE-060825-301	rnaseka	0.95198588	0.00122798	ribonuclease%2C RNase K a
GeneID:336153	NM_001302225.1			-0.8335145	0.00131265	glutamate receptor%2C metabotropic 2a%2C
GeneID:30476	NM_131229.1	ZDB-GENE-990712-17	wif1	-1.2083741	0.00134456	wnt inhibitory factor 1
GeneID:393664	NM_200691.1	ZDB-GENE-040426-1646	tuba8l2	-2.0145836	0.00134456	tubulin%2C alpha 8 like 2
GeneID:394045	NM_201070.3	ZDB-GENE-040426-1067	lmcd1	-1.4677176	0.00134456	LIM and cysteine-rich domains 1%2C
GeneID:101886793	XM_005168744.4	ZDB-GENE-120215-149	znf648	2.8239214	0.00139779	zinc finger protein 648%2C
GeneID:445109	NM_001003503.1	ZDB-GENE-040801-246	elac1	-2.0380299	0.00139779	elaC ribonuclease Z 1%2C
GeneID:565315	NM_001245072.2	ZDB-GENE-050420-93	slc6a15	1.09669539	0.00139779	solute carrier family 6 (neutral amino acid transporter)%2C member 15
GeneID:100002736	XM_001338767.7	ZDB-GENE-131121-569	si:dkey-54n8.2	0.97820701	0.00143562	si:dkey-54n8.2
GeneID:405805	NM_212869.1	ZDB-GENE-040426-2609	trib3	0.95647829	0.00143562	tribbles pseudokinase 3
GeneID:436780	NM_001002507.2	ZDB-GENE-040718-212	nrn1a	-1.2964924	0.0014625	neuritin 1a
GeneID:567809	NM_001110474.1	ZDB-GENE-070424-95	slc6a16b	1.20966273	0.00146317	solute carrier family 6%2C member 16b%2C
GeneID:431744	NM_001002197.1	ZDB-GENE-040704-38	sh3bgrl2	1.0543523	0.00148569	SH3 domain binding glutamate-rich protein like 2
GeneID:553575	NM_001020554.1	ZDB-GENE-050522-116	gsr	0.9171698	0.00148569	glutathione reductase
GeneID:373080	NM_194388.2	ZDB-GENE-030822-1	tuba1b	-1.9973831	0.00155636	tubulin%2C alpha 1b
GeneID:566886	XM_005156297.4	ZDB-GENE-030131-4490	epas1a	1.14477562	0.00156557	endothelial PAS domain protein 1a%2C
GeneID:110439046	XM_021473301.1			-3.8147916	0.00158436	extracellular calcium-sensing receptor-like
GeneID:573122	NM_001105126.2	ZDB-GENE-061114-1	tuba1c	-1.6777779	0.00158436	tubulin%2C alpha 1c
GeneID:335231	NM_199942.2	ZDB-GENE-030131-7171	fbp1a	1.20035012	0.00163176	fructose-1%2C6-bisphosphatase 1a
GeneID:360149	NM_182889.1	ZDB-GENE-030723-2	nfe2l2a	1.06725238	0.00163176	nuclear factor%2C erythroid 2-like 2a%2C
GeneID:393212	NM_200242.1	ZDB-GENE-040426-871	eps8a	-1.4176762	0.00163176	epidermal growth factor receptor pathway substrate 8
GeneID:793871	NM_200208.1	ZDB-GENE-040426-936	desi1b	1.15082748	0.00163176	desumoylating isopeptidase 1b
GeneID:405826	NM_212890.1	ZDB-GENE-040426-2449	ift122	-1.8068584	0.00165898	intraflagellar transport 122 homolog (Chlamydomonas)
GeneID:100150148	XM_001922320.7			1.38464994	0.00172847	Ras association (RalGDS/AF-6) domain family (N-terminal) member 9
GeneID:100004557	NM_001128293.1	ZDB-GENE-081104-366	hyal3	1.04861379	0.00178105	hyaluronoglucosaminidase 3%2C
GeneID:101886947	XM_009294189.3	ZDB-GENE-120827-2	creb5a	-1.2096665	0.00178583	cAMP responsive element binding protein 5a%2C
GeneID:100332988	XM_009296014.2	ZDB-GENE-110408-26	si:dkey-19a16.12	1.14536571	0.0017859	si:dkey-19a16.12
GeneID:393221	NM_200251.2			1.25721349	0.0017859	solute carrier family 43 (amino acid system L transporter)%2C member 2a
GeneID:407623	NM_214777.1	ZDB-GENE-040718-143	b3gnt7l	-1.0324224	0.0017859	UDP-GlcNAc:betaGal beta-1%2C3-N-acetylglucosaminyltransferase 7%2C like
GeneID:563435	NM_001128532.1	ZDB-GENE-070912-212	satb2	1.02928419	0.00184123	SATB homeobox 2%2C
GeneID:799099	NM_001044353.1	ZDB-GENE-080722-2	lpin1	1.49504896	0.00185224	lipin 1%2C
GeneID:140613	NM_131873.1	ZDB-GENE-011213-1	her9	-1.2280662	0.00185639	hairy-related 9%2C
GeneID:101883703	XM_021468209.1			2.13721341	0.00192856	cytosolic phospholipase A2 zeta-like
GeneID:556725	XM_001919486.6	ZDB-GENE-100922-12	fam131bb	1.01082063	0.00194653	family with sequence similarity 131%2C member Bb%2C
GeneID:791180	NM_001080662.2	ZDB-GENE-070112-2022	shisa3	-0.8867317	0.00208456	shisa family member 3
GeneID:30449	NM_001309823.1	ZDB-GENE-990415-93	serpinh1b	1.48036471	0.00210926	serpin peptidase inhibitor%2C clade H (heat shock protein 47)%2C member 1b%2C transcript
GeneID:406356	NM_213083.1	ZDB-GENE-040426-2050	tmem41ab	-2.2740171	0.00210926	transmembrane protein 41ab
GeneID:795591	NM_001326595.1	ZDB-GENE-120214-26	si:ch211-114n24.6	-1.8603304	0.00210926	si:ch211-114n24.6
GeneID:114430	NM_131823.1	ZDB-GENE-010716-1	irx1b	-0.9125126	0.00213606	iroquois homeobox 1b
GeneID:793236	XM_001332932.8			0.98112985	0.00214726	flavin-containing monooxygenase FMO GS-OX-like 1
GeneID:100384898	NM_001177341.1			1.73237498	0.00218014	UDP glucuronosyltransferase 2 family%2C polypeptide B3
GeneID:560385	NM_001089385.1	ZDB-GENE-070424-88	pald1b	-0.8152241	0.00221118	phosphatase domain containing%2C paladin 1b%2C
GeneID:334289	NM_001305589.1	ZDB-GENE-030131-6221	clca1	3.28983237	0.0022497	chloride channel accessory 1
GeneID:101886421	XM_017357749.2			1.06054014	0.00227264	family with sequence similarity 155 member A
GeneID:58128	NM_131605.2	ZDB-GENE-000627-1	fabp7a	-1.3371958	0.00235809	fatty acid binding protein 7%2C brain%2C a
GeneID:794778	NM_001122751.1	ZDB-GENE-081218-1	pacs2	0.94113217	0.00235809	phosphofurin acidic cluster sorting protein 2%2C
GeneID:100329607	XM_009298954.3			1.34928877	0.00242805	ATP-sensitive inward rectifier potassium channel 10
GeneID:323538	NM_199643.2	ZDB-GENE-030131-2258	coro2a	-0.7866281	0.00263849	coronin%2C actin binding protein%2C 2A%2C
GeneID:445507	NM_001004009.2	ZDB-GENE-040822-25	asb12b	3.92282036	0.00263849	ankyrin repeat and SOCS box-containing 12b
GeneID:571925	XM_005158604.4	ZDB-GENE-090821-5	stxbp5b	0.97747661	0.00263849	syntaxin binding protein 5b (tomosyn)%2C
GeneID:570907	NM_001030257.1	ZDB-GENE-050913-46	rab27a	1.36270883	0.00280577	RAB27A%2C member RAS oncogene family
GeneID:751650	NM_001045367.1	ZDB-GENE-060825-117	fam169aa	1.67001525	0.00282777	family with sequence similarity 169%2C member Aa
GeneID:100002448	XM_001921257.5	ZDB-GENE-071218-2	mst1ra	1.51029058	0.00286916	macrophage stimulating 1 receptor a%2C
GeneID:556385	NM_001079960.3	ZDB-GENE-061013-368	mxra8b	1.14768723	0.00293783	matrix-remodelling associated 8b
GeneID:568021	NM_001080036.1	ZDB-GENE-031018-3	tp53inp1	0.9974523	0.00293783	tumor protein p53 inducible nuclear protein 1
GeneID:406293	NM_213020.1	ZDB-GENE-040426-1956	tcp11l2	0.94374967	0.00305541	t-complex 11%2C testis-specific-like 2%2C
GeneID:572690	NM_001030000.2	ZDB-GENE-050320-132	dynll2b	-0.8650379	0.00307337	dynein%2C light chain%2C LC8-type 2b
GeneID:100006041	NM_212749.2	ZDB-GENE-040322-2	rhoab	-1.0654642	0.0032133	ras homolog gene family%2C member Ab%2C
GeneID:324215	NM_001001815.1	ZDB-GENE-030131-2935	igf2b	0.99027181	0.00332283	insulin-like growth factor 2b
GeneID:777637	NM_001077744.1	ZDB-GENE-060616-1	otud7b	0.98694599	0.00332283	OTU deubiquitinase 7B%2C
GeneID:101882481	XM_017352551.2			1.26547828	0.00344565	leucine rich repeat containing 66%2C
GeneID:101882691	XM_021475473.1			1.68184367	0.00346658	uncharacterized LOC101882691%2C
GeneID:555192	NM_001039806.2	ZDB-GENE-060607-11	epas1b	0.97515057	0.00348256	endothelial PAS domain protein 1b
GeneID:565541	XM_009296937.3			1.35125731	0.00348256	regulator of complement activation group 2 gene 2%2C
GeneID:797711	XM_001338141.7			-1.0449584	0.00348256	zinc finger protein ZFP69-like
GeneID:100000332	XM_001922608.6	ZDB-GENE-160728-105	si:ch73-335l21.4	1.23254728	0.0034897	si:ch73-335l21.4
GeneID:560583	NM_001098738.1	ZDB-GENE-070615-12	cbln2b	-0.7193021	0.0034897	cerebellin 2b precursor%2C
GeneID:373084	NM_200100.2	ZDB-GENE-030826-15	higd1a	0.92033672	0.00353043	HIG1 hypoxia inducible domain family%2C member 1A
GeneID:30767	NM_131479.1	ZDB-GENE-980526-68	stat3	0.88815825	0.00361078	signal transducer and activator of transcription 3 (acute-phase response factor)

GeneID:100141346	NM_001114911.1	ZDB-GENE-080220-48	itga8	1.07562791	0.00362888	integrin%2C alpha 8
GeneID:334503	NM_001005293.1	ZDB-GENE-030131-6435	ric8b	-0.8267512	0.00362888	RIC8 guanine nucleotide exchange factor B
GeneID:324153	NM_001004665.1	ZDB-GENE-030131-2873	taf13	-1.2996788	0.00389966	TATA-box binding protein associated factor 13
GeneID:436915	NM_001002642.1	ZDB-GENE-040718-389	camk2n2	-0.7665687	0.00394592	calcium/calmodulin-dependent protein kinase II inhibitor 2
GeneID:393993	NM_201018.1	ZDB-GENE-040426-1344	ralaa	-1.311847	0.00400949	v-ral simian leukemia viral oncogene homolog Aa (ras related)
GeneID:100000026	XM_001335943.7	ZDB-GENE-120823-1	aldh3a1	1.241274	0.00424174	aldehyde dehydrogenase 3 family%2C member A1%2C
GeneID:368621	NM_001004512.2	ZDB-GENE-030616-512	si:busm1-57f23.1	-0.9079644	0.00424234	si:busm1-57f23.1
GeneID:30216	NM_131020.2			-2.2143234	0.00426826	ba1 globin
GeneID:406517	NM_213236.2	ZDB-GENE-040426-2345	ilf2	-0.9506392	0.00426826	interleukin enhancer binding factor 2
GeneID:557237	NM_001319136.1	ZDB-GENE-070402-3	tmem237a	-1.0618224	0.00426826	transmembrane protein 237a
GeneID:338204	NM_198208.1	ZDB-GENE-030219-104	tfdp2	-1.0132472	0.00428086	transcription factor Dp-2
GeneID:100320658	XM_002664212.6	ZDB-GENE-080722-19	loxl4	1.25749707	0.00439148	lysyl oxidase-like 4
GeneID:368924	NM_213149.1	ZDB-GENE-030616-630	fkbp5	2.05565146	0.00446648	FK506 binding protein 5%2C
GeneID:550472	NM_001017775.2	ZDB-GENE-050417-297	zgc:110372	-0.9914325	0.00448552	zgc:110372
GeneID:559384	NM_001123246.1	ZDB-GENE-081105-3	prf1.5	-1.1435854	0.00457733	perforin 1.5%2C
GeneID:100004613	NM_001128365.1	ZDB-GENE-070912-622	tmeff2a	-1.2623417	0.00461612	transmembrane protein with EGF-like and two follistatin-like domains 2a
GeneID:100333821	XM_005162629.4	ZDB-GENE-120910-2	tnfrsf11a	1.14455567	0.00468408	tumor necrosis factor receptor superfamily%2C member 11a%2C NFKB activator%2C
GeneID:795332	NM_001326528.1	ZDB-GENE-130114-1	slc25a1b	1.14439456	0.00471012	slc25a1 solute carrier family 25 (mitochondrial carrier%3B citrate transporter)%2C member 1b
GeneID:100537363	XM_009302767.3			1.32204707	0.00473855	potassium voltage-gated channel subfamily KQT member 2%2C
GeneID:110439911	XM_021477648.1			0.89060182	0.00473855	uncharacterized LOC110439911
GeneID:550262	NM_001017599.1			-0.6361185	0.00473896	H3 histone%2C family 3B.1
GeneID:100034409	XM_021468715.1			1.00164474	0.00480335	si:ch211-221n20.8
GeneID:321485	NM_001044395.2	ZDB-GENE-030131-204	taf9	-1.0460776	0.00502168	TAF9 RNA polymerase II%2C TATA box binding protein (TBP)-associated factor
GeneID:569107	XM_017356545.2	ZDB-GENE-101215-2	dlgap4b	0.91558124	0.00502168	discs%2C large (Drosophila) homolog-associated protein 4b%2C
GeneID:406605	NM_213313.1	ZDB-GENE-040426-2556	fam213b	0.95961553	0.00502947	family with sequence similarity 213%2C member B
GeneID:557375	XM_005170784.4	ZDB-GENE-110419-1	csdc2b	1.34178477	0.00523114	cold shock domain containing C2%2C RNA binding b
GeneID:566696	NM_001080030.1	ZDB-GENE-060503-121	si:ch211-216l23.1	-0.6253724	0.00536856	si:ch211-216l23.1%2C
GeneID:393335	NM_200364.1	ZDB-GENE-040426-1339	hsd17b3	1.0807301	0.00539293	hydroxysteroid (17-beta) dehydrogenase 3%2C
GeneID:327510	NM_199818.1	ZDB-GENE-030131-5721	rhoq	-0.681913	0.00547739	ras homolog family member Q
GeneID:100330736	XM_002667571.6			-0.8568304	0.0056849	si:ch73-66d24.2
GeneID:100534922	XM_003200912.5	ZDB-GENE-131127-474	tmem240b	-1.3175465	0.0056849	transmembrane protein 240b%2C
GeneID:335124	NM_199937.1	ZDB-GENE-030131-7064	ppme1	-0.7134868	0.00578334	protein phosphatase methylesterase 1
GeneID:393939	NM_200964.1	ZDB-GENE-040426-728	atf3	1.47967072	0.00578334	activating transcription factor 3
GeneID:101884119	XM_005162859.4	ZDB-GENE-091204-123	si:ch211-56a11.2	-1.2020955	0.00592775	si:ch211-56a11.2%2C
GeneID:791176	NM_001080658.2	ZDB-GENE-070112-1872	camk1db	-0.8124037	0.00592775	calcium/calmodulin-dependent protein kinase 1Db%2C transcript variant 1
GeneID:321681	NM_205541.2	ZDB-GENE-030131-400	drap1	-1.1203766	0.00597031	DR1-associated protein 1 (negative cofactor 2 alpha)
GeneID:378458	XM_009305753.3	ZDB-GENE-030912-2	rbms2a	1.09287551	0.00604403	RNA binding motif%2C single stranded interacting protein 2a%2C
GeneID:678557	NM_001003884.1	ZDB-GENE-040923-1	slc25a24l	1.05957772	0.00604403	solute carrier family 25 (mitochondrial carrier%3B phosphate carrier)%2C member 24%2C like
GeneID:393376	NM_200405.1	ZDB-GENE-040426-1315	hif1al	0.99099565	0.00607029	hypoxia-inducible factor 1%2C alpha subunit%2C like%2C
GeneID:326903	NM_001328200.1	ZDB-GENE-030131-5102	si:dkey-77f5.3	-1.9246954	0.00616211	si:dkey-77f5.3
GeneID:406511	NM_213230.1	ZDB-GENE-040426-2329	sort1a	0.94192641	0.00619212	sortilin 1a%2C
GeneID:792970	XM_005160218.4	ZDB-GENE-121214-275	adgre5b.3	0.95898907	0.00619559	adhesion G protein-coupled receptor E5b%2C duplicate 3%2C
GeneID:415102	NM_001001842.1	ZDB-GENE-040624-3	crabp1b	-1.4562792	0.00622004	cellular retinoic acid binding protein 1b
GeneID:562279	XM_021476376.1			1.07940622	0.00627282	myosin XIX
GeneID:556258	NM_001305571.1	ZDB-GENE-070501-5	tcap	-1.2974078	0.006335	titin-cap (telethonin)
GeneID:100333693	XM_017358494.2	ZDB-GENE-121031-2	lmf1	-1.0081249	0.0063894	lipase maturation factor 1
GeneID:406528	NM_213245.1	ZDB-GENE-040426-2379	tdh	1.18532262	0.00649843	L-threonine dehydrogenase%2C
GeneID:566591	NM_001045109.2	ZDB-GENE-040901-2	furinb	-1.030676	0.00649843	furin (paired basic amino acid cleaving enzyme) b
GeneID:322455	NM_212619.1	ZDB-GENE-030131-1175	cd9a	-1.0875062	0.00653014	CD9 molecule a%2C
GeneID:334230	XM_009306985.3	ZDB-GENE-100212-3	zranb1a	-1.0418725	0.006592	zinc finger%2C RAN-binding domain containing 1a
GeneID:100007015	XM_009292280.3	ZDB-GENE-100922-124	si:dkey-71b5.7	-2.9645141	0.00661647	si:dkey-71b5.7%2C
GeneID:58042	NM_001115090.3	ZDB-GENE-000323-1	tbr1b	-1.5793554	0.00665459	T-box%2C brain%2C 1b
GeneID:101885825	XM_005168283.4	ZDB-GENE-141216-142	si:zfos-1324h11.5	-0.7950396	0.00666995	si:zfos-1324h11.5
GeneID:393600	NM_200627.1	ZDB-GENE-040426-1443	cdkal1	-1.5174362	0.00675666	CDK5 regulatory subunit associated protein 1-like 1
GeneID:436651	NM_001002378.1			1.19328002	0.00675666	zgc:92066
GeneID:564511	NM_001159985.1	ZDB-GENE-080403-10	vdrb	1.16424526	0.00675666	vitamin D receptor b%2C
GeneID:100147850	XM_001922397.7	ZDB-GENE-090313-216	pgap1	0.7865278	0.00680864	post-GPI attachment to proteins 1
GeneID:103910871	XM_021475707.1			-1.2362173	0.00680864	uncharacterized LOC103910871
GeneID:394115	NM_201140.2	ZDB-GENE-040426-1132	ranbp3a	0.86522355	0.00680864	RAN binding protein 3a%2C
GeneID:567442	XM_017358509.2	ZDB-GENE-121214-54	kcnh4b	0.80054516	0.00680864	potassium voltage-gated channel%2C subfamily H (eag-related)%2C member 4b%2C
GeneID:568254	XM_691574.8	ZDB-GENE-090429-1	sstr1a	-0.9932429	0.00680864	somatostatin receptor 1a
GeneID:793663	NM_001128779.1	ZDB-GENE-031019-1	timm8b	-1.166479	0.00680864	translocase of inner mitochondrial membrane 8 homolog B (yeast)
GeneID:799483	NM_001123317.1	ZDB-GENE-081105-110	si:ch211-251j10.3	1.00129947	0.00680948	si:ch211-251j10.3%2C
GeneID:556619	NM_001048063.1	ZDB-GENE-090505-3	chrna4b	-1.0203973	0.00683403	cholinergic receptor%2C nicotinic%2C alpha 4b
GeneID:100048921	XM_002666936.6	ZDB-GENE-070503-1	flnca	-1.5081689	0.00688303	filamin C%2C gamma a (actin binding protein 280)%2C
GeneID:30274	NM_131067.1	ZDB-GENE-990415-267	foxg1a	-2.039651	0.00693671	forkhead box G1a%2C
GeneID:795588	NM_200410.1	ZDB-GENE-040426-1364	slc16a9a	1.4123147	0.00700451	solute carrier family 16%2C member 9a%2C
GeneID:406409	NM_213135.2	ZDB-GENE-040426-2148	cyb5a	1.04938164	0.00702271	cytochrome b5 type A (microsomal)%2C
GeneID:563937	NM_001161341.1	ZDB-GENE-030131-7901	wu:fj39g12	-0.9553997	0.00706987	natriuretic peptide precursor C-like protein%2C
GeneID:100037331	NM_001089486.1	ZDB-GENE-070410-84	coq2	1.08185347	0.00736334	coenzyme Q2 4-hydroxybenzoate polyprenyltransferase
GeneID:415174	NM_001002084.1	ZDB-GENE-040625-61	med6	-0.9824921	0.00736334	mediator complex subunit 6
GeneID:550376	NM_001017681.1	ZDB-GENE-050417-170	cib1	-1.4304797	0.00736334	calcium and integrin binding 1 (calmyrin)
GeneID:100332699	XM_021468241.1			-1.5828886	0.00738091	histone deacetylase 9-B-like%2C
GeneID:393650	NM_001313779.1	ZDB-GENE-040426-1518	irf7	1.18694556	0.00743812	interferon regulatory factor 7%2C transcript variant 2
GeneID:566054	NM_001281990.1			-1.6063871	0.00743812	R-spondin 2
GeneID:751721	NM_001045431.2	ZDB-GENE-030813-3	cort	-0.8735632	0.00748421	somatostatin 3
GeneID:799627	XM_001339926.8	ZDB-GENE-080102-4	ptpn21	1.4394228	0.00748421	protein tyrosine phosphatase%2C non-receptor type 21%2C
GeneID:100073328	NM_001098765.1	ZDB-GENE-070615-25	slc38a3a	0.85382669	0.00763923	solute carrier family 38%2C member 3a
GeneID:558716	NM_001076562.2	ZDB-GENE-060929-998	galnt11	-1.0642845	0.00767122	UDP-N-acetyl-alpha-D-galactosamine:polypeptide N-acetylgalactosaminyltransferase 11 (GalNAc-T11)
GeneID:100002129	NM_001190755.1	ZDB-GENE-070501-4	slc1a5	1.03158602	0.00779459	solute carrier family 1 (neutral amino acid transporter)%2C member 5
GeneID:768183	NM_001077326.1	ZDB-GENE-061013-194	gabra1	0.79296425	0.00827757	gamma-aminobutyric acid (GABA) A receptor%2C alpha 1%2C
GeneID:100002307	NM_001110123.1	ZDB-GENE-080204-2	oscp1a	-0.8273154	0.00842859	organic solute carrier partner 1a%2C
GeneID:101882194	XM_021476391.1	ZDB-GENE-100921-23	si:ch73-72b7.1	0.90411523	0.00842859	si:ch73-72b7.1
GeneID:110437720	XM_017354877.2			-2.2747502	0.00842859	WW domain binding protein 11
GeneID:266958	NM_170762.2	ZDB-GENE-030730-1	neurod4	-1.119676	0.00842859	neuronal differentiation 4
GeneID:555643	NM_001256631.1	ZDB-GENE-030131-6898	hnrnpm	-0.7408839	0.00842859	heterogeneous nuclear ribonucleoprotein M%2C
GeneID:327336	NM_199790.1	ZDB-GENE-030131-5547	rbm25b	-0.9789439	0.00851598	RNA binding motif protein 25b%2C
GeneID:562810	NM_001033918.2	ZDB-GENE-050912-2	pmelb	-1.7767369	0.0086041	premelanosome protein b%2C
GeneID:406363	NM_213090.2	ZDB-GENE-040426-2068	scinlb	-1.4251871	0.00862303	scinderin like b
GeneID:100000660	NM_001098758.1	ZDB-GENE-070615-16	ppwd1	-0.7393886	0.00872484	peptidylprolyl isomerase domain and WD repeat containing 1%2C
GeneID:100151416	NM_001128420.1	ZDB-GENE-070705-7	cdkn1a	1.5458927	0.00872484	cyclin-dependent kinase inhibitor 1A
GeneID:100009620	NM_001081554.1	ZDB-GENE-070207-2	defbl2	1.73188648	0.00885596	defensin%2C beta-like 2
GeneID:321868	NM_212599.1	ZDB-GENE-030131-587	ilf3b	-0.7952627	0.00885596	interleukin enhancer binding factor 3b%2C
GeneID:445404	NM_001003881.3	ZDB-GENE-040930-9	snrpf	-0.787533	0.00889842	small nuclear ribonucleoprotein polypeptide F
GeneID:108179129	XM_017356216.2	ZDB-GENE-131121-284	si:zfos-375h5.1	0.75908833	0.00893949	si:zfos-375h5.1
GeneID:30067	NM_001312685.1	ZDB-GENE-000210-28	chico	0.89267211	0.00894999	chico%2C transcript variant 2
GeneID:405828	NM_212892.1	ZDB-GENE-040426-2533	nup43	-1.2222404	0.00902013	nucleoporin 43
GeneID:100141487	NM_001115104.1	ZDB-GENE-080226-7	si:ch211-66e2.3	1.20722126	0.00909969	si:ch211-66e2.3
GeneID:494534	NM_001009990.1	ZDB-GENE-050116-3	cdc40	-1.0584692	0.00915461	cell division cycle 40 homolog (S. cerevisiae)
GeneID:566998	XM_009303869.3	ZDB-GENE-081105-16	pde4d	0.99036316	0.00915461	phosphodiesterase 4D%2C cAMP-specific%2C
GeneID:406249	NM_212977.2	ZDB-GENE-040426-2902	ddx39ab	-1.5281645	0.00917352	DEAD (Asp-Glu-Ala-Asp) box polypeptide 39Ab
GeneID:448858	NM_001252504.1	ZDB-GENE-040917-1	txnipb	-1.7583923	0.00935487	thioredoxin interacting protein b
GeneID:571753	XM_695376.7	ZDB-GENE-030131-5877	ppp1cc	-0.6200648	0.00936049	protein phosphatase 1%2C catalytic subunit%2C gamma isozyme
GeneID:103911292	XM_009302780.3	ZDB-GENE-121214-273	si:ch211-281p14.2	1.4470041	0.00948545	si:ch211-281p14.2%2C

GeneID:325037	NM_001037370.2	ZDB-GENE-030131-3762	tsr1	-0.9128764	0.00948545	TSR1%2C 20S rRNA accumulation%2C homolog (S. cerevisiae)%2C
GeneID:553715	NM_001020686.1	ZDB-GENE-050522-359	ddx47	-1.7250785	0.00948545	DEAD (Asp-Glu-Ala-Asp) box polypeptide 47
GeneID:555362	XM_021479027.1	ZDB-GENE-030131-6933	tns1b	1.01961377	0.00951008	tensin 1b%2C
GeneID:792613	NM_001128775.1	ZDB-GENE-081022-190	zgc:195173	0.82977885	0.00951885	zgc:195173
GeneID:406482	NM_213201.1	ZDB-GENE-040426-2279	gdpd3a	-1.0860358	0.0095719	glycerophosphodiester phosphodiesterase domain containing 3a
GeneID:557232	NM_001110103.1	ZDB-GENE-080204-11	aspa	1.02232862	0.0095719	aspartoacylase
GeneID:103908625	XM_021478791.1	ZDB-GENE-030131-6722	map4k4	-0.7541704	0.00990606	mitogen-activated protein kinase kinase kinase kinase 4%2C
GeneID:793418	XM_003198822.5	ZDB-GENE-031204-36	apof	0.88295106	0.00990606	apolipoprotein F
GeneID:560478	NM_001030143.1	ZDB-GENE-050913-126	slc25a26	-1.2933305	0.0100508	solute carrier family 25 (S-adenosylmethionine carrier)%2C member 26
GeneID:798390	NM_001082925.1	ZDB-GENE-050208-347	s1pr3a	0.96097999	0.0100508	sphingosine-1-phosphate receptor 3a%2C
GeneID:108182924	XM_017354896.2			1.07429061	0.01006445	interferon-induced very large GTPase 1-like
GeneID:100003595	NM_001100084.1	ZDB-GENE-030131-8256	ak5	-0.9699489	0.0103648	adenylate kinase 5
GeneID:100334870	XM_021478054.1	ZDB-GENE-101230-2	rap1gds1	0.87521729	0.0103648	RAP1%2C GTP-GDP dissociation stimulator 1
GeneID:405782	NM_212856.1	ZDB-GENE-040630-4	ubl3b	-0.7287819	0.0103648	ubiquitin-like 3b%2C
GeneID:447936	NM_001004674.1	ZDB-GENE-040912-122	hmgb2b	-1.3448746	0.0103648	high mobility group box 2b
GeneID:560270	XM_683666.8	ZDB-GENE-070912-475	csrnp1a	1.06660375	0.0103648	cysteine-serine-rich nuclear protein 1a
GeneID:558560	NM_001079972.1	ZDB-GENE-061201-6	lcor	1.05282773	0.01046846	ligand dependent nuclear receptor corepressor%2C
GeneID:777614	NM_001077722.1	ZDB-GENE-061103-202	tpcn2	0.83756039	0.01070662	two pore segment channel 2
GeneID:100000073	XM_001920273.7	ZDB-GENE-120215-73	onecut2	1.60314262	0.01074512	one cut homeobox 2%2C
GeneID:100538248	XM_003199741.5			-1.2868487	0.01074512	uncharacterized LOC100538248%2C
GeneID:325034	NM_001025456.1	ZDB-GENE-030131-3759	pak2b	-0.6033793	0.01074512	p21 protein (Cdc42/Rac)-activated kinase 2b%2C
GeneID:560932	XM_684332.8	ZDB-GENE-050419-112	map3k10	0.88704914	0.0107693	mitogen-activated protein kinase kinase kinase 10
GeneID:555725	NM_001037374.1	ZDB-GENE-051120-48	pcyt1bb	1.22691231	0.01091602	phosphate cytidylyltransferase 1%2C choline%2C beta b
GeneID:619255	NM_001033737.1	ZDB-GENE-050913-105	zgc:114181	-1.5177554	0.01092595	zgc:114181%2C
GeneID:562853	NM_001111170.2	ZDB-GENE-080204-89	kcna2b	0.95037643	0.01099528	potassium voltage-gated channel%2C shaker-related subfamily%2C member 2b%2C
GeneID:564475	NM_001030189.2	ZDB-GENE-041014-339	cyp39a1	0.9956124	0.01099528	cytochrome P450%2C family 39%2C subfamily A%2C polypeptide 1
GeneID:58125	NM_131602.2	ZDB-GENE-000616-2	dedd1	-0.9392502	0.01099528	death effector domain-containing 1
GeneID:796659	XM_001335344.6	ZDB-GENE-090313-196	dnajc22	1.24829227	0.01099528	DnaJ (Hsp40) homolog%2C subfamily C%2C member 22
GeneID:793139	XM_001922038.7	ZDB-GENE-030131-212	evplb	-1.2052187	0.01103222	envoplakin b
GeneID:336541	NM_200018.1	ZDB-GENE-030131-8485	blmh	-0.8957157	0.01132257	bleomycin hydrolase
GeneID:402854	NM_001172565.1	ZDB-GENE-040724-122	ak7a	-0.8785206	0.01132257	adenylate kinase 7a
GeneID:556399	NM_001160126.1	ZDB-GENE-030131-9247	cfp	1.17470054	0.01132257	complement factor properdin%2C
GeneID:562116	NM_001044977.2	ZDB-GENE-060503-240	tinagl1	1.23926744	0.01132257	tubulointerstitial nephritis antigen-like 1
GeneID:567414	NM_001099450.1	ZDB-GENE-060531-19	agpat9l	0.96628613	0.01132257	1-acylglycerol-3-phosphate O-acyltransferase 9%2C like
GeneID:57923	NM_131495.2	ZDB-GENE-000511-2	pycard	1.41873472	0.0113477	PYD and CARD domain containing
GeneID:798433	XM_001336022.8	ZDB-GENE-031002-18	syt9a	-0.6581746	0.01140124	synaptotagmin IXa
GeneID:777619	NM_001077727.1	ZDB-GENE-061103-349	drc3	-1.1231914	0.01149201	dynein regulatory complex subunit 3%2C
GeneID:101883524	XM_005160085.4	ZDB-GENE-141212-331	si:ch73-109d9.3	0.78511854	0.01156324	si:ch73-109d9.3
GeneID:406468	NM_213187.1	ZDB-GENE-040426-2236	dhdds	-0.9445206	0.01173809	dehydrodolichyl diphosphate synthase
GeneID:100006122	NM_001111249.1			0.96745203	0.01180891	uncharacterized LOC100006122
GeneID:30131	NM_130954.2	ZDB-GENE-980526-29	dla	-0.7294265	0.01203843	deltaA
GeneID:751632	NM_001045352.2	ZDB-GENE-060503-675	exosc5	-1.1691494	0.01203843	exosome component 5
GeneID:767797	NM_001076764.1	ZDB-GENE-060929-668	utp18	-1.5166589	0.01203843	UTP18 small subunit (SSU) processome component
GeneID:100310831	XM_003199013.5	ZDB-GENE-091014-1	galnt18b	-0.8509827	0.0120777	UDP-N-acetyl-alpha-D-galactosamine:polypeptide N-acetylgalactosaminyltransferase 18b%2C
GeneID:573354	NM_001020556.1	ZDB-GENE-050522-398	lim2.1	-1.0174611	0.01212234	lens intrinsic membrane protein 2.1
GeneID:100151009	NM_001076621.2	ZDB-GENE-041014-209	mep1b	-2.0626594	0.01221794	meprin A%2C beta
GeneID:192316	NM_173243.2	ZDB-GENE-020419-38	terfa	-1.6509229	0.01221794	telomeric repeat binding factor a
GeneID:562242	XM_021472294.1	ZDB-GENE-050411-103	im:7155962	0.99609554	0.01221794	ribosomal protein S6 kinase b%2C polypeptide 1a%2C
GeneID:570994	NM_001105277.3	ZDB-GENE-070928-1	sf3b2	-1.1304556	0.01221794	splicing factor 3b%2C subunit 2
GeneID:767674	NM_001076613.1	ZDB-GENE-060929-412	fam102ab	0.87409397	0.0122347	family with sequence similarity 102%2C member Ab
GeneID:436707	NM_001002434.1	ZDB-GENE-040718-131	havcr1	1.17770952	0.01231846	hepatitis A virus cellular receptor 1%2C
GeneID:797538	NM_001145766.1	ZDB-GENE-090409-2	htr1ab	-0.8826126	0.01231846	5-hydroxytryptamine (serotonin) receptor 1A b
GeneID:322720	NM_001305471.1	ZDB-GENE-080225-27	itpkcb	1.09245404	0.01244794	inositol-trisphosphate 3-kinase Cb
GeneID:406500	NM_213219.2	ZDB-GENE-040426-2303	akap8l	-1.2106286	0.01250683	A kinase (PRKA) anchor protein 8-like
GeneID:573301	NM_001083855.2	ZDB-GENE-041114-92	nexn	-0.962344	0.01250775	nexilin (F actin binding protein)
GeneID:402941	NM_205644.1	ZDB-GENE-040426-1810	insm1a	-0.742814	0.01252128	insulinoma-associated 1a
GeneID:101884909	XM_021469223.1			0.95600573	0.01255208	calcium/calmodulin-dependent protein kinase kinase 2-like
GeneID:799747	NM_001110040.1	ZDB-GENE-070912-301	kcnq3	0.88072695	0.01294599	potassium voltage-gated channel%2C KQT-like subfamily%2C member 3%2C
GeneID:560986	XM_001921173.6	ZDB-GENE-050107-4	pnpla6	0.81366193	0.01294685	patatin-like phospholipase domain containing 6%2C
GeneID:100002266	XM_017351758.1	ZDB-GENE-081028-31	si:ch211-236p5.3	0.92491651	0.01324075	NACHT%2C LRR and PYD domains-containing protein 3-like
GeneID:100149572	NM_001007303.1	ZDB-GENE-041114-130	arfgap1	-0.8973503	0.01324075	ADP-ribosylation factor GTPase activating protein 1
GeneID:30569	NM_131306.2	ZDB-GENE-990415-49	dlx5a	-0.8597528	0.01324075	distal-less homeobox 5a
GeneID:30608	NM_131341.1	ZDB-GENE-990415-87	gap43	-1.3951356	0.01324075	growth associated protein 43
GeneID:30700	NM_131425.2	ZDB-GENE-990415-71	etv4	-1.0534162	0.01324075	ets variant 4%2C
GeneID:571581	XM_695185.7	ZDB-GENE-131009-1	nrbf2a	1.49224309	0.01326894	nuclear receptor binding factor 2a
GeneID:100147967	NM_001166337.1	ZDB-GENE-100309-1	nlgn4xb	1.0766386	0.01329262	neuroligin 4b%2C
GeneID:100331227	XM_017351221.2	ZDB-GENE-050517-32	abcf3	-1.1507775	0.01334449	ATP-binding cassette%2C sub-family F (GCN20)%2C member 3
GeneID:101885373	XM_021468298.1			0.84761853	0.01367691	potassium/sodium hyperpolarization-activated cyclic nucleotide-gated channel 2-like
GeneID:334628	NM_001005393.1	ZDB-GENE-030131-6560	boc	-0.8134083	0.01367691	BOC cell adhesion associated%2C oncogene regulated
GeneID:553537	XM_017353668.2	ZDB-GENE-110114-3	cyp2aa11	1.26444273	0.0136838	cytochrome P450%2C family 2%2C subfamily AA%2C polypeptide 11
GeneID:108179178	XM_017355799.2			1.41595299	0.01385583	gastrula zinc finger protein XlCGF8.2DB
GeneID:335119	NM_199936.1	ZDB-GENE-030131-7059	serpinb1l3	-2.0412611	0.01385583	serpin peptidase inhibitor%2C clade B (ovalbumin)%2C member 1%2C like 3
GeneID:378965	NM_198145.2	ZDB-GENE-031008-3	fem1c	-1.0559427	0.01385583	fem-1 homolog c (C. elegans)
GeneID:560202	NM_001111081.2	ZDB-GENE-071119-4	ghrb	-7.0192787	0.01385583	growth hormone receptor b
GeneID:795096	XM_001333771.7	ZDB-GENE-131120-86	ifit16	1.35221637	0.01385583	interferon-induced protein with tetratricopeptide repeats 16
GeneID:100141327	XM_002662840.5	ZDB-GENE-080327-32	igdcc4	1.05883008	0.01385789	immunoglobulin superfamily%2C DCC subclass%2C member 4
GeneID:322178	XM_005169617.4	ZDB-GENE-030131-897	si:ch211-168f7.5	-0.6802503	0.01385789	si:ch211-168f7.5
GeneID:393467	NM_001362378.1	ZDB-GENE-040426-1546	hnrnpa1a	-0.597839	0.01385789	heterogeneous nuclear ribonucleoprotein A1a
GeneID:403019	NM_207184.1	ZDB-GENE-040707-1	irx1a	-1.0259578	0.01385789	iroquois homeobox 1a%2C transcript variant 1
GeneID:559430	XM_005174361.4	ZDB-GENE-091013-4	rassf10a	0.92407937	0.01385789	Ras association (RalGDS/AF-6) domain family (N-terminal) member 10a
GeneID:563711	XM_687068.6			1.06613206	0.01385789	bestrophin 4
GeneID:567331	NM_001130605.1	ZDB-GENE-060130-6	sparcl1	-0.9253834	0.01385789	SPARC-like 1
GeneID:792692	NM_001029952.1	ZDB-GENE-050706-128	aldoca	-1.012188	0.01385789	aldolase C%2C fructose-bisphosphate%2C a
GeneID:798703	NM_001200037.1	ZDB-GENE-120409-2	gemin7	-1.3404888	0.01385789	gem (nuclear organelle) associated protein 7
GeneID:572909	NM_205678.1	ZDB-GENE-040426-1875	myoz1a	-1.3715833	0.01389224	myozenin 1a
GeneID:436611	NM_001002339.1	ZDB-GENE-040718-28	arl5a	-0.9696189	0.0139326	ADP-ribosylation factor-like 5A
GeneID:557526	XM_005165499.4	ZDB-GENE-030131-5345	traf2b	0.87428373	0.01409674	Tnf receptor-associated factor 2b%2C
GeneID:406458	NM_213179.1	ZDB-GENE-040426-2217	slc20a1a	1.23565459	0.01414902	solute carrier family 20%2C member 1a%2C
GeneID:406443	NM_213164.2	ZDB-GENE-040426-2191	ywhah	-0.6094808	0.01432543	tyrosine 3-monooxygenase/tryptophan 5-monooxygenase activation protein%2C eta polypeptide
GeneID:791218	NM_001080700.1	ZDB-GENE-070112-2282	aifm4	1.71376119	0.01432543	apoptosis-inducing factor%2C mitochondrion-associated%2C 4%2C
GeneID:555589	NM_001098732.1	ZDB-GENE-080214-5	hspb15	1.03245647	0.01449286	heat shock protein%2C alpha-crystallin-related%2C b15
GeneID:566073	NM_001128730.1	ZDB-GENE-050419-160	si:ch211-203b8.6	-0.7879759	0.01455613	si:ch211-203b8.6
GeneID:558612	NM_001245085.1	ZDB-GENE-060503-135	hepacam2	-1.1986651	0.01460647	HEPACAM family member 2
GeneID:494450	NM_001009557.1	ZDB-GENE-050105-2	slc6a5	1.50818069	0.01472739	solute carrier family 6 (neurotransmitter transporter)%2C member 5%2C
GeneID:541335	NM_001013463.1			1.18754383	0.01472739	zgc:114046
GeneID:560869	XM_002666100.3	ZDB-GENE-030131-8760	elf3	-1.8401817	0.01472739	E74-like factor 3 (ets domain transcription factor%2C epithelial-specific)
GeneID:566247	NM_001145593.2	ZDB-GENE-090313-134	si:ch211-89o9.4	-1.574812	0.01472739	si:ch211-89o9.4
GeneID:567704	NM_001077448.2	ZDB-GENE-061019-3	cdh7a	-0.5592605	0.01472739	cadherin 7%2C type 2%2C
GeneID:103908925	XM_017351488.2			1.18659342	0.01480869	uncharacterized LOC103908925
GeneID:497637	NM_001012377.1	ZDB-GENE-050221-2	xirp1	1.28051518	0.01480869	xin actin binding repeat containing 1%2C
GeneID:100535713	XM_021476960.1			-1.4236482	0.01487607	transcription activator BRG1-like
GeneID:326908	NM_001089334.1	ZDB-GENE-030131-5107	hmg20a	-0.7387685	0.01487607	high mobility group 20A%2C
GeneID:100330048	XM_002660710.5	ZDB-GENE-121022-1	brinp3b	-0.8652697	0.01505235	bone morphogenetic protein/retinoic acid inducible neural-specific 3b%2C
GeneID:406770	NM_213461.1	ZDB-GENE-040426-2816	arhgdia	-0.7468871	0.01505463	Rho GDP dissociation inhibitor (GDI) alpha

GeneID:790930	NM_001080075.1	ZDB-GENE-030131-7972	nell2a	-1.0707633	0.01508917	neural EGFL like 2a
GeneID:567953	NM_001128741.1	ZDB-GENE-081022-175	zgc:195023	-0.8113046	0.01517569	zgc:195023
GeneID:322509	NM_212620.2	ZDB-GENE-030131-1229	acta2	-1.2853218	0.01519814	actin%2C alpha 2%2C smooth muscle%2C aorta
GeneID:30344	NM_131121.2	ZDB-GENE-990415-109	hoxb9a	1.10739824	0.01530784	homeobox B9a
GeneID:553383	NM_001098488.1	ZDB-GENE-051113-60	sptan1	-0.9937275	0.01530784	spectrin alpha 2%2C
GeneID:558154	NM_001044855.2	ZDB-GENE-030131-4605	vegfab	-1.1874559	0.01530784	vascular endothelial growth factor Ab%2C
GeneID:561618	XM_005160996.4	ZDB-GENE-091030-1	slc4a4b	1.18361808	0.01530784	solute carrier family 4 (sodium bicarbonate cotransporter)%2C member 4b%2C
GeneID:571158	XM_005172915.4	ZDB-GENE-030131-1704	zbtb40	0.60712305	0.01530784	zinc finger and BTB domain containing 40%2C
GeneID:572518	NM_001317170.1	ZDB-GENE-110411-148	oat	1.03063132	0.01530784	ornithine aminotransferase
GeneID:792024	NM_001168287.1	ZDB-GENE-100921-8	si:ch73-199e17.1	-0.9441033	0.01536393	si:ch73-199e17.1%2C
GeneID:324835	NM_001015043.2	ZDB-GENE-030131-3556	fbxl14b	-1.2855411	0.01544386	F-box and leucine-rich repeat protein 14b
GeneID:393726	NM_200753.1	ZDB-GENE-040426-1719	tbcela	0.78321536	0.01544386	tubulin folding cofactor E-like a
GeneID:493612	NM_001007772.1	ZDB-GENE-041121-7	zgc:101810	-1.8479381	0.01544386	zgc:101810
GeneID:449779	NM_001005952.1	ZDB-GENE-041010-24	mettl1	-1.2458489	0.01551811	methyltransferase like 1%2C
GeneID:606559	NM_001308556.1	ZDB-GENE-050809-3	rnf121	-0.9976415	0.01551811	ring finger protein 121
GeneID:30507	NM_131257.2	ZDB-GENE-980526-79	hbaa1	-2.1346857	0.01562857	hemoglobin%2C alpha adult 1
GeneID:394060	NM_201085.2	ZDB-GENE-040426-1042	hmgcs1	-0.9176582	0.01562857	3-hydroxy-3-methylglutaryl-CoA synthase 1 (soluble)%2C
GeneID:550474	NM_001017777.1	ZDB-GENE-050417-299	babam2	-0.6262705	0.01562857	BRISC and BRCA1 A complex member 2%2C
GeneID:570112	XM_005166490.3	ZDB-GENE-121031-1	serinc4	-0.8871425	0.01562857	serine incorporator 4%2C
GeneID:402966	NM_205667.1	ZDB-GENE-040426-1819	snrpb	-1.119793	0.01580669	small nuclear ribonucleoprotein polypeptides B and B1
GeneID:100149543	NM_002665259.6	ZDB-GENE-090728-1	col12a1a	-0.9378717	0.01587099	collagen%2C type XII%2C alpha 1a%2C
GeneID:100007686	XM_003198028.4			1.04235197	0.01589036	phosphomannomutase 1-like
GeneID:373102	NM_194394.2	ZDB-GENE-030825-1	mcl1b	0.86510002	0.01589036	MCL1%2C BCL2 family apoptosis regulator b
GeneID:555448	NM_001162443.1	ZDB-GENE-031202-1	snx8a	0.80114013	0.01589036	sorting nexin 8a
GeneID:64608	NM_201287.1	ZDB-GENE-001229-2	alas1	-1.3651202	0.01597499	aminolevulinate%2C delta-%2C synthase 1
GeneID:323285	NM_178131.3	ZDB-GENE-030131-2005	scinla	-0.9513287	0.01599029	scinderin like a
GeneID:571005	XM_002667083.3	ZDB-GENE-061220-6	ncdn	-1.0957306	0.01599029	neurochondrin
GeneID:793098	XM_002666683.5	ZDB-GENE-091113-41	yme1l1a	0.67387439	0.01599029	YME1-like 1a%2C
GeneID:327175	XM_001920867.8	ZDB-GENE-030131-5386	larp1	-1.2031007	0.01600476	La ribonucleoprotein domain family%2C member 1%2C
GeneID:569085	XM_005168735.4	ZDB-GENE-110411-262	slc19a2	1.05389687	0.01602708	solute carrier family 19 (thiamine transporter)%2C member 2
GeneID:559896	NM_001079980.1	ZDB-GENE-061103-391	cd99	1.04060577	0.01619277	CD99 molecule%2C
GeneID:564854	NM_001143904.1	ZDB-GENE-071120-7	irf3	1.36180915	0.01621811	interferon regulatory factor 3%2C
GeneID:550449	NM_001017753.1	ZDB-GENE-050417-271	asb5b	0.97774582	0.01635722	ankyrin repeat and SOCS box containing 5b%2C
GeneID:552944	NM_001082800.2	ZDB-GENE-050506-25	lrrc30a	-2.0819721	0.01658876	leucine rich repeat containing 30a
GeneID:559792	XM_683164.8	ZDB-GENE-091118-114	si:ch211-183d21.3	0.7808371	0.01658876	si:ch211-183d21.3
GeneID:570360	XM_003200421.5			0.90666956	0.01658876	protein phosphatase 6%2C regulatory subunit 2b
GeneID:692252	NM_001045235.1	ZDB-GENE-060512-34	pcgf5b	0.85725196	0.01671502	polycomb group ring finger 5b%2C
GeneID:337413	NM_001130590.1	ZDB-GENE-030131-9359	stat6	0.82212883	0.01672323	signal transducer and activator of transcription 6%2C interleukin-4 induced
GeneID:100145909	NM_001309472.1	ZDB-GENE-071005-2	srsf3b	-0.6586081	0.01700675	serine/arginine-rich splicing factor 3b%2C transcript variant 3
GeneID:447866	NM_001004605.1	ZDB-GENE-040912-182	arrdc3b	0.9555731	0.01715714	arrestin domain containing 3b
GeneID:101884099	XM_005158192.4	ZDB-GENE-141212-231	si:ch1073-90m23.1	-1.1585301	0.01764413	si:ch1073-90m23.1
GeneID:347743	NM_178133.3	ZDB-GENE-030411-2	selenoh	-1.3344215	0.01764413	selenoprotein H
GeneID:558193	NM_001110105.2	ZDB-GENE-030131-494	ash2l	-0.5941066	0.01766446	ash2 (absent%2C small%2C or homeotic)-like (Drosophila)%2C
GeneID:565173	XM_688458.8	ZDB-GENE-130530-693	rps6ka5	0.85745792	0.01766446	ribosomal protein S6 kinase%2C polypeptide 5
GeneID:564805	XM_005162826.4	ZDB-GENE-030131-9510	arhgap29a	0.72754571	0.01771443	Rho GTPase activating protein 29a%2C
GeneID:450067	NM_001006087.1	ZDB-GENE-041010-190	ormdl3	0.95109024	0.01783271	ORMDL sphingolipid biosynthesis regulator 3
GeneID:565795	NM_001045086.1	ZDB-GENE-050208-574	nab1a	-0.6688037	0.01783271	NGFI-A binding protein 1a (EGR1 binding protein 1)%2C
GeneID:559897	NM_001079981.1	ZDB-GENE-061020-1	isoc1	0.76884518	0.01792642	isochorismatase domain containing 1
GeneID:569303	XM_021469961.1			-1.7742944	0.01816014	si:dkey-98j1.5%2C
GeneID:335888	NM_199983.1	ZDB-GENE-030131-7831	mylipa	0.73045079	0.01824303	myosin regulatory light chain interacting protein a
GeneID:569546	XM_692941.7	ZDB-GENE-060810-22	ehd4	0.85840662	0.01824303	EH-domain containing 4
GeneID:557983	NM_001044848.2	ZDB-GENE-060616-136	arhgef1b	0.66125329	0.01825568	Rho guanine nucleotide exchange factor (GEF) 1b%2C
GeneID:100148368	NM_001145706.1	ZDB-GENE-080424-3	mmrn2a	0.84698975	0.01833694	multimerin 2a%2C
GeneID:100170782	NM_001130621.1	ZDB-GENE-060810-74	chst6	0.86573461	0.01833694	zgc:194879%2C
GeneID:100535807	XM_017357546.2	ZDB-GENE-140422-2	prkg1l	-0.6323254	0.01833694	protein kinase%2C cGMP-dependent%2C type I%2C like%2C
GeneID:335409	NM_199950.1	ZDB-GENE-030131-7349	socs3a	1.13421598	0.01833694	suppressor of cytokine signaling 3a
GeneID:555432	XM_005171403.4	ZDB-GENE-030131-7031	crema	1.13532518	0.01833694	cAMP responsive element modulator a%2C
GeneID:751758	NM_001045467.1	ZDB-GENE-070912-1	tagln	-2.2358994	0.01833694	transgelin%2C
GeneID:796572	NM_001309798.1	ZDB-GENE-140909-1	sco1	-1.2442738	0.01833694	SCO1 cytochrome c oxidase assembly protein
GeneID:565458	NM_001045081.2	ZDB-GENE-030131-6225	fan1	0.84866985	0.01844633	FANCD2/FANCI-associated nuclease 1
GeneID:447874	NM_001004613.1	ZDB-GENE-040912-38	glod4	-0.6569751	0.01847305	glyoxalase domain containing 4
GeneID:335519	NM_001328424.1	ZDB-GENE-030131-7459	ppiab	-1.3642354	0.01855393	peptidylprolyl isomerase Ab (cyclophilin A)
GeneID:445143	NM_001003537.2	ZDB-GENE-040801-45	sfxn1	0.95098258	0.01855393	sideroflexin 1%2C
GeneID:791169	NM_001080651.2	ZDB-GENE-070112-1542	creb3l2	0.79037291	0.01855393	cAMP responsive element binding protein 3-like 2%2C
GeneID:449542	NM_001005584.1	ZDB-GENE-040930-2	chchd6a	-0.919945	0.01856525	coiled-coil-helix-coiled-coil-helix domain containing 6a%2C
GeneID:548607	NM_001015066.2	ZDB-GENE-050419-21	b3gat1a	0.85763396	0.01856525	beta-1%2C3-glucuronyltransferase 1 (glucuronosyltransferase P) a%2C
GeneID:100333125	XM_005158535.4	ZDB-GENE-100405-4	wdpcp	-1.1495443	0.01865665	WD repeat containing planar cell polarity effector
GeneID:324490	NM_199683.1	ZDB-GENE-030826-26	eif4ebp3l	1.00298544	0.01885137	eukaryotic translation initiation factor 4E binding protein 3%2C like
GeneID:557383	XM_005155890.4	ZDB-GENE-120717-1	bicd2	1.0044601	0.01892194	bicaudal D homolog 2 (Drosophila)%2C
GeneID:323071	NM_198357.3	ZDB-GENE-030131-1791	tcerg1a	-0.9579215	0.01907464	transcription elongation regulator 1a (CA150)
GeneID:436757	NM_001002484.1	ZDB-GENE-040718-187	tmem147	-0.6459079	0.01907464	transmembrane protein 147
GeneID:566647	NM_001030210.2	ZDB-GENE-050303-1	cx32.2	1.34287735	0.01907464	connexin 32.2
GeneID:100302471	NM_001291933.1	ZDB-GENE-090312-190	si:dkey-188i13.7	1.22443073	0.0191226	si:dkey-188i13.7
GeneID:30147	NM_130962.1	ZDB-GENE-980526-112	isl1	-0.9678974	0.01924355	ISL LIM homeobox 1%2C
GeneID:566599	XM_689875.8			0.80515283	0.01926729	si:ch211-117m20.4
GeneID:664747	NM_001039977.2	ZDB-GENE-050913-77	dcbld1	1.04839604	0.01933348	discoidin%2C CUB and LCCL domain containing 1%2C
GeneID:394014	NM_201039.1	ZDB-GENE-040426-804	wdr1	-1.3369474	0.01940827	WD repeat domain 1
GeneID:445026	NM_001003421.1	ZDB-GENE-040801-6	vps26c	-1.4221564	0.01965785	Down syndrome critical region 3
GeneID:336187	XM_003201200.5	ZDB-GENE-030131-8131	klhl17	0.721462	0.01984139	kelch-like family member 17%2C
GeneID:101883345	XM_021478389.1			-1.6628793	0.01984906	si:dkey-21p1.3%2C
GeneID:393882	NM_001320515.1	ZDB-GENE-040426-836	tubg1	-0.6382565	0.01986267	tubulin%2C gamma 1%2C transcript variant 1
GeneID:100333416	XM_002661093.6	ZDB-GENE-141216-161	si:dkey-29p9.3	-0.7735307	0.01994043	si:dkey-29p9.3
GeneID:368709	NM_001007209.1	ZDB-GENE-030616-108	si:busm1-105l16.2	1.04376959	0.01994043	si:busm1-105l16.2%2C
GeneID:562300	NM_001111166.1	ZDB-GENE-030131-6336	nkx2.4a	-0.729679	0.01994043	NK2 homeobox 4a%2C transcript variant 1
GeneID:100332392	XM_002662169.6	ZDB-GENE-100921-13	tbcelb	1.01293005	0.01998438	tubulin folding cofactor E-like b
GeneID:335110	NM_001042745.1	ZDB-GENE-030131-7050	dync1h1	-1.1633903	0.01998438	dynein%2C cytoplasmic 1%2C heavy chain 1
GeneID:394053	NM_201078.1	ZDB-GENE-040426-915	eif4a3	-1.0563714	0.01998438	eukaryotic translation initiation factor 4A3
GeneID:503713	NM_001013307.1	ZDB-GENE-050306-8	ppm1k	0.77755255	0.01998438	protein phosphatase%2C Mg2+/Mn2+ dependent%2C 1K%2C
GeneID:571123	NM_001030258.1			-0.60693	0.01998438	Gpr7%2C
GeneID:793956	XM_009306703.3	ZDB-GENE-130531-36	mkl2a	0.92351813	0.01998438	MKL/myocardin-like 2a%2C
GeneID:560420	NM_001079984.1	ZDB-GENE-070206-7	nrxn2b	-0.7454491	0.0200066	neurexin 2b
GeneID:794185	XM_001334062.7	ZDB-GENE-091204-378	sftpba	0.72564765	0.02021687	surfactant protein Ba
GeneID:564859	XM_021466956.1	ZDB-GENE-030131-4831	stk40	0.60536866	0.02022027	serine/threonine kinase 40%2C
GeneID:565338	NM_001077567.1	ZDB-GENE-061013-268	eif2ak1	0.60468403	0.02022027	eukaryotic translation initiation factor 2-alpha kinase 1
GeneID:322129	NM_212610.1	ZDB-GENE-030131-848	rbb4l	-0.7323567	0.02022722	retinoblastoma binding protein 4%2C like%2C
GeneID:100006301	NM_001109739.1	ZDB-GENE-060526-247	si:dkey-204l11.1	1.32818383	0.02026474	si:dkey-204l11.1%2C
GeneID:100333928	XM_009302790.2	ZDB-GENE-140106-68	alpk1	1.24701316	0.02033567	alpha-kinase 1
GeneID:101885783	XM_005172169.4			0.94500098	0.02033567	phosphofurin acidic cluster sorting protein 1-like
GeneID:336888	NM_001044755.1	ZDB-GENE-030131-8832	susd6	0.79785054	0.02033567	sushi domain containing 6%2C
GeneID:393930	NM_200955.1	ZDB-GENE-040426-1411	bhlhe22	-0.717015	0.02033567	basic helix-loop-helix family%2C member e22
GeneID:565492	NM_001030197.2	ZDB-GENE-050913-90	aplnrb	-1.1097529	0.02033567	apelin receptor b
GeneID:393173	NM_200204.2	ZDB-GENE-040426-928	rpap3	-0.7558664	0.02049777	RNA polymerase II associated protein 3
GeneID:564220	XM_017356704.2			0.84275105	0.02050667	novel protein similar to human membrane-associated guanylate kinase-related (MAGI-3) (MAGI-3)%2C
GeneID:259303	NM_152884.2	ZDB-GENE-020812-1	caspb	1.12937834	0.02052212	caspase b

GeneID:335008	XM_003201387.5	ZDB-GENE-030131-6948	pla2g15	0.85308705	0.02052212	phospholipase A2%2C group XV
GeneID:405857	NM_212921.2	ZDB-GENE-040426-2304	msrb2	0.86038012	0.02052212	methionine sulfoxide reductase B2
GeneID:553336	NM_001171028.1	ZDB-GENE-070912-555	plppr3a	-1.0671526	0.02052212	phospholipid phosphatase related 3a
GeneID:556853	NM_001128688.1	ZDB-GENE-070912-591	cbln2a	-0.7755329	0.02052212	cerebellin 2a precursor
GeneID:791132	NM_001080614.1	ZDB-GENE-070112-252	csrp1b	-0.9539922	0.02052212	cysteine and glycine-rich protein 1b
GeneID:100149863	NM_001103128.1	ZDB-GENE-980526-521	her4.1	-0.8833895	0.0206773	hairy-related 4.1-like
GeneID:100331283	XM_017351874.2	ZDB-GENE-110406-5	otofb	0.73659671	0.02072601	otoferlin b%2C
GeneID:322969	NM_201580.2	ZDB-GENE-030131-1689	nup50	-0.8579432	0.02072601	nucleoporin 50
GeneID:503534	NM_001012498.2	ZDB-GENE-050220-12	rnf7	-1.2143903	0.02072601	ring finger protein 7
GeneID:768293	NM_001077457.1	ZDB-GENE-061027-225	crtc1a	0.95842863	0.02072601	CREB regulated transcription coactivator 1a%2C
GeneID:107980443	NM_001327832.1	ZDB-GENE-040724-74	si:dkey-192d15.2	0.9465865	0.02075257	uncharcterized protein LOC107980443
GeneID:335493	NM_001204250.2	ZDB-GENE-030131-7433	coa3b	-0.6494732	0.02075257	cytochrome C oxidase assembly factor 3b
GeneID:793027	NM_001003589.2	ZDB-GENE-040801-108	ccdc12	-1.0392137	0.02090975	coiled-coil domain containing 12
GeneID:378453	NM_200104.1	ZDB-GENE-030912-14	hnrnpa1b	-0.7447454	0.02091008	heterogeneous nuclear ribonucleoprotein A1b
GeneID:566846	NM_001045422.1	ZDB-GENE-060825-81	dub	-1.0558116	0.02094123	duboraya%2C
GeneID:100534615	NM_001302743.1	ZDB-GENE-110307-1	ost4	-1.1234704	0.02100249	oligosaccharyltransferase complex subunit 4 (non-catalytic)
GeneID:555254	XM_021472895.1	ZDB-GENE-040914-57	itga6l	-1.6624269	0.02105063	integrin%2C alpha 6%2C like%2C
GeneID:558924	NM_001309465.1	ZDB-GENE-031006-12	abca1a	0.96582604	0.02109428	ATP-binding cassette%2C sub-family A (ABC1)%2C member 1A
GeneID:563950	XM_017352670.2	ZDB-GENE-041014-340	si:dkey-221h15.4	1.32759384	0.02109428	si:dkey-221h15.4
GeneID:100330523	NM_001190756.1	ZDB-GENE-030131-968	col5a3a	0.76805915	0.02135312	collagen%2C type V%2C alpha 3a%2C
GeneID:192326	NM_201455.1	ZDB-GENE-020419-6	cct2	-1.6599983	0.02135312	chaperonin containing TCP1%2C subunit 2 (beta)
GeneID:559639	NM_001113797.1	ZDB-GENE-080722-18	socs5b	0.9507478	0.02163383	suppressor of cytokine signaling 5b%2C
GeneID:100332028	XM_002666955.4	ZDB-GENE-100809-1	cpne8	0.969723	0.02164834	copine VIII
GeneID:402943	NM_205646.1	ZDB-GENE-040426-1797	stard10	-1.1609661	0.02164834	StAR-related lipid transfer (START) domain containing 10
GeneID:569113	XM_692476.9	ZDB-GENE-060503-169	kctd3	0.94374128	0.02164834	potassium channel tetramerization domain containing 3
GeneID:735250	NM_001045335.2	ZDB-GENE-060804-3	rpl22l1	-0.9873324	0.02175572	ribosomal protein L22-like 1%2C transcript variant 1
GeneID:100535592	XM_017354481.2			1.0393171	0.0219152	uncharacterized LOC100535592%2C
GeneID:403077	NM_001043323.2	ZDB-GENE-050506-24	h2afvb	-1.0071598	0.0219152	H2A histone family%2C member Vb
GeneID:558956	NM_001204169.1			1.8485902	0.0219152	ISG15 ubiquitin-like modifier
GeneID:751752	NM_001045462.2	ZDB-GENE-060825-200	ccdc51	-1.2059516	0.02195134	coiled-coil domain containing 51
GeneID:562350	NM_001030167.1	ZDB-GENE-041210-245	nup107	-0.8688028	0.02201937	nucleoporin 107
GeneID:101883438	XM_005162454.4			0.79049329	0.02215064	formin-like protein 13
GeneID:560720	XM_009296375.3			0.81940351	0.02232996	DENN/MADD domain containing 1B%2C
GeneID:562525	NM_001004559.2	ZDB-GENE-040912-26	cldn2	0.94289848	0.02241805	claudin 2%2C
GeneID:558150	XM_009291501.3	ZDB-GENE-110411-146	sacs	0.89284034	0.02242796	sacsin molecular chaperone%2C
GeneID:30083	NM_001362264.1	ZDB-GENE-000210-32	nme2b.1	-1.6423888	0.02247136	NME/NM23 nucleoside diphosphate kinase 2b%2C tandem duplicate 1%2C
GeneID:100005981	XM_001344835.8	ZDB-GENE-120406-9	ppp2r5ca	-1.1783295	0.02259213	protein phosphatase 2%2C regulatory subunit B'%2C gamma a
GeneID:30334	NM_131113.1	ZDB-GENE-980526-530	pou6f1	0.82265507	0.02259213	POU class 6 homeobox 1%2C
GeneID:561303	NM_001200011.2	ZDB-GENE-030131-3627	si:ch73-41h24.1	1.02080066	0.02259213	si:ch73-41h24.1%2C
GeneID:569091	XM_009303116.3	ZDB-GENE-141216-311	si:dkey-19b23.7	-0.7047893	0.02259213	si:dkey-19b23.7%2C
GeneID:572250	XM_695946.8	ZDB-GENE-091118-54	cnn1b	0.85804897	0.02262709	calponin 1%2C basic%2C smooth muscle%2C b
GeneID:445033	NM_001003427.1	ZDB-GENE-040801-15	psma1	-1.4139277	0.02264406	proteasome subunit alpha 1
GeneID:571430	XM_695016.8	ZDB-GENE-030131-8516	si:ch211-183d21.1	1.02695237	0.02264406	si:ch211-183d21.1
GeneID:171478	NM_152961.3	ZDB-GENE-020318-2	fabp3	-1.0698767	0.02266827	fatty acid binding protein 3%2C muscle and heart
GeneID:245951	XM_009292910.3	ZDB-GENE-020506-1	ros1	0.88834806	0.02295674	c-ros oncogene 1%2C receptor tyrosine kinase%2C
GeneID:402980	NM_205680.1	ZDB-GENE-040426-1889	zgc:77838	0.74852946	0.02295674	zgc:77838%2C
GeneID:445043	NM_001003437.2	ZDB-GENE-040801-170	syf2	-0.8302952	0.02295674	SYF2 pre-mRNA-splicing factor
GeneID:563166	XM_009304219.3			0.78037834	0.02295674	RIMS binding protein 2%2C
GeneID:336612	NM_212758.1	ZDB-GENE-030131-8556	ppiaa	-1.1804423	0.0230854	peptidylprolyl isomerase Aa (cyclophilin A)
GeneID:564690	NM_001177451.1	ZDB-GENE-080204-76	zgc:174904	1.54501179	0.0230854	zgc:174904
GeneID:569455	NM_001145604.1	ZDB-GENE-030424-2	kpnb3	-1.0078452	0.02321129	karyopherin (importin) beta 3%2C
GeneID:100536119	XM_021466570.1			-1.3530135	0.02325493	NAD-dependent protein deacylase sirtuin-5%2C mitochondrial-like
GeneID:101882211	XM_005161637.4			1.41201196	0.02325493	uncharacterized LOC101882211%2C
GeneID:641321	NM_001037117.2	ZDB-GENE-051030-75	snap23.2	1.28043884	0.02325493	synaptosomal-associated protein 23.2
GeneID:767726	NM_001076664.2	ZDB-GENE-060929-1046	fdx2	-0.9906032	0.02333433	ferredoxin 1-like
GeneID:560522	NM_001110460.1	ZDB-GENE-070424-97	hnrnpd	-0.6759514	0.0233484	heterogeneous nuclear ribonucleoprotein D%2C
GeneID:100006778	NM_001113504.1	ZDB-GENE-080104-6	il6st	0.80309638	0.02335095	interleukin 6 signal transducer%2C
GeneID:100329900	XM_009296561.3	ZDB-GENE-100921-74	frmd4bb	-0.7480443	0.02347369	FERM domain containing 4Bb%2C
GeneID:393871	NM_200897.2	ZDB-GENE-040426-1863	csrp2	-0.7537478	0.02348566	cysteine and glycine-rich protein 2
GeneID:100005083	NM_001082957.2	ZDB-GENE-070822-9	icn2	-1.4861385	0.02368642	ictacalcin 2
GeneID:405789	NM_212863.2	ZDB-GENE-040629-2	sp7	1.26423045	0.02368642	Sp7 transcription factor
GeneID:793646	NM_001167899.1	ZDB-GENE-090407-1	glra2	-0.7354663	0.02368642	glycine receptor%2C alpha 2%2C
GeneID:100334010	XM_002662055.5	ZDB-GENE-060526-76	cenpv	1.07617349	0.02369704	centromere protein V
GeneID:101884964	XM_005157607.4	ZDB-GENE-131127-570	si:ch1073-145m9.1	1.8521703	0.02369704	si:ch1073-145m9.1
GeneID:101885377	XM_005173015.4			-0.7441342	0.02369704	uncharacterized LOC101885377
GeneID:394059	NM_201084.2	ZDB-GENE-040426-821	cdc5l	-1.000582	0.02369704	CDC5 cell division cycle 5-like (S. pombe)
GeneID:394209	XM_002662826.5	ZDB-GENE-040108-9	sb:cb1058	0.95854687	0.02369704	sb:cb1058%2C
GeneID:541418	NM_001013545.2	ZDB-GENE-050320-120	zgc:101564	0.97235458	0.02369704	zgc:101564
GeneID:567822	XM_021467792.1	ZDB-GENE-030616-581	si:ch211-14a17.10	0.91415655	0.02369704	si:ch211-14a17.10%2C
GeneID:571482	NM_001045220.1	ZDB-GENE-060503-468	si:dkeyp-92c9.2	0.94324513	0.02369704	si:dkeyp-92c9.2%2C
GeneID:564048	NM_001089403.1	ZDB-GENE-030131-2752	plekhm1	0.89709879	0.02380376	pleckstrin homology domain containing%2C family M (with RUN domain) member 1%2C
GeneID:445145	NM_001003539.1	ZDB-GENE-040801-49	nudcd2	-0.6429235	0.02394534	NudC domain containing 2
GeneID:561821	NM_001190307.2	ZDB-GENE-100405-2	kcnk18	-1.0507862	0.02411042	potassium channel%2C subfamily K%2C member 18
GeneID:116517	NM_131849.2	ZDB-GENE-011003-1	adh5	-1.2997626	0.02421988	alcohol dehydrogenase 5
GeneID:30345	NM_131122.2	ZDB-GENE-990415-112	hoxc4a	1.98601721	0.02421988	homeobox C4a%2C
GeneID:794129	XM_021466438.1	ZDB-GENE-050522-383	mau2	-1.3778673	0.02421988	MAU2 sister chromatid cohesion factor
GeneID:564371	NM_001195241.1	ZDB-GENE-100901-2	kcnc3b	0.85107429	0.02426127	potassium voltage-gated channel%2C Shaw-related subfamily%2C member 3b%2C
GeneID:325064	NM_212692.1	ZDB-GENE-030131-3789	thoc5	-1.0333973	0.02430612	THO complex 5%2C
GeneID:570700	NM_001037702.3	ZDB-GENE-030131-2579	kpnb1	-1.1043713	0.02430612	karyopherin (importin) beta 1
GeneID:692332	NM_001045298.1	ZDB-GENE-060519-40	cyp46a1.3	0.83359001	0.0243144	cytochrome P450%2C family 46%2C subfamily A%2C polypeptide 1%2C tandem duplicate 3
GeneID:559570	XM_009291752.3	ZDB-GENE-090807-2	mnta	0.84107814	0.02433263	MAX network transcriptional repressor a%2C
GeneID:30221	NM_131024.1	ZDB-GENE-991119-4	psen1	-0.7028342	0.02442442	presenilin 1%2C
GeneID:30369	NM_131139.1	ZDB-GENE-990415-223	fzd7a	-0.9588736	0.0244428	frizzled class receptor 7a
GeneID:334100	NM_199858.2	ZDB-GENE-030131-6032	ldb3b	-1.2353984	0.02451797	LIM domain binding 3b%2C
GeneID:100004977	NM_001002181.1	ZDB-GENE-040704-20	mthfd2	0.83067166	0.0245667	methylenetetrahydrofolate dehydrogenase (NADP+ dependent) 2%2C methenyltetrahydrofolate cyclohydrolase
GeneID:101882984	XM_021468928.1			1.16085724	0.0245667	si:ch211-134a4.4
GeneID:393599	NM_200626.1	ZDB-GENE-040426-1272	ddb1	-1.3892966	0.0245667	damage-specific DNA binding protein 1
GeneID:393652	NM_200679.3	ZDB-GENE-040426-1414	lyar	-0.8381938	0.0245667	Ly1 antibody reactive homolog (mouse)
GeneID:563246	NM_001144794.1	ZDB-GENE-081028-71	exoc3l4	0.78374238	0.0245667	exocyst complex component 3-like 4%2C
GeneID:393214	NM_200244.1	ZDB-GENE-040426-884	plekhf2	0.68893494	0.02458366	pleckstrin homology domain containing%2C family F (with FYVE domain) member 2%2C
GeneID:108180146	XM_021468272.1			-1.284604	0.0245847	uncharacterized LOC108180146
GeneID:550231	NM_001017569.1	ZDB-GENE-050417-22	cdk5r2a	-1.102606	0.0245847	cyclin-dependent kinase 5%2C regulatory subunit 2a (p39)
GeneID:561049	NM_001044951.1	ZDB-GENE-050208-283	eif3eb	-0.6247258	0.02478697	eukaryotic translation initiation factor 3%2C subunit E%2C b
GeneID:768194	NM_001077336.1	ZDB-GENE-061013-283	pick1	0.87787284	0.02478697	protein interacting with prkca 1
GeneID:792402	NM_001002224.2	ZDB-GENE-040704-71	kctd4	-1.0017456	0.02478697	potassium channel tetramerization domain containing 4
GeneID:30541	NM_131284.2	ZDB-GENE-990415-78	foxa1	-0.715057	0.02502136	forkhead box A1
GeneID:406449	NM_213170.1	ZDB-GENE-040426-2200	ptges3a	0.79251292	0.02502136	prostaglandin E synthase 3a (cytosolic)
GeneID:795198	XM_005157928.4	ZDB-GENE-100922-123	si:dkey-27n14.1	1.01562007	0.02502136	si:dkey-27n14.1
GeneID:797303	NM_001077141.1	ZDB-GENE-060929-508	tada2a	-0.5573417	0.02508404	transcriptional adaptor 2A%2C
GeneID:436819	NM_001002546.1	ZDB-GENE-040718-281	vapal	-1.0757163	0.02520653	VAMP (vesicle-associated membrane protein)-associated protein A%2C like
GeneID:449822	NM_001005995.2	ZDB-GENE-041010-72	zgc:101569	-0.7483427	0.02520653	zgc:101569%2C transcript variant 1
GeneID:571011	XM_009295180.3			0.86798489	0.02520653	phosphodiesterase 8B%2C
GeneID:101886833	XM_021471646.1			1.65103731	0.02541638	C-X-C motif chemokine 11-like

GeneID:100149555	XM_001921870.7	ZDB-GENE-111111-11	stard9	0.9202906	0.02559841	StAR-related lipid transfer (START) domain containing 9
GeneID:100536024	XM_003197803.4	ZDB-GENE-130530-628	zmp:0000000625	-1.1724208	0.02559841	zmp:0000000625
GeneID:393994	NM_001320519.1	ZDB-GENE-040426-1326	ttll1	-1.1314985	0.02559841	tubulin tyrosine ligase-like family%2C member 1%2C
GeneID:563283	NM_001110463.1	ZDB-GENE-060503-433	plekho1a	-0.77509	0.02559841	pleckstrin homology domain containing%2C family O member 1a
GeneID:101882488	XM_003197942.5			0.89133004	0.02562485	tripartite motif-containing protein 16-like
GeneID:558206	XM_005158277.4	ZDB-GENE-080425-4	cspg5a	-0.7191648	0.02562485	chondroitin sulfate proteoglycan 5a%2C
GeneID:393533	NM_200561.2	ZDB-GENE-040426-1451	fzd6	0.802313	0.02576903	frizzled class receptor 6
GeneID:560620	NM_001114419.1	ZDB-GENE-050208-185	hemk1	-0.7782633	0.0258731	HemK methyltransferase family member 1
GeneID:100332402	XM_021466504.1			-1.0699075	0.02595263	dynein cytoplasmic 2 heavy chain 1
GeneID:101887100	XM_005168430.4			0.81469499	0.02595263	uncharacterized LOC101887100
GeneID:436733	NM_001002460.2	ZDB-GENE-040718-161	zgc:92907	-0.752576	0.02595263	zgc:92907
GeneID:566945	NM_194414.1	ZDB-GENE-030901-2	mmp14b	0.77474246	0.02595263	matrix metallopeptidase 14b (membrane-inserted)%2C
GeneID:447892	NM_001025558.2	ZDB-GENE-040912-57	zgc:101731	-0.9007522	0.02596241	zgc:101731
GeneID:100329294	NM_001195441.3			-1.9675838	0.02600888	uncharacterized LOC100329294%2C
GeneID:114420	NM_200852.3	ZDB-GENE-010724-15	rps9	-1.4733451	0.02600888	ribosomal protein S9
GeneID:557109	NM_001199755.1	ZDB-GENE-090826-2	raf1b	-0.647007	0.02600888	Raf-1 proto-oncogene%2C serine/threonine kinase b%2C
GeneID:566587	XM_689863.9			0.83994269	0.02601749	ERBB receptor feedback inhibitor 1-like
GeneID:406303	NM_213030.1	ZDB-GENE-040426-1970	tuba2	-0.8760637	0.02606029	tubulin%2C alpha 2
GeneID:562078	XM_005160274.4	ZDB-GENE-141212-230	si:ch211-151p13.8	0.79666724	0.02606029	si:ch211-151p13.8
GeneID:564052	NM_001145582.2	ZDB-GENE-080723-57	lingo2b	-0.7153863	0.02606029	leucine rich repeat and Ig domain containing 2b%2C
GeneID:796857	XM_001337212.7	ZDB-GENE-060503-115	magixa	0.68786458	0.02606029	MAGI family member%2C X-linked a%2C
GeneID:445300	NM_001003756.1	ZDB-GENE-040808-12	meaf6	-0.5951939	0.02616666	MYST/Esa1-associated factor 6%2C
GeneID:791107	NM_001080203.1	ZDB-GENE-070103-2	hs6st1b	-1.2678285	0.02616666	heparan sulfate 6-O-sulfotransferase 1b
GeneID:565781	XM_689051.8	ZDB-GENE-100910-2	sst1.2	-0.9263925	0.02635013	somatostatin 1%2C tandem duplicate 2
GeneID:436885	NM_001002612.1	ZDB-GENE-040718-356	zgc:92275	-1.1928181	0.02636323	zgc:92275
GeneID:777744	NM_001077788.2	ZDB-GENE-061027-53	rab3gap1	-1.0631278	0.02644581	RAB3 GTPase activating protein subunit 1
GeneID:568283	XM_009302582.3	ZDB-GENE-100729-2	larp4ab	0.76680386	0.02652653	La ribonucleoprotein domain family%2C member 4Ab%2C
GeneID:564838	NM_001045853.1	ZDB-GENE-060825-331	ppp1r14ba	-0.6556095	0.02668647	protein phosphatase 1%2C regulatory (inhibitor) subunit 14Ba%2C
GeneID:793666	NM_001020800.1	ZDB-GENE-060518-2	popdc2	1.14788303	0.02668647	popeye domain containing 2%2C transcript variant 1
GeneID:793969	XM_005158551.4	ZDB-GENE-131121-492	ccser2a	0.54226794	0.02669071	coiled-coil serine-rich protein 2a%2C
GeneID:336619	NM_214718.1	ZDB-GENE-030131-8563	eif4bb	-0.7523472	0.02717751	eukaryotic translation initiation factor 4Bb%2C
GeneID:387290	NM_001328186.1	ZDB-GENE-031201-3	smox	0.79793861	0.02717751	spermine oxidase
GeneID:100334344	NM_001291900.1	ZDB-GENE-141222-6	si:ch211-195b11.3	1.10539538	0.02718839	si:ch211-195b11.3
GeneID:570405	XM_017356957.2			1.20317431	0.02718839	histone H3-like
GeneID:393274	NM_200304.1	ZDB-GENE-040426-1110	strap	-0.6407805	0.02719784	serine/threonine kinase receptor associated protein
GeneID:570792	NM_001201392.1	ZDB-GENE-080204-57	myrf	0.78163113	0.02727092	myelin regulatory factor%2C
GeneID:324723	NM_212687.1	ZDB-GENE-040426-1968	irf2bpl	-0.474216	0.02731464	interferon regulatory factor 2 binding protein-like
GeneID:337158	NM_199214.1	ZDB-GENE-030131-9102	col1a1a	-1.0126108	0.02731464	collagen%2C type I%2C alpha 1a
GeneID:100005105	XM_021476961.1			-1.4984087	0.02745887	meiosis regulator and mRNA stability factor 1%2C
GeneID:796551	NM_001083866.1	ZDB-GENE-080405-1	tert	-1.3204145	0.02745887	telomerase reverse transcriptase
GeneID:100334264	XM_002663670.5			0.79133898	0.02748711	multiple C2 domains%2C transmembrane 1b%2C
GeneID:100333757	XM_002667837.6	ZDB-GENE-110721-2	ggt5b	1.13629711	0.02752462	gamma-glutamyltransferase 5b
GeneID:570586	XM_694083.8	ZDB-GENE-121023-2	msantd4	0.70219036	0.02752462	Myb/SANT-like DNA-binding domain containing 4 with coiled-coils
GeneID:798302	XM_005157179.4	ZDB-GENE-100316-9	frmpd3	0.60808918	0.02752462	FERM and PDZ domain containing 3%2C
GeneID:553443	NM_001083819.1	ZDB-GENE-060810-69	vstm4a	-1.0004479	0.02759061	V-set and transmembrane domain containing 4a%2C
GeneID:100004929	XM_001344071.8	ZDB-GENE-070912-124	zeb1a	0.69586047	0.02766825	zinc finger E-box binding homeobox 1a%2C
GeneID:114834	NM_131848.2	ZDB-GENE-010919-2	tfe3a	1.0871729	0.02766825	transcription factor binding to IGHM enhancer 3a
GeneID:30574	NM_131311.2	ZDB-GENE-980526-212	dlx2a	-0.6506584	0.02766825	distal-less homeobox 2a
GeneID:30622	NM_131351.2	ZDB-GENE-990614-17	ube2ib	-0.748386	0.02766825	ubiquitin-conjugating enzyme E2Ib%2C
GeneID:564461	NM_001287547.1	ZDB-GENE-060201-5	grm2b	-0.6424939	0.02766825	glutamate receptor%2C metabotropic 2b%2C
GeneID:30466	NM_131219.1	ZDB-GENE-980526-90	ascl1a	-0.7580319	0.02767698	achaete-scute family bHLH transcription factor 1a
GeneID:100006618	NM_001017902.1	ZDB-GENE-050417-462	mettl2a	-0.6889979	0.02773887	methyltransferase like 2A
GeneID:406852	NM_213531.1	ZDB-GENE-040426-2941	nup133	-0.9685625	0.02789056	nucleoporin 133
GeneID:393754	NM_200781.1	ZDB-GENE-040426-1750	chmp7	-0.7329378	0.02800356	charged multivesicular body protein 7
GeneID:394081	NM_201106.1	ZDB-GENE-040426-1287	bysl	-1.6563159	0.02816523	bystin-like
GeneID:678599	NM_001040347.1	ZDB-GENE-060421-3674	zgc:136971	-0.6729744	0.02816523	zgc:136971%2C
GeneID:100007978	NM_001082990.1	ZDB-GENE-060503-68	si:dkey-261i16.5	-0.6898971	0.02819473	si:dkey-261i16.5%2C
GeneID:100334928	XM_009293931.3	ZDB-GENE-120404-1	tgfbr2a	0.99846927	0.02819473	transforming growth factor beta receptor 2a
GeneID:570262	XM_017352030.2	ZDB-GENE-050420-284	srgap1a	0.65612919	0.02831482	SLIT-ROBO Rho GTPase activating protein 1a%2C
GeneID:323683	XM_001335483.7	ZDB-GENE-030131-2403	tut4	-1.7173306	0.02838269	zinc finger%2C CCHC domain containing 11%2C
GeneID:100536289	XM_003201459.5	ZDB-GENE-131127-365	fam19a5b	-0.7065593	0.02844024	family with sequence similarity 19 (chemokine (C-C motif)-like)%2C member A5b
GeneID:393636	NM_001328426.1	ZDB-GENE-040426-1611	tiprl	-0.9819372	0.02844024	TIP41%2C TOR signaling pathway regulator-like (S. cerevisiae)
GeneID:406593	NM_214749.1	ZDB-GENE-040426-2515	nhej1	-0.9956519	0.02874904	nonhomologous end-joining factor 1
GeneID:494103	NM_001008646.1	ZDB-GENE-041212-74	cdk11b	-0.8660569	0.02877698	cyclin-dependent kinase 11B%2C
GeneID:572733	NM_201024.1	ZDB-GENE-040116-6	pgam2	-1.5848838	0.02885158	phosphoglycerate mutase 2 (muscle)
GeneID:100750260	NM_001251832.1			-1.4296154	0.02891294	polyribonucleotide nucleotidyltransferase 1
GeneID:336124	NM_001089341.1	ZDB-GENE-030131-8068	cnot11	-0.7701956	0.02891294	CCR4-NOT transcription complex%2C subunit 11
GeneID:336198	NM_200001.2	ZDB-GENE-030131-8142	ppp4r3b	-1.4443229	0.02891294	protein phosphatase 4%2C regulatory subunit 3B
GeneID:406496	NM_213215.1	ZDB-GENE-040426-2299	aldoab	-1.2843648	0.02891294	aldolase a%2C fructose-bisphosphate%2C b
GeneID:541385	NM_001013512.2	ZDB-GENE-050320-81	tmem86a	1.04893332	0.02891294	transmembrane protein 86A
GeneID:58153	NM_131629.1	ZDB-GENE-000511-8	slc40a1	0.59520601	0.02891294	solute carrier family 40 (iron-regulated transporter)%2C member 1
GeneID:108180736	XM_017352938.2	ZDB-GENE-070806-4	or113-4	-1.226608	0.02892107	olfactory receptor 2A1/2A42-like
GeneID:558729	XM_009292581.3	ZDB-GENE-060503-427	cpne4a	-0.5103882	0.02892107	copine IVa
GeneID:557524	XM_679124.7			0.91419646	0.02895433	un-named sa1261
GeneID:563753	XM_021478463.1	ZDB-GENE-070817-3	mmp11a	0.98698533	0.02895433	matrix metallopeptidase 11a
GeneID:393608	NM_200635.1	ZDB-GENE-040426-1321	ptger2a	1.14171201	0.02909819	prostaglandin E receptor 2a (subtype EP2)
GeneID:100537129	XM_017351172.2			0.75094341	0.02920062	si:dkey-103g5.4%2C
GeneID:100148522	XM_001919861.6	ZDB-GENE-121214-95	si:ch211-217a12.1	-1.259517	0.02940564	si:ch211-217a12.1%2C
GeneID:100332066	XM_002661256.6	ZDB-GENE-131121-348	ppp1r27a	-1.5352278	0.02940564	protein phosphatase 1%2C regulatory subunit 27a
GeneID:114421	NM_131818.3	ZDB-GENE-010817-3	angptl3	0.98209265	0.02940564	angiopoietin-like 3%2C
GeneID:393673	NM_200700.1	ZDB-GENE-040426-1657	desi1a	0.73147391	0.02940564	desumoylating isopeptidase 1a
GeneID:100148073	NM_001142376.1	ZDB-GENE-030131-4473	caskb	-1.3632214	0.02944035	calcium/calmodulin-dependent serine protein kinase b
GeneID:101887127	XM_005160119.4			1.49475507	0.02944035	calcium homeostasis modulator 1
GeneID:30750	XM_694808.9	ZDB-GENE-990415-212	pparda	1.08061146	0.02944035	peroxisome proliferator-activated receptor delta a
GeneID:406297	NM_213024.1	ZDB-GENE-040426-1961	dynll1	-0.9970088	0.02944035	dynein%2C light chain%2C LC8-type 1%2C
GeneID:492579	NM_001309483.1	ZDB-GENE-070828-2	efcab6	-0.8056961	0.02944035	EF-hand calcium binding domain 6
GeneID:393122	NM_200153.1	ZDB-GENE-040426-852	dnpep	-0.7058847	0.02952844	aspartyl aminopeptidase%2C
GeneID:100006201	XM_001339351.8	ZDB-GENE-090521-4	letm2	-0.8909414	0.02965942	leucine zipper-EF-hand containing transmembrane protein 2%2C
GeneID:192328	NM_177479.2	ZDB-GENE-020419-1	u2af1	-0.6248707	0.02965942	U2 small nuclear RNA auxiliary factor 1
GeneID:334608	NM_199918.2	ZDB-GENE-030131-6540	vapa	-1.5226938	0.02965942	VAMP (vesicle-associated membrane protein)-associated protein A
GeneID:336550	NM_201153.1	ZDB-GENE-030131-8494	rps3	-1.5958654	0.02965942	ribosomal protein S3
GeneID:406250	NM_001003944.1	ZDB-GENE-040428-2	actr3	-1.4635513	0.02965942	ARP3 actin related protein 3 homolog
GeneID:550463	NM_001017766.2	ZDB-GENE-050417-287	eed	-1.3379473	0.02965942	embryonic ectoderm development
GeneID:553132	NM_001020503.2	ZDB-GENE-050512-1	syce2	0.94717372	0.02965942	synaptonemal complex central element protein 2
GeneID:559502	NM_001310844.1	ZDB-GENE-070705-13	mrc1b	1.14736531	0.02965942	mannose receptor%2C C type 1b
GeneID:562930	XM_001919000.6	ZDB-GENE-070815-4	papln a	-1.1522564	0.02965942	papilin a%2C proteoglycan-like sulfated glycoprotein%2C
GeneID:565714	NM_001025540.1	ZDB-GENE-051129-1	wnt7aa	-0.7458549	0.02965942	wingless-type MMTV integration site family%2C member 7Aa
GeneID:798206	NM_001128796.1	ZDB-GENE-030131-4878	zgc:194551	-1.7663466	0.02965942	zgc:194551
GeneID:568689	XM_021474083.1	ZDB-GENE-090310-1	card14	-0.9292233	0.02972073	caspase recruitment domain family%2C member 14
GeneID:393263	NM_200293.2	ZDB-GENE-040426-1050	ddrgk1	-1.1513226	0.02995868	DDRGK domain containing 1
GeneID:570947	XM_001919051.7	ZDB-GENE-081105-71	dennd2c	-0.5704051	0.02995868	DENN/MADD domain containing 2C
GeneID:100334988	XM_002663842.5	ZDB-GENE-131115-1	trappc10	-0.8101401	0.02999355	trafficking protein particle complex 10
GeneID:563306	NM_001083551.1	ZDB-GENE-070319-1	rbpjb	0.77246294	0.02999355	recombination signal binding protein for immunoglobulin kappa J region b
GeneID:569667	NM_001045187.1	ZDB-GENE-040724-21	zdhhc14	0.87247985	0.03006999	zinc finger%2C DHHC-type containing 14%2C

GeneID:100151589	XM_001923942.6	ZDB-GENE-130530-729	si:dkey-183j2.10	-0.8637201	0.03010022	si:dkey-183j2.10
GeneID:100330213	XM_002661359.4	ZDB-GENE-070705-151	si:ch211-258f14.2	-0.6759211	0.03010022	si:ch211-258f14.2%2C
GeneID:553749	NM_001020720.1	ZDB-GENE-050522-529	zgc:113279	0.85855962	0.03010022	zgc:113279%2C
GeneID:569734	NM_001123298.1	ZDB-GENE-080226-1	ggt5a	1.08423972	0.03010022	gamma-glutamyltransferase 5a%2C
GeneID:641560	NM_001037559.1	ZDB-GENE-051127-5	slc7a7	1.02827079	0.03010022	solute carrier family 7 (amino acid transporter light chain%2C y+L system)%2C member 7
GeneID:794084	NM_001327912.1	ZDB-GENE-130603-13	si:ch73-111m19.2	1.07460315	0.03010022	si:ch73-111m19.2%2C
GeneID:100329491	XM_021475185.1			-1.7318896	0.03019874	si: zfos-932h1.3%2C
GeneID:322372	NM_199601.1	ZDB-GENE-030131-1091	fah	1.27785919	0.0303476	fumarylacetoacetate hydrolase (fumarylacetoacetase)%2C
GeneID:393215	NM_200245.1	ZDB-GENE-040426-886	rbbp5	-0.764078	0.03044077	retinoblastoma binding protein 5%2C
GeneID:563665	NM_001100013.1	ZDB-GENE-030131-2211	c3b.1	0.90978689	0.03044077	complement component c3b%2C tandem duplicate 1%2C
GeneID:564554	NM_001110347.1	ZDB-GENE-130530-866	lrig3	-1.5594565	0.03083529	leucine-rich repeats and immunoglobulin-like domains 3
GeneID:393195	NM_200226.2	ZDB-GENE-040426-976	rnaseh2a	-1.1624819	0.03091481	ribonuclease H2%2C subunit A
GeneID:100034609	XM_003200774.5			0.7082351	0.03109618	dual specificity phosphatase 10
GeneID:30288	NM_131079.2	ZDB-GENE-980526-144	her6	-0.8247585	0.03113704	hairy-related 6
GeneID:406578	NM_213289.1	ZDB-GENE-040426-2482	stxbp2	0.65233539	0.03113704	syntaxin binding protein 2
GeneID:566553	XM_005164274.4	ZDB-GENE-071214-1	recql5	-0.9813734	0.03113704	RecQ helicase-like 5
GeneID:393710	NM_200737.1	ZDB-GENE-040426-1700	faua	-1.7428867	0.03116765	Finkel-Biskis-Reilly murine sarcoma virus (FBR-MuSV) ubiquitously expressed a
GeneID:335357	NM_199948.1	ZDB-GENE-030131-7297	syt4	-1.0483048	0.03122678	synaptotagmin IV
GeneID:503767	NM_001013345.1	ZDB-GENE-050306-51	rbm8a	-0.5905187	0.03127151	RNA binding motif protein 8A
GeneID:556465	NM_001089324.1	ZDB-GENE-030131-1092	mtif2	-0.6090432	0.03127151	mitochondrial translational initiation factor 2
GeneID:565980	XM_689244.9	ZDB-GENE-030131-1826	ace	-0.8975853	0.03127151	angiotensin I converting enzyme (peptidyl-dipeptidase A) 1
GeneID:724001	NM_001045306.1	ZDB-GENE-030131-66	dnajc8	-1.1582047	0.03127151	DnaJ (Hsp40) homolog%2C subfamily C%2C member 8
GeneID:791156	NM_001080638.1	ZDB-GENE-070112-1072	slc25a15a	1.12496965	0.03127151	solute carrier family 25 (mitochondrial carrier%3B ornithine transporter) member 15a
GeneID:568631	NM_001034984.1	ZDB-GENE-060526-274	slc4a4a	0.67207276	0.03127733	solute carrier family 4 (sodium bicarbonate cotransporter)%2C member 4a%2C
GeneID:30230	NM_131032.2	ZDB-GENE-990415-83	inab	-0.7040478	0.03151109	internexin neuronal intermediate filament protein%2C alpha b
GeneID:553661	NM_001020634.1	ZDB-GENE-050522-417	homer2	-1.2376892	0.03158962	homer scaffolding protein 2
GeneID:30670	NM_131396.1	ZDB-GENE-990415-263	thraa	0.76560511	0.03173653	thyroid hormone receptor alpha a%2C
GeneID:436823	NM_001002550.2	ZDB-GENE-040718-288	pir	0.60178009	0.03173653	pirin
GeneID:570552	XM_005156924.4	ZDB-GENE-090313-170	si:dkey-103j14.5	1.0911901	0.03173653	si:dkey-103j14.5%2C
GeneID:768286	NM_001077451.2	ZDB-GENE-061027-93	ccdc120	0.69464284	0.03178829	coiled-coil domain containing 120%2C
GeneID:568779	NM_001080604.1	ZDB-GENE-070112-1992	zbtb20	1.02315004	0.03190791	zinc finger and BTB domain containing 20%2C
GeneID:334166	NM_214705.1			0.81626841	0.03190985	family with sequence similarity 43%2C member A
GeneID:100329773	XM_005173042.4			-0.7104178	0.03203759	transmembrane protein 121-like
GeneID:405872	NM_212936.1	ZDB-GENE-040426-2418	nptx1l	-0.6261023	0.03206469	neuronal pentraxin 1 like
GeneID:562552	NM_001111167.1	ZDB-GENE-030131-4309	zgc:171775	1.39693686	0.03206469	zgc:171775%2C
GeneID:100000085	XM_002662803.5	ZDB-GENE-030131-1312	si:ch211-212k18.7	0.81128058	0.03217682	si:ch211-212k18.7
GeneID:393505	NM_200533.2	ZDB-GENE-040426-1415	srsf10b	-1.3221985	0.0325341	serine/arginine-rich splicing factor 10b
GeneID:406519	NM_214740.1	ZDB-GENE-040426-2347	slc10a3	-0.8557482	0.03257077	solute carrier family 10%2C member 3
GeneID:553609	NM_001177743.2	ZDB-GENE-050522-312	gng8	-1.2164155	0.03257077	guanine nucleotide binding protein (G protein)%2C gamma 8
GeneID:554230	NM_001024653.2	ZDB-GENE-041001-112	kdr	0.87559941	0.03257077	kinase insert domain receptor (a type III receptor tyrosine kinase)
GeneID:558261	NM_001044856.2	ZDB-GENE-041014-253	ociad2	0.94485195	0.03257077	OCIA domain containing 2
GeneID:327635	NM_199841.2	ZDB-GENE-030131-5846	gnsb	0.95035209	0.0325729	glucosamine (N-acetyl)-6-sulfatase (Sanfilippo disease IIID)%2C b
GeneID:101884756	XM_005173869.4			0.92073049	0.03264982	trichohyalin-like%2C
GeneID:324950	NM_001123045.1	ZDB-GENE-030131-3673	tspan17	0.81403836	0.03266788	tetraspanin 17%2C
GeneID:562909	XM_009305138.3			0.77182538	0.03266788	PTC7 protein phosphatase homolog b
GeneID:503762	NM_001013340.1	ZDB-GENE-050306-43	fam210ab	0.76133207	0.03292287	family with sequence similarity 210%2C member Ab
GeneID:563496	NM_001030175.1	ZDB-GENE-050706-134	zfand3	-0.7610268	0.03292287	zinc finger%2C AN1-type domain 3
GeneID:60640	NM_131642.2	ZDB-GENE-001103-4	cyp19a1b	-0.7780921	0.03292287	cytochrome P450%2C family 19%2C subfamily A%2C polypeptide 1b
GeneID:677757	NM_001040051.1	ZDB-GENE-060331-97	mycbp	-1.3775807	0.03292287	c-myc binding protein
GeneID:100334297	XM_005169434.4			0.88235127	0.03299071	potassium/sodium hyperpolarization-activated cyclic nucleotide-gated channel 1-like%2C
GeneID:266799	NM_199531.1	ZDB-GENE-021015-1	gatm	-1.736331	0.03299071	glycine amidinotransferase (L-arginine:glycine amidinotransferase)
GeneID:404604	NM_001328259.1	ZDB-GENE-040426-2343	clasp2	-0.7979138	0.03299071	cytoplasmic linker associated protein 2%2C transcript variant 1
GeneID:550410	NM_001017715.1	ZDB-GENE-050417-216	zgc:112183	-0.6315933	0.03299071	zgc:112183
GeneID:559769	NM_001045848.1	ZDB-GENE-060825-263	tssc4	-0.6649577	0.03299071	tumor suppressing subtransferable candidate 4%2C
GeneID:564523	NM_001110467.1	ZDB-GENE-070424-62	sgk3	0.79091867	0.03299071	serum/glucocorticoid regulated kinase family%2C member 3
GeneID:569505	XM_017351260.2	ZDB-GENE-121214-56	si:ch211-191a16.5	1.52603322	0.03299071	si:ch211-191a16.5
GeneID:799923	XM_009301593.3			0.62113558	0.03334337	si:ch211-208h16.4%2C
GeneID:641475	NM_001037416.2	ZDB-GENE-030516-6	clybl	-0.9814132	0.03340506	citrate lyase beta like
GeneID:553421	XM_021478817.1			1.03739474	0.03343035	si:ch73-167c12.2
GeneID:564996	NM_001045066.1	ZDB-GENE-010919-3	tfe3b	0.93822636	0.03343433	transcription factor binding to IGHM enhancer 3b%2C
GeneID:103910622	XM_009300621.3	ZDB-GENE-141212-227	si:ch211-142b24.6	1.05883538	0.03349582	si:ch211-142b24.6%2C
GeneID:108182684	XM_021470971.1			0.7346604	0.03349582	inhibitor of nuclear factor kappa-B kinase subunit beta-like
GeneID:406246	NM_001009888.2	ZDB-GENE-040429-1	sox21b	-0.6782495	0.03356166	SRY (sex determining region Y)-box 21b
GeneID:436702	NM_001002429.1	ZDB-GENE-040718-126	wdr83	-0.8561202	0.03371711	WD repeat domain containing 83
GeneID:445251	NM_001003645.2	ZDB-GENE-040724-77	carm1	-0.6156665	0.03371711	coactivator-associated arginine methyltransferase 1%2C
GeneID:550383	NM_001017688.1	ZDB-GENE-050417-177	npepl1	-0.8060424	0.03378954	aminopeptidase-like 1
GeneID:568454	XM_009306727.3	ZDB-GENE-100316-5	ablim2	0.88430103	0.03378954	actin binding LIM protein family%2C member 2%2C
GeneID:557828	NM_001256641.1	ZDB-GENE-050419-55	panx2	0.70085617	0.03382595	pannexin 2%2C
GeneID:324340	NM_212673.1	ZDB-GENE-030131-3060	cyp3c1	0.6289742	0.03384873	cytochrome P450%2C family 3%2C subfamily c%2C polypeptide 1
GeneID:100148840	XM_001920184.7	ZDB-GENE-081006-1	wnt7bb	-0.6703463	0.03395392	wingless-type MMTV integration site family%2C member 7Bb%2C
GeneID:100334925	XM_009292700.3	ZDB-GENE-131127-642	si:dkeyp-97a10.3	-2.0283239	0.03395392	si:dkeyp-97a10.3
GeneID:101883513	XM_009298298.3			0.8577937	0.03399463	gastrula zinc finger protein XlCGF57.1-like
GeneID:100006464	XM_001345161.6	ZDB-GENE-121214-264	si:dkey-27j5.5	0.78490525	0.03402545	si:dkey-27j5.5
GeneID:393567	NM_200595.1	ZDB-GENE-040426-1450	fbxo45	-0.6650323	0.03402545	F-box protein 45
GeneID:402995	NM_001122698.1	ZDB-GENE-060620-1	pus7	-1.2365802	0.03402545	pseudouridylate synthase 7 (putative)
GeneID:550235	NM_001017573.2	ZDB-GENE-050417-28	psmd5	-0.8942342	0.03402545	proteasome 26S subunit%2C non-ATPase 5
GeneID:569539	NM_001128751.1	ZDB-GENE-030131-1862	fignl1	-0.9538568	0.03402545	fidgetin-like 1%2C
GeneID:796017	NM_001200036.1	ZDB-GENE-141216-219	si:ch211-215a9.5	-0.9400696	0.03402545	si:ch211-215a9.5
GeneID:100000044	XM_001340292.7	ZDB-GENE-121214-60	kcnj19b	-0.6898409	0.03425936	si:dkey-100n10.2
GeneID:393461	NM_001102475.2	ZDB-GENE-050419-45	gmds	-0.8162291	0.03426381	GDP-mannose 4%2C6-dehydratase%2C transcript variant 1
GeneID:192301	NM_173235.3	ZDB-GENE-020419-25	rpl24	-1.6443091	0.0342669	ribosomal protein L24
GeneID:100333891	XM_002666373.6			0.76621889	0.03441584	hepatic and glial cell adhesion molecule%2C
GeneID:563719	XM_009291168.3	ZDB-GENE-150311-1	foxo4	0.80819241	0.03449974	forkhead box O4
GeneID:100003678	XM_001336753.7	ZDB-GENE-131127-232	si:ch211-286c4.6	-1.0112074	0.03450852	si:ch211-286c4.6%2C
GeneID:100189615	NM_001135142.1	ZDB-GENE-081022-111	zgc:194398	1.12858339	0.03450852	zgc:194398
GeneID:100334731	XM_021467528.1			-0.7556702	0.03450852	ryanodine receptor 3-like
GeneID:100537687	XM_017355404.2	ZDB-GENE-161017-46	si:cabz01054396.2	-0.9023732	0.03450852	si:cabz01054396.2
GeneID:110438817	XM_021472113.1			-1.7327784	0.03450852	E3 ubiquitin-protein ligase RBBP6-like%2C
GeneID:322256	NM_199591.2	ZDB-GENE-030131-975	eif5	-0.7446574	0.03450852	eukaryotic translation initiation factor 5%2C
GeneID:368239	NM_213301.2	ZDB-GENE-030326-5	aldh2.2	-0.9274662	0.03450852	aldehyde dehydrogenase 2 family (mitochondrial)%2C tandem duplicate 2
GeneID:393182	NM_200213.1	ZDB-GENE-040426-947	prpf4ba	0.59219758	0.03450852	pre-mRNA processing factor 4Ba
GeneID:436588	NM_001002317.2	ZDB-GENE-040714-2	polr2d	-1.2043574	0.03450852	polymerase (RNA) II (DNA directed) polypeptide D
GeneID:445306	NM_001003763.2	ZDB-GENE-040808-19	eif3ha	-1.211834	0.03450852	eukaryotic translation initiation factor 3%2C subunit H%2C a
GeneID:492328	NM_001007294.1	ZDB-GENE-041114-3	ankrd9	-1.0731386	0.03450852	ankyrin repeat domain 9
GeneID:493610	NM_001007770.1	ZDB-GENE-041121-4	med28	-0.5558914	0.03450852	mediator complex subunit 28
GeneID:567758	NM_001317766.1	ZDB-GENE-050419-197	carmil2	-1.1682598	0.03450852	RGD motif%2C leucine rich repeats%2C tropomodulin domain and proline-rich containing
GeneID:751670	NM_001045387.1	ZDB-GENE-060825-210	arl9	-0.6277559	0.03450852	ADP-ribosylation factor-like 9
GeneID:751683	NM_001045397.1	ZDB-GENE-060503-173	si:dkey-6n6.2	-0.9807445	0.03450852	si:dkey-6n6.2
GeneID:799482	NM_212669.1	ZDB-GENE-050417-309	sypl2b	1.07695516	0.03450852	synaptophysin-like 2b
GeneID:393105	NM_200136.1	ZDB-GENE-040421-1	hsd17b4	-1.050365	0.03465127	hydroxysteroid (17-beta) dehydrogenase 4%2C
GeneID:794708	XM_017351515.2	ZDB-GENE-090806-2	mroh1	0.66828978	0.03465127	maestro heat-like repeat family member 1%2C
GeneID:393944	NM_200969.1	ZDB-GENE-040426-1615	sncb	-0.650308	0.03471845	synuclein%2C beta
GeneID:394122	NM_201147.2	ZDB-GENE-040426-1066	mrpl14	-0.9708935	0.03471845	mitochondrial ribosomal protein L14
GeneID:353150	NM_180969.2	ZDB-GENE-030427-1	cntn1a	-1.3758755	0.03496134	contactin 1a

GeneID:406735	NM_213426.1	ZDB-GENE-040426-2766	hnrnpr	-0.8072868	0.03496134	heterogeneous nuclear ribonucleoprotein R
GeneID:541328	NM_001013456.1	ZDB-GENE-050320-17	ndufa5	-1.3948747	0.03496134	NADH dehydrogenase (ubiquinone) 1 alpha subcomplex%2C 5
GeneID:554098	NM_001024399.1	ZDB-GENE-050522-113	mrpl28	-1.0860477	0.03496134	mitochondrial ribosomal protein L28
GeneID:565078	XM_688372.6	ZDB-GENE-141215-49	si:dkey-33m11.8	-1.056068	0.03496134	si:dkey-33m11.8
GeneID:791986	NM_001002527.3	ZDB-GENE-040718-260	sec61b	-0.7712753	0.03496134	Sec61 translocon beta subunit
GeneID:393153	NM_200184.1	ZDB-GENE-040426-848	serpine2	-0.7929651	0.03497039	serpin peptidase inhibitor%2C clade E (nexin%2C plasminogen activator inhibitor type 1)%2C member 2
GeneID:449775	NM_001005948.1	ZDB-GENE-041010-20	prrg1	1.01930339	0.03515847	proline rich Gla (G-carboxyglutamic acid) 1
GeneID:393805	NM_200832.1	ZDB-GENE-040426-1720	mid1ip1b	-1.0699754	0.03544527	MID1 interacting protein 1b
GeneID:751643	NM_001045362.1	ZDB-GENE-060825-91	krtcap2	-0.8263311	0.03544527	keratinocyte associated protein 2
GeneID:100008100	NM_001111254.2	ZDB-GENE-080206-1	zgc:174180	1.41854801	0.03550894	zgc:174180
GeneID:246094	NM_144763.1	ZDB-GENE-020513-2	gpia	-0.9157339	0.03565423	glucose-6-phosphate isomerase a
GeneID:559017	XM_009298437.3	ZDB-GENE-070912-575	arhgef4	-0.5035175	0.03571834	Rho guanine nucleotide exchange factor (GEF) 4%2C
GeneID:560006	NM_001145574.1	ZDB-GENE-090312-97	inpp4b	-0.8662666	0.03571834	inositol polyphosphate-4-phosphatase type II B%2C
GeneID:798632	NM_001005955.1	ZDB-GENE-041010-28	myl6	-0.6477322	0.03571834	myosin%2C light chain 6%2C alkali%2C smooth muscle and non-muscle%2C
GeneID:359833	NM_001309803.1	ZDB-GENE-030711-1	tfpia	0.94493374	0.03575301	tissue factor pathway inhibitor a%2C transcript variant 2
GeneID:100330405	XM_009292653.3	ZDB-GENE-080829-11	tmem176l.1	0.72935527	0.03597555	transmembrane protein 176l.1%2C
GeneID:393530	NM_001331200.1	ZDB-GENE-040426-1531	mybpha	-1.4575187	0.03620056	myosin binding protein Ha%2C transcript variant 1
GeneID:100149582	NM_001128395.1	ZDB-GENE-070705-193	si:ch211-71m22.1	0.87399301	0.03631413	si:ch211-71m22.1%2C
GeneID:322660	NM_199616.1	ZDB-GENE-030131-1380	upb1	-1.3351559	0.03631413	ureidopropionase%2C beta
GeneID:323368	NM_212639.1	ZDB-GENE-050522-2	ints10	-1.0022749	0.03631413	integrator complex subunit 10%2C
GeneID:378990	NM_001310061.1	ZDB-GENE-031010-33	nudt4a	0.75653993	0.03631413	nudix (nucleoside diphosphate linked moiety X)-type motif 4a%2C transcript variant 1
GeneID:445140	NM_001003534.1	ZDB-GENE-040801-41	mipa	1.07998685	0.03631413	major intrinsic protein of lens fiber a
GeneID:562146	XM_009304543.3			0.85915557	0.03636389	par-3 family cell polarity regulator beta b%2C
GeneID:797309	XM_001337745.7	ZDB-GENE-041210-324	cyp2j20	1.11368412	0.03636389	cytochrome P450%2C family 2%2C subfamily J%2C polypeptide 20
GeneID:798289	NM_001082923.2	ZDB-GENE-041210-237	pdzrn4	-1.1758765	0.03636389	PDZ domain containing ring finger 4
GeneID:100003049	XM_001342658.6	ZDB-GENE-100922-233	dus2	-0.7703122	0.03660515	dihydrouridine synthase 2%2C
GeneID:100038777	NM_001089557.2	ZDB-GENE-070424-101	zgc:163022	0.79072283	0.03660515	zgc:163022
GeneID:100149353	NM_001251831.1	ZDB-GENE-111212-1	trpm6	1.23009085	0.03660515	transient receptor potential cation channel%2C subfamily M%2C member 6%2C
GeneID:326962	NM_001305577.1	ZDB-GENE-030131-5162	cox5ab	-1.4014701	0.03660515	cytochrome c oxidase subunit Vab
GeneID:334932	NM_001328581.1	ZDB-GENE-030131-6872	mdm4	0.59074564	0.03660515	MDM4%2C p53 regulator%2C transcript variant 2
GeneID:393154	NM_200185.1	ZDB-GENE-040426-860	tuba8l4	-1.3072776	0.03660515	tubulin%2C alpha 8 like 4
GeneID:503753	NM_001013331.3	ZDB-GENE-050306-34	zgc:113263	0.69361226	0.03660515	zgc:113263%2C
GeneID:553655	NM_001020628.1	ZDB-GENE-050522-482	pygma	-1.3120092	0.03660515	phosphorylase%2C glycogen%2C muscle A
GeneID:565743	XM_005165378.4	ZDB-GENE-060216-4	pld2	0.65698227	0.03660515	phospholipase D2%2C
GeneID:568954	XM_009302576.3	ZDB-GENE-100729-4	dip2bb	0.69834414	0.03660515	disco-interacting protein 2 homolog Bb%2C
GeneID:798071	NM_213335.1	ZDB-GENE-040426-2649	sec13	-0.6639785	0.03660515	SEC13 homolog%2C nuclear pore and COPII coat complex component
GeneID:799619	XM_001921784.7	ZDB-GENE-081104-59	si:ch211-169p10.1	0.7631504	0.0366267	si:ch211-169p10.1%2C
GeneID:572234	NM_001201344.1	ZDB-GENE-110208-8	slc43a3a	0.89759539	0.03669623	solute carrier family 43%2C member 3a%2C
GeneID:100037324	NM_001089480.1	ZDB-GENE-070410-69	klhdc2	-1.2801487	0.03672767	kelch domain containing 2
GeneID:678595	NM_001040343.1	ZDB-GENE-060421-3368	tchp	-0.7546751	0.03678695	trichoplein%2C keratin filament binding
GeneID:100321338	XM_002662528.5	ZDB-GENE-090915-1	slc2a1b	0.86884677	0.0368131	solute carrier family 2 (facilitated glucose transporter)%2C member 1b
GeneID:100334524	XM_009303490.3			0.91783747	0.0368131	ring finger protein 166
GeneID:101886789	XM_005159753.4	ZDB-GENE-120215-196	susd5	1.01140181	0.0368131	sushi domain containing 5%2C
GeneID:108182240	XM_017354431.2			-1.2229916	0.0368131	cilia and flagella associated protein 44
GeneID:393535	NM_200563.3	ZDB-GENE-040426-1474	chmp1a	-0.8939991	0.0368131	charged multivesicular body protein 1A%2C
GeneID:394102	NM_201127.1	ZDB-GENE-040426-1426	surf4	-1.0825035	0.0368131	surfeit gene 4
GeneID:58023	NM_131511.1	ZDB-GENE-000906-4	fzd9b	-1.060312	0.0368131	frizzled class receptor 9b
GeneID:30477	NM_131230.1	ZDB-GENE-990714-24	tcp1	-1.1027261	0.03684222	t-complex 1
GeneID:541556	NM_001014370.1	ZDB-GENE-050327-97	rbm39b	-0.5099648	0.03684222	RNA binding motif protein 39b%2C
GeneID:571909	NM_001145700.1	ZDB-GENE-030131-8003	atl1	-0.8822229	0.03688639	atlastin GTPase 1%2C
GeneID:100004582	XM_001343807.8			0.82948319	0.03707085	uncharacterized LOC100004582
GeneID:100037318	NM_001089474.1	ZDB-GENE-070410-38	cerkl	-0.7613872	0.03710139	ceramide kinase-like
GeneID:555303	XM_677774.9			1.58106422	0.03710139	si:dkeyp-118h9.7
GeneID:562734	NM_001044994.2	ZDB-GENE-030131-5627	fam210aa	0.90754329	0.03710139	family with sequence similarity 210%2C member Aa
GeneID:557850	NM_001042730.1	ZDB-GENE-030912-7	trpv4	0.78076657	0.03728101	transient receptor potential cation channel%2C subfamily V%2C member 4%2C
GeneID:795027	XM_005155917.1	ZDB-GENE-030131-247	si:ch211-222l21.1	-0.6350798	0.03728101	si:ch211-222l21.1
GeneID:795950	NM_001102674.1	ZDB-GENE-050506-83	cyb5d2	-0.7243131	0.03728101	cytochrome b5 domain containing 2
GeneID:100537224	XM_021468539.1	ZDB-GENE-130531-41	adgrf8	1.0976114	0.03729177	adhesion G protein-coupled receptor F8
GeneID:100332905	XM_002662558.6	ZDB-GENE-120919-5	snta1	1.17326664	0.03735678	syntrophin%2C alpha 1
GeneID:100334776	NM_001347685.1			-1.0361456	0.03735678	phosphatase and actin regulator 1
GeneID:386663	NM_198816.1	ZDB-GENE-031031-4	pfkfb4b	0.90111581	0.03735678	6-phosphofructo-2-kinase/fructose-2%2C6-biphosphatase 4b%2C
GeneID:555376	NM_001045844.1	ZDB-GENE-060825-186	btd	-0.8786091	0.03735678	biotinidase
GeneID:798716	NM_001077802.1	ZDB-GENE-061110-16	ispd	-0.7683854	0.03735678	isoprenoid synthase domain containing
GeneID:799552	NM_001135136.1	ZDB-GENE-131121-645	si:ch211-157b11.14	-1.0378362	0.03735678	si:ch211-157b11.14
GeneID:100005026	XM_001344147.7	ZDB-GENE-100408-4	rilp	0.76921259	0.03751528	Rab interacting lysosomal protein%2C
GeneID:108180723	XM_017352917.2			0.92309204	0.03751528	probable E3 ubiquitin-protein ligase HECTD4
GeneID:553678	NM_001020651.1	ZDB-GENE-050522-237	zgc:112102	-1.6342211	0.03751528	zgc:112102
GeneID:560602	XM_684000.9	ZDB-GENE-081104-301	slc2a6	1.26451302	0.03754698	solute carrier family 2 (facilitated glucose transporter)%2C member 6
GeneID:100000160	NM_131430.1	ZDB-GENE-980526-488	fgfr4	-0.7048834	0.03766556	fibroblast growth factor receptor 4
GeneID:100270734	XM_003198619.5	ZDB-GENE-060526-379	sgsm1a	0.79517776	0.03766556	small G protein signaling modulator 1a%2C
GeneID:100536175	XM_003200201.5			-0.8170966	0.03766556	cortexin-2-like
GeneID:322880	NM_212630.1	ZDB-GENE-030131-1600	ewsr1b	-0.6539192	0.03766556	EWS RNA-binding protein 1b
GeneID:393302	NM_200331.1	ZDB-GENE-040426-1078	zgc:56556	-0.5153445	0.03766556	zgc:56556
GeneID:58022	NM_131510.2	ZDB-GENE-000713-1	casp8	0.61313328	0.03766556	caspase 8%2C apoptosis-related cysteine peptidase%2C
GeneID:796537	NM_131472.1	ZDB-GENE-000705-1	kdrl	0.60444941	0.037817	kinase insert domain receptor like%2C
GeneID:556236	XM_021476432.1	ZDB-GENE-060526-132	acacb	1.03065726	0.03783108	acetyl-CoA carboxylase beta%2C
GeneID:100149268	NM_001024461.3	ZDB-GENE-041008-1	polr2b	-0.9326811	0.03814356	polymerase (RNA) II (DNA directed) polypeptide B
GeneID:192311	NM_173238.2	ZDB-GENE-020419-13	srrt	-1.0210566	0.03814356	serrate RNA effector molecule homolog (Arabidopsis)
GeneID:322258	NM_212613.1	ZDB-GENE-030131-977	cct5	-0.9558952	0.03814356	chaperonin containing TCP1%2C subunit 5 (epsilon)
GeneID:324855	NM_212690.1	ZDB-GENE-030826-13	plaa	-0.9044862	0.03814356	phospholipase A2-activating protein
GeneID:402809	NM_205575.3	ZDB-GENE-040718-80	tnni2a.3	-1.378824	0.03814356	troponin I type 2a (skeletal%2C fast)%2C tandem duplicate 3
GeneID:550423	NM_001017728.2	ZDB-GENE-050417-234	f3b	-1.0849757	0.03814356	coagulation factor IIIb
GeneID:555681	NM_001137657.1	ZDB-GENE-081119-3	frem2a	0.96996569	0.03814356	Fras1 related extracellular matrix protein 2a%2C
GeneID:792197	NM_001001825.2	ZDB-GENE-040426-21	bgnb	1.46817489	0.03814356	biglycan b
GeneID:559958	NM_001351715.1	ZDB-GENE-170609-2	cdaa	0.83649521	0.03814507	cytidine deaminase a
GeneID:563854	NM_001045031.1	ZDB-GENE-030131-1030	si:ch211-154o6.3	-0.6762555	0.03814507	si:ch211-154o6.3
GeneID:100005140	NM_001113657.1	ZDB-GENE-080220-36	fbxo40.1	-1.874289	0.03836789	F-box protein 40%2C tandem duplicate 1
GeneID:100137105	NM_001114702.1	ZDB-GENE-080212-8	zgc:174710	1.29758322	0.03836789	zgc:174710
GeneID:565335	NM_001128344.1	ZDB-GENE-060526-35	slc25a15b	0.50820031	0.03853723	solute carrier family 25 (mitochondrial carrier%3B ornithine transporter) member 15b%2C
GeneID:334098	NM_212720.2	ZDB-GENE-030131-6030	hsd11b2	0.83106457	0.03854871	hydroxysteroid (11-beta) dehydrogenase 2
GeneID:140615	NM_131645.2	ZDB-GENE-011212-1	fads2	-1.0785039	0.03863626	fatty acid desaturase 2
GeneID:415253	NM_001002163.1	ZDB-GENE-040625-180	zgc:86599	-0.9349585	0.03869914	zgc:86599
GeneID:799716	NM_001007296.1	ZDB-GENE-041114-5	smarcb1a	-0.8406483	0.03887105	SWI/SNF related%2C matrix associated%2C actin dependent regulator of chromatin%2C subfamily b%2C member 1a%2C
GeneID:100331800	XM_005158862.4	ZDB-GENE-130530-548	zmp:0000000545	0.85913461	0.03887387	zmp:0000000545%2C
GeneID:321636	NM_199556.1	ZDB-GENE-030131-355	rprd1b	-0.5595835	0.03887387	regulation of nuclear pre-mRNA domain containing 1B%2C
GeneID:557341	NM_001082812.2	ZDB-GENE-060526-126	cmklr1	1.1357681	0.03887387	chemokine-like receptor 1
GeneID:799963	NM_001098757.2	ZDB-GENE-030729-8	gpt2	0.82738845	0.03887387	glutamic pyruvate transaminase (alanine aminotransferase) 2
GeneID:337851	NM_200074.3	ZDB-GENE-040407-1	cherp	-0.6619649	0.03898265	calcium homeostasis endoplasmic reticulum protein
GeneID:558312	NM_001100436.2	ZDB-GENE-070327-2	rplp2l	-1.6884712	0.03898265	ribosomal protein%2C large P2%2C like
GeneID:793927	XM_001333745.7	ZDB-GENE-090908-1	atxn1l	0.60493205	0.03906126	ataxin 1-like
GeneID:393555	NM_200583.1	ZDB-GENE-040426-1421	cct4	-1.2766732	0.03921009	chaperonin containing TCP1%2C subunit 4 (delta)
GeneID:561353	XM_005170201.4	ZDB-GENE-041014-293	efr3bb	0.63407945	0.03921009	EFR3 homolog Bb (S. cerevisiae)%2C
GeneID:406292	NM_213019.1	ZDB-GENE-040426-1955	ppib	-0.9276494	0.03960472	peptidylprolyl isomerase B (cyclophilin B)

GeneID:767648	NM_001076588.2	ZDB-GENE-060929-204	spata5l1	-1.289289	0.03961608	spermatogenesis associated 5-like 1
GeneID:573364	NM_214727.1	ZDB-GENE-040628-4	bsx	-0.7866988	0.03970801	brain-specific homeobox
GeneID:100331003	XM_005168460.4			0.76919616	0.03978324	zinc finger protein 583-like
GeneID:100334475	XM_002661160.6	ZDB-GENE-050302-128	cog1	-0.7906567	0.03978324	component of oligomeric golgi complex 1
GeneID:100008101	NM_001113484.1	ZDB-GENE-990415-184	nr4a2a	-0.7670973	0.03978899	nuclear receptor subfamily 4%2C group A%2C member 2a%2C
GeneID:171585	NM_001310044.1	ZDB-GENE-020326-1	psma6a	-0.7153706	0.03980543	proteasome subunit alpha 6a%2C transcript variant 1
GeneID:793301	NM_198064.1	ZDB-GENE-030131-4661	slc15a1b	0.91178765	0.03980543	solute carrier family 15 (oligopeptide transporter)%2C member 1b
GeneID:108183709	XM_017355632.2			1.05985665	0.03989236	gastrula zinc finger protein XlCGF8.2DB-like
GeneID:447833	NM_001004572.2	ZDB-GENE-040912-99	hat1	-0.6324811	0.03989236	histone acetyltransferase 1
GeneID:558271	NM_001076560.1	ZDB-GENE-031009-1	fscn1a	-0.6778009	0.03989236	fascin actin-bundling protein 1a
GeneID:794169	XM_003200655.5	ZDB-GENE-060810-114	ndufaf4	-1.0909662	0.03989236	NADH dehydrogenase (ubiquinone) complex I%2C assembly factor 4
GeneID:101883546	XM_005170635.4			-0.7953787	0.03992904	uncharacterized LOC101883546
GeneID:393920	NM_200946.1	ZDB-GENE-040426-694	crnkl1	-0.8131087	0.0401758	crooked neck pre-mRNA splicing factor 1
GeneID:403307	NM_203479.1	ZDB-GENE-040310-4	rtn4rl2a	-0.9979638	0.0401758	reticulon 4 receptor-like 2 a
GeneID:541507	NM_001014320.2	ZDB-GENE-050327-33	ppp1r13l	0.64694538	0.0401758	protein phosphatase 1%2C regulatory subunit 13 like%2C
GeneID:562834	NM_001045001.1	ZDB-GENE-060503-425	slc12a10.2	0.87449622	0.04020999	solute carrier family 12 (sodium/potassium/chloride transporters)%2C member 10%2C tandem duplicate 2
GeneID:100535682	XM_003199037.5	ZDB-GENE-081104-335	si:dkey-178e17.3	0.90557854	0.0404984	si:dkey-178e17.3%2C
GeneID:399483	NM_001002040.1	ZDB-GENE-040123-1	cdkn1ca	-0.9076528	0.0404984	cyclin-dependent kinase inhibitor 1Ca
GeneID:504111	NM_001328215.1	ZDB-GENE-050309-241	znf1035	0.73958698	0.0404984	zinc finger protein 1035%2C
GeneID:561971	NM_001083835.1	ZDB-GENE-070209-238	vcam1b	-0.7776788	0.0404984	vascular cell adhesion molecule 1b%2C
GeneID:378728	NM_200427.2	ZDB-GENE-030922-1	siah2l	0.78118245	0.04049975	seven in absentia homolog 2 (Drosophila)-like
GeneID:564573	XM_001920252.7	ZDB-GENE-070706-2	myo3b	-0.5784658	0.04049975	myosin IIIB%2C
GeneID:541347	NM_001013474.2	ZDB-GENE-030131-786	mat2b	-0.7945396	0.0405793	methionine adenosyltransferase II%2C beta%2C
GeneID:797938	NM_001111226.1	ZDB-GENE-080204-119	hbegfa	0.6573076	0.04065436	heparin-binding EGF-like growth factor a
GeneID:359827	NM_001007197.1	ZDB-GENE-030616-127	ier5	-0.7107067	0.04076419	immediate early response 5
GeneID:100002627	XM_001342336.5	ZDB-GENE-131127-236	ptafr	0.75423177	0.0409095	platelet-activating factor receptor
GeneID:321247	NM_001305468.1	ZDB-GENE-030131-9828	ftsj3	-0.9176114	0.0409095	FtsJ homolog 3 (E. coli)
GeneID:557878	NM_001159666.1	ZDB-GENE-070912-243	igsf3	-0.9508306	0.0409095	immunoglobulin superfamily%2C member 3
GeneID:569049	NM_001039823.1	ZDB-GENE-060312-33	cog4	-0.7330409	0.0409095	component of oligomeric golgi complex 4
GeneID:799904	NM_001083874.1	ZDB-GENE-141222-90	si:ch73-359m17.2	-2.3469086	0.0409095	si:ch73-359m17.2%2C
GeneID:100331484	NM_001351705.1	ZDB-GENE-030131-8244	ier2b	-0.9067587	0.04115519	immediate early response 2-like
GeneID:368704	NM_214722.1	ZDB-GENE-030619-9	ctsc	0.7271775	0.04132016	cathepsin C
GeneID:280650	NM_201461.2	ZDB-GENE-021115-9	klf6a	-0.4526597	0.04136893	Kruppel-like factor 6a
GeneID:555217	NM_212650.2	ZDB-GENE-030131-5055	zbtb11	-0.7299778	0.04138876	zinc finger and BTB domain containing 11
GeneID:110438482	XM_021470989.1			1.21846092	0.04157382	uncharacterized LOC110438482
GeneID:322275	NM_199595.1	ZDB-GENE-030131-994	rad21a	-0.4632868	0.04157382	RAD21 cohesin complex component a
GeneID:751764	NM_001045473.1	ZDB-GENE-041210-319	bmf2	0.84682329	0.04168752	Bcl2 modifying factor 2%2C
GeneID:325423	NM_001017860.1	ZDB-GENE-030131-4148	fstl1a	0.97555678	0.04168947	follistatin-like 1a
GeneID:393506	NM_200534.1	ZDB-GENE-040426-1417	wdr77	-1.0118457	0.04181888	WD repeat domain 77
GeneID:406782	NM_213473.1	ZDB-GENE-040426-2835	mapk4	0.68342491	0.04185168	mitogen-activated protein kinase 4%2C
GeneID:335499	NM_199954.1	ZDB-GENE-030131-7439	klc1b	-0.7576466	0.04186996	kinesin light chain 1b
GeneID:336303	NM_200008.1	ZDB-GENE-030131-8247	ppdpfb	-0.8886586	0.04186996	pancreatic progenitor cell differentiation and proliferation factor b
GeneID:394092	NM_201117.1	ZDB-GENE-040426-1316	nudt21	-0.5616064	0.04186996	nudix hydrolase 21
GeneID:402899	NM_205606.1	ZDB-GENE-040426-58	tada1	-0.8820241	0.04186996	transcriptional adaptor 1
GeneID:497403	XM_002665215.6	ZDB-GENE-041111-205	cenpf	1.24096136	0.04186996	centromere protein F%2C
GeneID:553611	NM_001020585.1	ZDB-GENE-050522-308	six6b	-0.7486616	0.04186996	SIX homeobox 6b
GeneID:558831	NM_001111159.1	ZDB-GENE-030131-4471	mprip	-0.5575989	0.04186996	myosin phosphatase Rho interacting protein%2C
GeneID:561047	NM_001025511.1	ZDB-GENE-040724-185	and3	-1.4958367	0.04186996	actinodin3
GeneID:791524	NM_001001949.3	ZDB-GENE-030131-8434	grna	1.28557748	0.04186996	granulin a%2C
GeneID:336823	NM_200038.1	ZDB-GENE-030131-8767	drg1	-0.7425293	0.04191024	developmentally regulated GTP binding protein 1
GeneID:553264	NM_001099238.1	ZDB-GENE-070112-1662	zgc:158564	0.71214447	0.04191024	zgc:158564
GeneID:568862	NM_001100037.1	ZDB-GENE-070705-229	si:ch73-266o15.4	0.92374566	0.04191024	si:ch73-266o15.4%2C
GeneID:393589	NM_200617.1	ZDB-GENE-040426-1244	rars2	-1.6801412	0.04223578	arginyl-tRNA synthetase 2%2C mitochondrial (putative)%2C
GeneID:564082	NM_001079680.1			-0.6059578	0.04223578	sperm autoantigenic protein 17
GeneID:558214	XM_005171249.4	ZDB-GENE-070912-696	slc12a7a	0.78122419	0.04223609	solute carrier family 12 (potassium/chloride transporter)%2C member 7a%2C
GeneID:103909514	XM_009295902.2	ZDB-GENE-121214-41	si:ch73-221f6.1	-0.726168	0.04229305	si:ch73-221f6.1
GeneID:406493	NM_213212.1	ZDB-GENE-040426-2296	myl9b	0.98662483	0.04239903	myosin%2C light chain 9b%2C regulatory
GeneID:259256	NM_201457.1	ZDB-GENE-020809-3	rfc3	-0.8194644	0.04248329	replication factor C (activator 1) 3
GeneID:393504	NM_200532.1	ZDB-GENE-040426-1473	rasd1	-1.0403378	0.04248329	RAS%2C dexamethasone-induced 1
GeneID:560245	XM_005158068.4	ZDB-GENE-030131-1361	si:ch211-197h24.6	-0.5677732	0.04249956	si:ch211-197h24.6%2C
GeneID:100004427	XM_005163898.3			1.60491121	0.04255998	serine protease 27-like
GeneID:100535421	XM_009292506.3			-0.804849	0.04255998	si:dkey-34d22.1
GeneID:559276	XM_005173182.4	ZDB-GENE-030131-8719	ahnak	0.80249431	0.04255998	AHNAK nucleoprotein%2C
GeneID:568202	NM_001034982.1	ZDB-GENE-050809-121	pora	0.46515979	0.04255998	P450 (cytochrome) oxidoreductase a
GeneID:572028	XM_009304392.3			1.08183149	0.04259712	si:dkey-11f4.14%2C
GeneID:368229	NM_200084.1	ZDB-GENE-030722-6	pmm2	0.89310014	0.04264777	phosphomannomutase 2
GeneID:100037309	NM_001089466.1	ZDB-GENE-070410-8	srebf2	-0.6492379	0.04275055	sterol regulatory element binding transcription factor 2
GeneID:101883541	XM_009304026.3			-1.2301113	0.04279342	serine/threonine-protein kinase pim-1-like%2C
GeneID:450021	NM_001006042.1	ZDB-GENE-041010-138	fip1l1b	-0.8709157	0.04279342	FIP1 like 1b (S. cerevisiae)%2C
GeneID:100333801	XM_005171722.4			1.1077616	0.04281007	myotubularin-related protein 3-like%2C
GeneID:170446	NM_131893.2	ZDB-GENE-020122-1	meis1b	-0.5624787	0.04281007	Meis homeobox 1 b
GeneID:445197	NM_001003591.1	ZDB-GENE-040801-110	fam219aa	1.19850251	0.04281007	family with sequence similarity 219%2C member Aa%2C
GeneID:664769	NM_001039995.1	ZDB-GENE-060312-46	slc35b3	-0.7547134	0.04281007	solute carrier family 35 (adenosine 3'-phospho 5'-phosphosulfate transporter)%2C member B3
GeneID:327462	NM_199809.1	ZDB-GENE-030131-5673	hsd3b7	0.71398233	0.04292986	hydroxy-delta-5-steroid dehydrogenase%2C 3 beta- and steroid delta-isomerase%2C
GeneID:386769	NM_001018133.2	ZDB-GENE-031113-24	ptprma	-1.1470479	0.04292986	protein tyrosine phosphatase%2C receptor type%2C M%2C a
GeneID:557840	NM_001044843.1	ZDB-GENE-050208-755	yjefn3	-0.8130777	0.04331195	YjeF N-terminal domain containing 3
GeneID:335206	NM_199939.1	ZDB-GENE-030131-7146	arrdc2	0.78195683	0.04350406	arrestin domain containing 2%2C
GeneID:100321249	XM_002663198.5	ZDB-GENE-090521-3	slc23a2	1.4894018	0.04350475	solute carrier family 23 (ascorbic acid transporter)%2C member 2%2C
GeneID:100000079	XM_001921984.6	ZDB-GENE-110718-2	soul5	-1.0167883	0.04357224	heme-binding protein soul5
GeneID:436866	NM_001002593.2	ZDB-GENE-040718-335	tnfsf10	-0.6853715	0.04357224	TNF superfamily member 10%2C transcript variant 1
GeneID:751725	NM_001045435.1	ZDB-GENE-031001-1	hspa12a	0.74517866	0.04357224	heat shock protein 12A%2C
GeneID:393676	NM_200703.2	ZDB-GENE-040426-1659	gorasp2	-0.6953948	0.04367926	golgi reassembly stacking protein 2%2C
GeneID:561969	NM_001128711.1	ZDB-GENE-041001-183	gpr176	-0.7263427	0.04370012	G protein-coupled receptor 176
GeneID:568430	NM_001144049.1	ZDB-GENE-041210-264	gpr37b	-1.1035104	0.04396274	G protein-coupled receptor 37b
GeneID:571044	XM_001922037.5	ZDB-GENE-110408-61	slc25a22a	0.8241017	0.04396274	si:dkey-201c1.2
GeneID:795295	XM_005165199.4	ZDB-GENE-060526-127	si:ch211-240b21.4	1.22946062	0.04396274	si:ch211-240b21.4
GeneID:555401	NM_001077598.1	ZDB-GENE-061103-112	aoc1	-0.6680688	0.04406538	amine oxidase%2C copper containing 1
GeneID:282554	NM_183067.1	ZDB-GENE-021119-1	drd3	-0.6431442	0.04423571	dopamine receptor D3%2C
GeneID:336610	NM_212757.2	ZDB-GENE-030131-8554	ywhaz	-0.6608318	0.04423571	tyrosine 3-monooxygenase/tryptophan 5-monooxygenase activation protein%2C zeta polypeptide
GeneID:503939	NM_001110390.1	ZDB-GENE-080204-25	senp6a	-0.8738036	0.04423571	SUMO1/sentrin specific peptidase 6a%2C
GeneID:767721	NM_001076659.1	ZDB-GENE-060929-1014	uhmk1	0.77951368	0.04423571	U2AF homology motif (UHM) kinase 1
GeneID:494176	NM_001009912.1	ZDB-GENE-040801-48	rdh12l	0.61782838	0.04452464	retinol dehydrogenase 12%2C like
GeneID:393723	NM_200750.1	ZDB-GENE-040426-1716	rps19	-1.5183361	0.04465172	ribosomal protein S19
GeneID:393840	NM_200866.1	ZDB-GENE-040426-1850	ctnnbl1	-0.6748106	0.04465172	catenin%2C beta like 1
GeneID:553318	NM_001256242.1	ZDB-GENE-040914-80	gtf3c3	-0.8473257	0.04465172	general transcription factor IIIC%2C polypeptide 3
GeneID:554967	NM_201184.2	ZDB-GENE-040426-810	psmd1	-0.9859126	0.04465172	proteasome 26S subunit%2C non-ATPase 1
GeneID:436656	NM_001002383.1	ZDB-GENE-040718-78	krt97	-1.3091789	0.04482333	keratin 97
GeneID:30343	NM_131120.1	ZDB-GENE-990415-108	hoxb8a	-1.3324154	0.04502066	homeobox B8a%2C
GeneID:373866	NM_194406.1	ZDB-GENE-030902-2	cbr1	0.85879641	0.04502066	carbonyl reductase 1%2C
GeneID:561773	NM_001025518.1	ZDB-GENE-040724-204	hrh3	-0.7796991	0.04502066	histamine receptor H3
GeneID:567964	XM_017355939.2	ZDB-GENE-070424-110	si:ch211-114l13.10	-0.8927085	0.04502066	si:ch211-114l13.10
GeneID:394176	NM_001277119.2	ZDB-GENE-030131-2074	pum1	-0.8659582	0.04505851	pumilio RNA-binding family member 1
GeneID:492472	NM_001007344.2	ZDB-GENE-041114-32	slc7a6os	-0.7324977	0.04505851	solute carrier family 7%2C member 6 opposite strand

GeneID:565099	NM_001128719.1	ZDB-GENE-080723-11	zgc:193725	1.7714663	0.04522821	zgc:193725
GeneID:565876	NM_001123277.1	ZDB-GENE-081105-124	si:dkey-91i10.3	0.87028036	0.04532647	si:dkey-91i10.3
GeneID:436882	NM_001002609.1	ZDB-GENE-040718-353	psmb2	-0.9141758	0.04546608	proteasome subunit beta 2
GeneID:568726	NM_001166272.2	ZDB-GENE-040713-1	adcy7	-0.732621	0.04546803	adenylate cyclase 7%2C
GeneID:100333310	XM_017354458.2	ZDB-GENE-150114-1	kdm5a	-0.7584417	0.04550393	lysine (K)-specific demethylase 5A
GeneID:553186	NM_001018132.1	ZDB-GENE-060118-2	barhl1b	-0.7418699	0.04555223	BarH-like homeobox 1b
GeneID:751746	NM_001045456.1	ZDB-GENE-060825-59	mrps6	-0.832404	0.0455739	mitochondrial ribosomal protein S6
GeneID:58092	NM_131571.2	ZDB-GENE-000509-3	smad3a	0.84279605	0.04580874	SMAD family member 3a
GeneID:100149386	NM_001128394.1	ZDB-GENE-060503-81	bnipl	0.9407586	0.04595654	BCL2/adenovirus E1B 19kD interacting protein%2C like%2C
GeneID:101886240	XM_005172833.4	ZDB-GENE-041210-266	crtc1b	0.8197224	0.04595654	CREB regulated transcription coactivator 1
GeneID:100003680	XM_001338982.7	ZDB-GENE-140106-164	pelp1	-0.630659	0.04605322	proline%2C glutamate and leucine rich protein 1
GeneID:556651	NM_001030103.1	ZDB-GENE-050913-50	nudt17	-0.6327952	0.04606772	nudix (nucleoside diphosphate linked moiety X)-type motif 17
GeneID:100037372	NM_001089524.1	ZDB-GENE-070410-70	cnot2	-0.4787379	0.046149	CCR4-NOT transcription complex%2C subunit 2%2C
GeneID:321556	NM_212589.2	ZDB-GENE-030131-275	hnrnph1l	-0.817604	0.04628321	heterogeneous nuclear ribonucleoprotein H1%2C like
GeneID:560799	XM_684199.7	ZDB-GENE-040724-117	rab44	1.12722341	0.04628321	RAB44%2C member RAS oncogene family
GeneID:324413	NM_212679.2	ZDB-GENE-030131-3133	bhlhe40	-0.9233247	0.04637625	basic helix-loop-helix family%2C member e40
GeneID:325550	NM_001328342.1	ZDB-GENE-030131-4275	ddx39aa	-0.8872879	0.04639791	DEAD (Asp-Glu-Ala-Asp) box polypeptide 39Aa
GeneID:557567	NM_001077146.2	ZDB-GENE-060929-328	bmp7b	-0.7481214	0.04661668	bone morphogenetic protein 7b%2C
GeneID:100034488	XM_005170088.4			0.76136601	0.04664563	Pim proto-oncogene%2C serine/threonine kinase%2C related 80%2C
GeneID:406600	NM_213308.1	ZDB-GENE-040426-2537	stk25b	-0.874656	0.04668245	serine/threonine kinase 25b%2C
GeneID:373114	NM_001313772.1	ZDB-GENE-030826-3	cflara	0.62238264	0.04672754	CASP8 and FADD-like apoptosis regulator a%2C
GeneID:100536673	XM_003200772.5	ZDB-GENE-050208-270	si:ch73-91k6.2	1.0499105	0.04686194	alpha-1%2C6-mannosyl-glycoprotein 2-beta-N-acetylglucosaminyltransferase-like
GeneID:266797	NM_201458.2	ZDB-GENE-021015-3	stt3a	-0.6904667	0.04686194	STT3A%2C subunit of the oligosaccharyltransferase complex (catalytic)
GeneID:30757	NM_131471.1	ZDB-GENE-990415-145	mhc1uba	1.32369424	0.04686194	major histocompatibility complex class I UBA
GeneID:564730	XM_021470025.1			0.75498615	0.04686194	P2Y purinoceptor 1-like
GeneID:100170811	NM_001130646.1	ZDB-GENE-060503-80	si:dkey-6n6.1	-1.2367327	0.04694128	si:dkey-6n6.1
GeneID:30190	NM_130997.2	ZDB-GENE-980526-283	egr2b	-0.9025976	0.04694128	early growth response 2b%2C
GeneID:405894	NM_212958.1	ZDB-GENE-040426-2427	prkar2ab	-1.1625799	0.04694128	protein kinase%2C cAMP-dependent%2C regulatory%2C type II%2C alpha%2C B
GeneID:572372	NM_001128766.1	ZDB-GENE-080723-77	gjd1a	-0.8832295	0.04694128	gap junction protein delta 1a
GeneID:497162	NM_001033093.1	ZDB-GENE-131121-640	si:ch211-5k11.8	-1.5789708	0.04694585	si:ch211-5k11.8
GeneID:503594	NM_001013282.2	ZDB-GENE-050227-5	il4r.1	0.8797968	0.04694615	interleukin 4 receptor%2C tandem duplicate 1%2C
GeneID:652982	XM_005162972.3			-0.6871959	0.04694615	iron-responsive element binding protein 2
GeneID:573111	NM_001033586.1	ZDB-GENE-041010-177	tpmt.2	0.92257066	0.04703893	thiopurine S-methyltransferase%2C tandem duplicate 2%2C
GeneID:368645	NM_001003574.1	ZDB-GENE-030616-546	tmem267	0.78958099	0.04705846	transmembrane protein 267%2C transcript variant 1
GeneID:393284	NM_200314.2	ZDB-GENE-040426-901	pdgfrl	-1.0861201	0.04705846	platelet-derived growth factor receptor-like
GeneID:110437722	XM_021471331.1			0.77010284	0.04716412	gastrula zinc finger protein XlCGF8.2DB-like%2C
GeneID:437015	NM_001002742.2	ZDB-GENE-040718-242	trappc1	-0.708146	0.04720242	trafficking protein particle complex 1%2C
GeneID:100148383	XM_001921546.7	ZDB-GENE-060418-2	sec31b	-0.7238972	0.04747135	SEC31 homolog B%2C COPII coat complex component%2C
GeneID:100142646	NM_001122622.1	ZDB-GENE-080303-29	zgc:174862	1.09117573	0.04752962	zgc:174862
GeneID:100332245	XM_009304979.3	ZDB-GENE-120313-6	cobll1b	0.60626169	0.04752962	cordon-bleu WH2 repeat protein-like 1b%2C
GeneID:100332553	XM_002661995.6	ZDB-GENE-060526-263	si:dkey-229d2.4	0.94923202	0.04752962	si:dkey-229d2.4
GeneID:100498671	XM_017354419.2	ZDB-GENE-100701-3	ccdc88ab	-1.1483258	0.04752962	coiled-coil domain containing 88Ab%2C
GeneID:100538081	XM_003199759.5			1.10094393	0.04752962	CDK2 associated cullin domain 1
GeneID:101886002	XM_017354471.2			-1.224776	0.04752962	intraflagellar transport protein 122 homolog
GeneID:393584	NM_200612.1	ZDB-GENE-040426-1208	mtmr4	0.58140596	0.04752962	myotubularin related protein 4%2C
GeneID:541384	NM_001013511.2	ZDB-GENE-050320-80	mybpc2b	-1.2605676	0.04752962	myosin binding protein C%2C fast type b%2C
GeneID:541552	NM_001014365.1	ZDB-GENE-050327-93	eya4	-0.8385846	0.04752962	EYA transcriptional coactivator and phosphatase 4%2C
GeneID:100332046	XM_009307655.3			0.70226936	0.04770941	TBC1 domain family member 9B%2C
GeneID:492334	NM_001007300.1	ZDB-GENE-041114-9	slc5a11	0.95533478	0.04770941	solute carrier family 5 (sodium/inositol cotransporter)%2C member 11
GeneID:100137113	NM_001114710.1	ZDB-GENE-080215-16	zgc:171474	-2.3111382	0.04783417	zgc:171474
GeneID:100333944	XM_002667372.5	ZDB-GENE-121003-4	psmg4	-1.2037822	0.04783417	proteasome (prosome%2C macropain) assembly chaperone 4
GeneID:30236	NM_131038.1	ZDB-GENE-991105-4	cx43	0.74347118	0.04783417	connexin 43
GeneID:550241	NM_001017579.1	ZDB-GENE-050417-38	rrp7a	-1.2970509	0.04783417	ribosomal RNA processing 7 homolog A
GeneID:569207	NM_001080193.1	ZDB-GENE-070112-2002	kdm6al	-0.9616632	0.04783417	lysine (K)-specific demethylase 6A%2C like%2C
GeneID:572187	NM_001110011.1	ZDB-GENE-080108-5	dock1	-0.7303158	0.04783417	dedicator of cytokinesis 1
GeneID:724017	NM_001045322.1	ZDB-GENE-060610-2	crb3a	1.01744626	0.04783417	crumbs homolog 3a
GeneID:751740	NM_001045450.1	ZDB-GENE-060825-43	anapc15	-1.0053218	0.04783417	anaphase promoting complex subunit 15
GeneID:83415	NM_131783.2	ZDB-GENE-010412-1	six2a	-0.9403625	0.04783417	SIX homeobox 2a
GeneID:569359	XM_692739.9			0.86986953	0.04809762	jade family PHD finger 2
GeneID:100331601	XM_002662799.6	ZDB-GENE-110304-4	peli3	-1.3511948	0.04820812	pellino E3 ubiquitin protein ligase family member 3
GeneID:100333901	XM_017354413.2			-1.2699983	0.04822916	phosphatidylinositol transfer protein membrane associated 2
GeneID:406387	NM_213113.2	ZDB-GENE-040426-2117	rpl26	-1.6392733	0.04822916	ribosomal protein L26
GeneID:445233	NM_001003627.2	ZDB-GENE-040801-147	me2	-0.6715698	0.04822916	malic enzyme 2%2C NAD(+)-dependent%2C mitochondrial
GeneID:445083	NM_001003477.1	ZDB-GENE-040801-217	dhrs3a	0.72463891	0.04823362	dehydrogenase/reductase (SDR family) member 3a
GeneID:557340	NM_001044826.1	ZDB-GENE-060503-759	atxn1a	0.57186608	0.04823362	ataxin 1a%2C
GeneID:569608	NM_001030237.1	ZDB-GENE-050913-70	slc25a29	1.19611694	0.04823362	solute carrier family 25 (mitochondrial carnitine/acylcarnitine carrier)%2C member 29
GeneID:406586	NM_001007187.1	ZDB-GENE-040426-2495	fam53b	-0.7198356	0.04858829	family with sequence similarity 53%2C member B%2C
GeneID:445085	NM_001003479.1	ZDB-GENE-040801-220	rasgef1bb	-0.6662838	0.04858829	RasGEF domain family%2C member 1Bb
GeneID:554130	NM_001024425.1	ZDB-GENE-050522-397	stx18	-0.8263174	0.04858829	syntaxin 18%2C
GeneID:555585	XM_021471425.1	ZDB-GENE-030131-5416	si:dkey-68o6.5	3.59588653	0.04858829	si:dkey-68o6.5%2C
GeneID:100535636	XM_009299406.3			0.7974956	0.04865911	protein shisa-9-like%2C
GeneID:327284	NM_001001817.1	ZDB-GENE-040718-159	lgals9l3	1.02932417	0.04865911	lectin%2C galactoside-binding%2C soluble%2C 9 (galectin 9)-like 3
GeneID:558291	NM_001082816.2	ZDB-GENE-030131-8089	hapln2	0.82655455	0.04865911	hyaluronan and proteoglycan link protein 2
GeneID:557266	XM_005157161.4	ZDB-GENE-141216-78	purab	0.82300135	0.04867177	purine-rich element binding protein Ab
GeneID:334582	NM_001001404.1	ZDB-GENE-030131-6514	vdac1	-0.6799324	0.0488565	voltage-dependent anion channel 1
GeneID:368235	NM_201316.1	ZDB-GENE-030425-2	yars	0.56564551	0.0488565	tyrosyl-tRNA synthetase%2C
GeneID:437027	NM_001002754.2	ZDB-GENE-040718-256	rac3a	-0.7896346	0.0488565	ras-related C3 botulinum toxin substrate 3a (rho family%2C small GTP binding protein Rac3)
GeneID:570100	XM_021467785.1	ZDB-GENE-120221-6	admb	-1.0803974	0.0488565	adrenomedullin b%2C
GeneID:767644	NM_001076584.1	ZDB-GENE-060929-168	kat7a	-0.8504732	0.0488565	K(lysine) acetyltransferase 7a
GeneID:101883673	XM_005172323.4	ZDB-GENE-160728-24	si:dkey-183c6.9	-0.8836869	0.04896718	si:dkey-183c6.9
GeneID:30138	NM_130955.2	ZDB-GENE-990415-47	dld	-0.7045859	0.04896718	deltaD
GeneID:321948	NM_001024817.1	ZDB-GENE-030131-667	ddx46	-1.107021	0.04896718	DEAD (Asp-Glu-Ala-Asp) box polypeptide 46%2C
GeneID:555274	NM_001034174.1	ZDB-GENE-050809-126	gpr157	0.66883114	0.04896718	G protein-coupled receptor 157
GeneID:556575	NM_001135971.1	ZDB-GENE-081022-106	lratb.2	-0.8216142	0.04896718	lecithin retinol acyltransferase b%2C tandem duplicate 2
GeneID:798757	NM_001077161.1	ZDB-GENE-060929-868	znf438	0.72477057	0.04896718	zinc finger protein 438%2C
GeneID:567916	XM_009306797.3	ZDB-GENE-110420-5	stxbp4	0.64240403	0.04904463	syntaxin binding protein 4%2C
GeneID:791206	NM_001080688.1	ZDB-GENE-070112-1892	pdk3b	1.00397175	0.04904463	pyruvate dehydrogenase kinase%2C isozyme 3b
GeneID:794752	NM_001105683.1	ZDB-GENE-040426-2128	ckmb	-1.3345194	0.04904463	creatine kinase%2C muscle b%2C
GeneID:563776	NM_001130779.1	ZDB-GENE-081022-163	deaf1	-0.5201524	0.04907103	DEAF1 transcription factor%2C
GeneID:555882	NM_001080560.2	ZDB-GENE-030131-866	fnta	-0.5189566	0.04908191	farnesyltransferase%2C CAAX box%2C alpha
GeneID:100003877	NM_001134684.1	ZDB-GENE-090821-1	lrp6	-1.5384025	0.04909896	low density lipoprotein receptor-related protein 6
GeneID:101882393	XM_017352053.2			-0.9730404	0.04909896	uncharacterized LOC101882393
GeneID:406485	NM_213204.1	ZDB-GENE-040426-2284	rps20	-1.4561971	0.04909896	ribosomal protein S20
GeneID:567024	NM_001031674.2	ZDB-GENE-060312-26	lgsn	-0.9092806	0.04911817	lengsin%2C lens protein with glutamine synthetase domain
GeneID:100006377	NM_001122946.1	ZDB-GENE-060531-45	senp7b	-0.6705961	0.04912153	SUMO1/sentrin specific peptidase 7b%2C
GeneID:368864	NM_001004520.1	ZDB-GENE-030616-420	prpf39	-0.9949465	0.04912153	PRP39 pre-mRNA processing factor 39 homolog (yeast)
GeneID:100535378	XM_003200909.5	ZDB-GENE-120215-136	si:ch73-269m14.2	0.56960266	0.04912311	si:ch73-269m14.2
GeneID:100536156	XM_017352387.2			-1.3175856	0.04926636	venom phosphodiesterase 1-like
GeneID:798916	NM_001100063.1	ZDB-GENE-060526-348	upk3b	1.12311737	0.04926636	uroplakin 3b
GeneID:336712	NM_200027.1	ZDB-GENE-030131-8656	rpl10	-1.3321399	0.04941511	ribosomal protein L10%2C
GeneID:65233	NM_131718.2	ZDB-GENE-010201-2	six4a	-1.5463653	0.04946091	SIX homeobox 4a
GeneID:393943	NM_200968.1	ZDB-GENE-040426-1124	ndufa6	-1.742832	0.0495708	NADH dehydrogenase (ubiquinone) 1 alpha subcomplex%2C 6
GeneID:100007749	NM_001172400.1	ZDB-GENE-070705-4	gphnb	-0.4711656	0.04958728	gephyrin b%2C
GeneID:100149189	NM_001113579.1	ZDB-GENE-081105-55	si:ch73-237c6.1	0.7668005	0.04958728	si:ch73-237c6.1%2C

GeneID:103909792	XM_017353896.2			-1.0869043	0.04958728	NEDD4-like E3 ubiquitin-protein ligase WWP1
GeneID:352920	NM_178291.2	ZDB-GENE-030407-1	oxt	-1.3558048	0.04958728	oxytocin
GeneID:352928	NM_001007282.2	ZDB-GENE-030410-2	gpx4a	-0.7134616	0.04958728	glutathione peroxidase 4a%2C transcript variant 1
GeneID:394165	NM_201190.1	ZDB-GENE-040426-720	nelfcd	-0.5452987	0.04958728	negative elongation factor complex member C/D
GeneID:406472	NM_213191.1	ZDB-GENE-040426-2255	nudcd3	-0.9210206	0.04958728	NudC domain containing 3
GeneID:436612	NM_001002340.1	ZDB-GENE-040718-29	cldn7a	0.81411269	0.04958728	claudin 7a
GeneID:445503	NM_001004007.2	ZDB-GENE-040822-21	matn3a	1.09572977	0.04958728	matrilin 3a%2C transcript variant 2
GeneID:449989	NM_001006010.1	ZDB-GENE-041010-102	sssca1	-0.7353973	0.04958728	Sjogren syndrome/scleroderma autoantigen 1
GeneID:541504	NM_001014317.2	ZDB-GENE-050327-30	igfn1.1	-1.1223911	0.04958728	immunoglobulin-like and fibronectin type III domain containing 1%2C tandem duplicate 1%2C
GeneID:550327	NM_001017634.1	ZDB-GENE-050417-108	pcyt1aa	0.6226935	0.04958728	phosphate cytidylyltransferase 1%2C choline%2C alpha a%2C
GeneID:556852	NM_001044354.2	ZDB-GENE-050420-389	irf2bp1	-0.6799541	0.04958728	interferon regulatory factor 2 binding protein 1
GeneID:561457	XM_005158613.4	ZDB-GENE-081028-33	si:ch211-236p5.2	0.69980933	0.04958728	si:ch211-236p5.2
GeneID:565829	NM_001102389.2	ZDB-GENE-030131-9045	emc10	-0.6815175	0.04958728	ER membrane protein complex subunit 10%2C transcript variant 1
GeneID:568039	NM_001080603.1	ZDB-GENE-070105-3	ttc7b	-0.5272649	0.04958728	tetratricopeptide repeat domain 7B%2C
GeneID:569876	NM_001100628.1	ZDB-GENE-030131-9839	rgs4	0.83702327	0.04958728	regulator of G protein signaling 4
GeneID:573346	NM_001045227.1	ZDB-GENE-010716-2	irx5a	-0.5614261	0.04958728	iroquois homeobox 5a%2C
GeneID:724003	NM_001045308.2	ZDB-GENE-060616-210	rsrp1	-1.4613307	0.04958728	arginine/serine-rich protein 1
GeneID:794293	NM_001109724.1	ZDB-GENE-080103-3	tead3b	1.07316275	0.04958728	TEA domain family member 3 b%2C
GeneID:567738	XM_691038.7	ZDB-GENE-100922-229	fbxo21	0.7234607	0.04966488	F-box protein 21
GeneID:100149283	XM_001922684.7	ZDB-GENE-120503-2	znf710b	-0.5017935	0.049684	zinc finger protein 710b%2C
GeneID:368435	XM_005173268.4			1.28653743	0.049684	pyrimidinergic receptor P2Y%2C G-protein coupled%2C 4%2C
GeneID:555377	NM_001044784.1	ZDB-GENE-070410-92	zgc:162780	-1.1982732	0.049684	zgc:162780
GeneID:558711	NM_001025502.1	ZDB-GENE-050913-114	h2afy2	-0.7658308	0.04983431	H2A histone family%2C member Y2
GeneID:335153	NM_207058.1	ZDB-GENE-030131-7093	setdb2	-0.8718137	0.04998023	SET domain%2C bifurcated 2
GeneID:571530	XM_695132.7	ZDB-GENE-131121-257	si:dkey-21a6.5	0.5727387	0.04998023	si:dkey-21a6.5

a) Only genes with FDR<0.05 are listed

Supplementary Table ST4. Functional analysis of over- and underexpressed genes in AA-treated samples relative to controls

Functional analysis of overexpressed genes in acrylamide treated brain samples

Term	Count	Fold Enrichment	PValue	FDR(%)	Genes
GO:0006355~regulation of transcription, DNA-templated	31	2.2	0.0000477	0.06	EPAS1A, ONECUT2, SI:CH211-142B24.6, NFE2L2A, TEAD3B, HOXD13A, HOXB5B, HOXB5A, SI:CH73-63E15.2, CREMA, HOXB9A, SH2D5, PPARDA, NR1D2A, HOXC4A, NFIL3-6, RARGA, SATB2, VDRB, TEFB, HOXA4A, CEBPD, TFE3A, MYCH, FOXG1B, CSDC2B, ATF3, SBNO2A, IRF7, AHRRA, IRF3
IPR018108:Mitochondrial substrate/solute carrier	7	10.6	0.0000505	0.07	SLC25A24L, UCP2, SLC25A1B, SLC25A29, SLC25A23A, SLC25A47A, SLC25A15A
GO:0003700~transcription factor activity, sequence-specific DNA binding	20	2.8	0.0000895	0.12	NFIL3-6, RARGA, EPAS1A, VDRB, TEFB, HOXA4A, CEBPD, NFE2L2A, MYCH, TEAD3B, HOXB5B, HOXB5A, FOXG1B, CREMA, ATF3, NR1D2A, PPARDA, IRF7, IRF3, HOXC4A
dre01100:Metabolic pathways	24	2.1	0.000148	0.16	FBP1A, GLULB, HYAL3, GGT5B, GPT2L, GGT5A, CBR1L, ALPI.2, ACACB, LPIN1, MTHFD1L, CMBL, ALDH3A1, FAH, ITPKCB, ASPA, COQ2, CYP2J20, HPDB, HSD17B3, AOC2, OAT, PCYT1BB, MAT2AB
IPR017995:Homeobox protein, antennapedia type	4	33.8	0.000192	0.27	HOXA4A, HOXB5B, HOXC4A, HOXB5A
Symport	6	9.9	0.000342	0.38	SLC1A4, SLC1A5, SLC6A5, SLC6A16B, SLC6A15, SLC20A1A
GO:0050727~regulation of inflammatory response	4	24.9	0.000493	0.65	SI:CH73-63E15.2, SBNO2A, PYCARD, CASPB
GO:0006810~transport	23	1.9	0.00388109	4.99	SYPL2B, SLC25A1B, SLC6A16B, SLC6A15, SLC4A4B, SLC25A23A, BICD2, SLC19A2, SLC20A1A, SLC1A4, SLC1A5, SLC2A6, SLC25A24L, CHCHD4A, UCP2, SLC23A2, SLC6A5, APOEB, SLC25A29, SLC2A15B, SLC25A47A, SLC25A15A, MIPA

Functional analysis of underexpressed genes in acrylamide treated brain samples

Term	Count	Fold Enrichment	PValue	FDR	Genes
IPR000217:Tubulin	15	16.1	4E-14	0	TUBB2B, TUBA8L2, TUBA8L3, TUBA8L4, ZGC:153426, SI:CH73-199E17.1, TUBA2, SI:CH211-114N24.6, TUBB5, TUBG1, TUBA1A, TUBB2, TUBA1B, TUBA1C, TUBB4B
GO:0007017~microtubule-based process	17	11.5	2.39E-13	0	TUBB2B, TUBA8L2, TUBA8L3, TUBA8L4, DYNLL1, ZGC:153426, SI:CH73-199E17.1, DYNLL2B, TUBB5, TUBA2, SI:CH211-114N24.6, TUBG1, TUBA1A, TUBB2, TUBA1B, TUBA1C, TUBB4B
GO:0005200~structural constituent of cytoskeleton	15	8.4	1.38E-09	0	MYLIPA, TUBB2B, TUBA8L2, TUBA8L3, TUBA8L4, ZGC:153426, SI:CH73-199E17.1, TUBA2, SI:CH211-114N24.6, TUBB5, TUBA1A, TUBB2, TUBA1B, TUBA1C, TUBB4B
dre03040:Spliceosome	21	3.5	0.00000171	0	HNRNPA1B, CHERP, CCDC12, CRNKL1, HNRNPA1A, RBM25B, CDC5L, TCERG1A, SF3B2, CTNNBL1, HNRNPM, EIF4A3, DDX46, SRSF3B, RBM8A, SRSF10B, CDC40, SNRPB, SYF2, U2AF1, SNRPF
GO:0005874~microtubule	17	4	0.00000453	0.01	TUBB2B, MID1IP1B, TUBA8L2, TUBA8L3, TUBA8L4, ZGC:153426, SI:CH73-199E17.1, TUBB5, SI:CH211-114N24.6, TUBA2, CLASP2, TUBG1, TUBA1A, TUBB2, TUBA1B, TUBA1C, TUBB4B
GO:0003924~GTPase activity	19	3.7	0.0000042	0.01	TSR1, TUBB2B, ATL1, MTIF2, TUBA8L2, TUBA8L3, TUBA8L4, ZGC:153426, SI:CH73-199E17.1, TUBB5, TUBA2, SI:CH211-114N24.6, RALAA, TUBG1, TUBA1A, TUBB2, TUBA1B, TUBA1C, TUBB4B
Transcription	45	1.9	0.0000351	0.04	MEAF6, POU6F1, EPAS1B, SI:CH211-216L23.1, TADA1, FOXO4, POLR2B, STAT6, BSX, SRRT, RBB4L, MED28, ILF3B, EED, TFDP2, CREB3L2, HIF1AL, BHLHE40, INSM1A, NR4A2A, ETV4, EGR1, THRAA, SMAD3A, IRF2BP1, HER6, HER9, STAT3, SREBF2, HOXB8A, MED6, TBR1B, PCGF5B, FEZF2, EYA4, ILF2, ASCL1A, SOX21B, JUN, EGR2B, NEUROD2, SMARCB1A, CNOT11, NEUROD4, CARM1
GO:0005856~cytoskeleton	27	2.5	0.0000367	0.05	ZGC:101810, MYLIPA, TUBB2B, ACTB1, ACTB2, MID1IP1B, FSCN1A, FRMPD3, TUBA8L2, TUBA8L3, FRMD4BB, IFT122, TUBA8L4, ZGC:153426, ZGC:112102, SI:CH73-199E17.1, SI:CH211-114N24.6, TUBB5, TUBA2, EVPLB, TCHP, CLASP2, TUBA1A, TUBA1B, TUBB2, TUBA1C, TUBB4B
Activator	17	3.4	0.0000463	0.06	EGR1, MEAF6, THRAA, SI:CH211-216L23.1, STAT3, SREBF2, STAT6, MED6, BSX, SRRT, MED28, ILF2, EGR2B, CREB3L2, NEUROD2, SMARCB1A, ETV4
GTP-binding	22	2.7	0.0000754	0.1	TUBB2B, ATL1, ARL9, TUBA8L2, TUBA8L3, SI:DKEY-27J5.5, ARL5A, TUBA8L4, ZGC:153426, SI:CH73-199E17.1, TUBB5, SI:CH211-114N24.6, TUBA2, RALAA, TUBG1, TUBA1A, RHOAB, RASD1, TUBB2, TUBA1B, TUBA1C, TUBB4B
GO:0005515~protein binding	20	2.6	0.000222	0.33	HAPLN2, FGFR4, THRAA, MYLIPA, SMAD3A, ZGC:110372, ZGC:113263, CX43, MEIS1B, KDR, TMEM147, DLA, KDRL, RTN4RL2A, UBE2IB, PDGFRL, DLD, RTN4RL2B, VEGFAB, BOC
dre04145:Phagosome	18	2.6	0.00054	0.62	RILP, TUBB2B, ACTB1, ACTB2, TUBA8L2, TUBA8L3, TUBA8L4, SEC61B, ZGC:153426, STX18, TUBB5, TUBA2, DYNC1H1, TUBA1A, TUBB2, TUBA1B, TUBA1C, TUBB4B
GO:0006355~regulation of transcription, DNA-templated	73	1.5	0.000781	1.21	POU6F1, ELF3, NKX2.4A, SI:CH211-216L23.1, CNOT2, FOXO4, BSX, RBB4L, ILF3B, MED28, EED, CREB3L2, DLX2A, HIF1AL, INSM1A, NR4A2A, EGR1, NUP133, RBPJB, IRX1B, CREB5A, HER6, IRF2BP1, HER9, HOXB8A, TBR1B, MED6, HMGB2B, NAB1A, EYA4, SIX4A, JUN, MYRF, SMARCB1A, CNOT11, NUP107, CARM1, MEAF6, EPAS1B, IRX5A, ZBTB11, TADA1, BARHL1B, STAT6, SRRT, TFDP2, MYCBP, BHLHE40, KAT7A, IRX1A, ETV4, THRAA, BHLHE22, SMAD3A, TFE3B, MEIS1B, FOXA1, SIX2A, ISL1, SIX6B, STAT3, SREBF2, FOXG1A, PCGF5B, FEZF2, ASCL1A, ILF2, SOX21B, EGR2B, NEUROD2, NEUROD4, DLX5A, TP53INP1
dre04540:Gap junction	15	2.6	0.001813	2.06	TUBB2B, ADCY7, CX43, TUBA8L2, TUBA8L3, TUBA8L4, ZGC:153426, RAF1B, TUBA2, TUBB5, TUBA1A, TUBB2, TUBA1B, TUBA1C, TUBB4B
dre03013:RNA transport	17	2.4	0.00184911	2.1	NUP133, ELAC1, STRAP, EIF5, EIF3EB, EIF4A3, UBE2IB, EIF3HA, RBM8A, EIF4BB, NUP50, SEC13, NUP107, THOC5, NUP43, GEMIN7, KPNB1
Developmental protein	30	1.9	0.00173128	2.18	MYLIPA, CX43, SI:CH211-216L23.1, PPDPFB, APLNRB, KDRL, WNT7AA, IFT122, UBE2IB, CREB3L2, DLX2A, INSM1A, SETDB2, THRAA, RPL24, FZD7A, ISL1, FZD6, HOXB8A, KDR, FEZF2, WNT7BB, DLA, ASCL1A, DLD, FZD9B, NEUROD2, NEUROD4, WIF1, DLX5A
IPR017986:WD40-repeat-containing domain	22	2.1	0.00167474	2.68	STXBP5B, SEC31B, RBBP5, STRAP, UTP18, DDB1, KCTD3, WDR83, PLAA, CORO2A, IFT122, WDPCP, RBB4L, CDC40, WDR77, EED, SEC13, SI:CH211-154O6.3, SI:CH73-266O15.4, WDR1, NUP43, PPWD1
Differentiation	13	2.8	0.00240262	3.01	FEZF2, DLA, KDRL, ASCL1A, CX43, SI:CH211-216L23.1, DLD, NEUROD2, PPDPFB, NEUROD4, INSM1A, THOC5, KDR
IPR002194:Chaperonin TCP-1, conserved site	4	14.5	0.00201417	3.22	TCP1, CCT5, CCT4, CCT2
IPR020902:Actin/actin-like conserved site	5	8.5	0.00233995	3.73	ACTR3, ZGC:101810, ACTA2, ACTB1, ACTB2

Supplementary Table ST5. KEGG pathway results from the joint transcriptomic, proteomic, and metabolomic datasets[a]

Term	Description	Names
dre01100	Metabolic pathways	C00003, C00025, C00041, C00049, C00051, C00127, C00386, C00719, acacb, adcy7, adh5, agpat9l, ahcyl1, ak5, alas1, aldh2.2, aldh3a1, aldh9a1a.1, aldoab, aldoca, aldocb, alpi.2, aoc1, aoc2, aspa, b3galnt2, b3gat1a, btd, cbr1, cbr1l, cbsb, cdaa, chac1, ckmb, ckmt1, cmbl, coq2, cox5ab, cyp19a1b, enoph1, fads2, fah, fbp1a, gad1a, gad2, galnt11, galnt18b, gatm, ggt5a, ggt5b, glo1, glulb, gmds, gmpr2, gnsb, gphnb, gpia, gpt2, gpt2l, gpx4a, gsr, gstp1, gstp2, hmgcs1, hpdb, hsd11b2, hsd17b3, hsd17b4, hsd3b7, hyal3, inpp4b, itpkcb, lpin1, mat2ab, mat2b, mpi, mtap, mthfd1l, mthfd2, mtmr4, ndufa5, ndufa6, nme2b.1, oat, ogdha, pcca, pcyt1aa, pcyt1bb, pde4d, pgam1a, pgam2, pgap1, pip5k1ca, pld2, pmm2, pnpo, prdx6, ptges3a, pygma, smox, stt3a, tdh, upb1
dre04540	Gap junction	C00025, adcy7, cx43, gna11a, grb2a, grb2b, prkcbb, raf1b, tuba1a, tuba1b, tuba1c, tuba2, tuba8l2, tuba8l3, tuba8l4, tubb2, tubb2b, tubb4b, tubb5
dre04080	Neuroactive ligand-receptor interaction	C00025, C00049, aplnrb, chrna4b, crhr1, drd3, gabra1, glra2, grm2b, hrh3, htr1ab, oxt, ptafr, ptger2a, s1pr3a, sstr1a, thraa
dre04068	FoxO signaling pathway	C00025, cdkn1a, foxg1a, foxo4, grb2a, grb2b, homer2, irs2a, klf2a, prmt1, raf1b, sgk3, smad3a, sod2, stat3, tnfsf10
dre01200	Carbon metabolism	C00025, C00041, C00049, adh5, aldoab, aldoca, aldocb, fbp1a, gpia, gpt2, gpt2l, me2, ogdha, pcca, pgam1a, pgam2
dre01230	Biosynthesis of amino acids	C00025, C00041, C00049, aldoab, aldoca, aldocb, cbsb, glulb, gpt2, gpt2l, mat2ab, mat2b, pgam1a, pgam2
dre00410	beta-Alanine metabolism	C00049, C00386, aldh2.2, aldh3a1, aldh9a1a.1, aoc2, gad1a, gad2, smox, upb1
dre00330	Arginine and proline metabolism	C00025, aldh2.2, aldh3a1, aldh9a1a.1, aoc1, ckmb, ckmt1, gatm, oat, smox
dre00480	Glutathione metabolism	C00025, C00051, C00127, chac1, ggt5a, ggt5b, gpx4a, gsr, gstp1, gstp2
dre00270	Cysteine and methionine metabolism	C00041, C00049, C00051, ahcyl1, cbsb, enoph1, mat2ab, mat2b, mtap
dre00340	Histidine metabolism	C00025, C00049, C00386, aldh2.2, aldh3a1, aldh9a1a.1, aoc1, aspa
dre00250	Alanine, aspartate and glutamate metabolism	C00025, C00041, C00049, aspa, gad1a, gad2, glulb, gpt2, gpt2l
dre00260	Glycine, serine and threonine metabolism	C00049, C00719, alas1, aoc2, cbsb, gatm, pgam1a, pgam2, tdh
dre02010	ABC transporters	C00025, C00041, C00049, C00051, C00719, abca1a
dre04216	Ferroptosis	C00025, C00051, C00127, gpx4a, slc40a1, vdac3
dre00983	Drug metabolism	C00003, cdaa, gstp1, gstp2, nme2b.1, upb1
dre00430	Taurine and hypotaurine metabolism	C00025, C00041, gad1a, gad2, ggt5a, ggt5b
dre00190	Oxidative phosphorylation	C00003, cox5ab, ndufa5, ndufa6, ppa1b
dre00970	Aminoacyl-tRNA biosynthesis	C00025, C00041, C00049, rars2, yars
dre00220	Arginine biosynthesis	C00025, C00049, glulb, gpt2, gpt2l
dre01210	2-Oxocarboxylic acid metabolism	C00025, C00049, gpt2, gpt2l
dre00650	Butanoate metabolism	C00025, gad1a, gad2, hmgcs1

a) Only pathways including at least one metabolite are listed

Supplementary Table ST6.Quality Control information on RNA samples and sequencing parameters

Library ID	Sample Type	Treatment	Concentration (ng/µl)	260/280	260/230	RIN	SRA Accession	# of Read Pairs
RL_BCN1	Zebra fish brain	control	83.24	2.06	1.46	6.8	SRR8457166	44976258
RL_BCN2	Zebra fish brain	control	123.17	2.12	1.42	6.8	SRR8457167	53244586
RL_BCN3	Zebra fish brain	control	165.1	2.12	1.93	6.4	SRR8457164	44803945
RL_BCN4	Zebra fish brain	control	124.77	2.1	1.7	10	SRR8457165	45605938
RL_BCN5	Zebra fish brain	control	148.55	2.14	1.11	8.1	SRR8457170	51044940
RL_BCN6	Zebra fish brain	control	126.34	2.03	1.61	7.5	SRR8457171	56365118
RL_BCN7	Zebra fish brain	control	40.06	1.93	0.96	5	SRR8457168	54049013
RL_BACR1	Zebra fish brain	acrylamide	130.21	2.09	1.95	6.6	SRR8457169	42601423
RL_BACR2	Zebra fish brain	acrylamide	152.87	2.09	2.02	5.4	SRR8457172	44393341
RL_BACR3	Zebra fish brain	acrylamide	83.35	2.08	1.72	5.2	SRR8457173	43009514
RL_BACR4	Zebra fish brain	acrylamide	121.75	2.09	1.98	10	SRR8457162	50326552
RL_BACR5	Zebra fish brain	acrylamide	124.24	2.06	1.85	10	SRR8457163	45688169
RL_BACR6	Zebra fish brain	acrylamide	40.48	1.95	1.56	9.4	SRR8457160	44403598
RL_BACR7	Zebra fish brain	acrylamide	112.61	2.11	1.8	9.9	SRR8457161	49883027
